# Animal Welfare during Transport and Slaughter of Cattle: A Systematic Review of Studies in the European Legal Framework

**DOI:** 10.3390/ani13121974

**Published:** 2023-06-13

**Authors:** Svea Nicolaisen, Nina Langkabel, Christa Thoene-Reineke, Mechthild Wiegard

**Affiliations:** 1 Institute of Animal Welfare, Animal Behavior and Laboratory Animal Science, School of Veterinary Medicine, Freie Universität Berlin, 14163 Berlin, Germany; 2 Institute of Food Safety and Food Hygiene, Working Group Meat Hygiene, School of Veterinary Medicine, Freie Universität Berlin, 14163 Berlin, Germany

**Keywords:** bovines, animal welfare, abattoir, animal transport, stress

## Abstract

**Simple Summary:**

This systematic review addresses animal welfare-related aspects associated with the transport and slaughter of cattle. Before transport, the husbandry system and health status of cattle can have a major impact on animal welfare. At the abattoir, personnel may inflict stress on the animals when moving them to the lairage pens and to the stunning box. Constructional conditions and the resulting environmental effects have a major influence on stress induction as well. Stress can be assessed by both behavioural observations and measurement of physiological parameters. Rapid and effective stunning is an important welfare-related criterion. Some easily verifiable and detectable indicators of unconsciousness, such as immediate collapse after stunning, loss of rhythmic breathing, and loss of the corneal reflex are routinely monitored at the abattoir. Other aspects, such as measuring stress hormones in the blood or using an electrocardiogram during stunning, provide scientific information but are neither practically nor financially achievable in routine procedures. Expertise and training of drivers and abattoir personnel are an important contribution to stress reduction during handling of cattle and, therefore, to animal welfare during transport and slaughter and, finally, to meat quality.

**Abstract:**

Literature related to European transport and slaughter processes were included in this systematic review. The publication period is limited to the past twelve years since the European Animal Welfare Transport Regulation was enacted in 2009. Three different databases were used. The final screening resulted in the inclusion of 19 articles in this review. When handling cattle during transport and slaughter, personnel have an important impact and may inflict stress on the animals. Other factors, such as the group composition and health status prior to transport, can have a strong negative effect on animal welfare. At the abattoir, constructional conditions and the resulting environmental influences can have a negative impact on welfare as well. These include increased noise levels due to the lack of noise dampening and changing light conditions. Stress in cattle can be assessed, e.g., by measuring stress hormones or heart rate. Effective stunning is an important welfare-relevant step in the slaughtering process. Some signs of unconsciousness, such as immediate body collapse or absence of the corneal reflex, can be easily assessed. Expertise and continuous training of all personnel involved are important measures in stress reduction.

## 1. Introduction

Animal welfare is an important topic in current public debate [1]. Critical social comments on modern livestock farming and the associated production of food of animal origin have been increasingly brought up in recent years [2]. In a representative survey conducted in Germany, a large proportion of respondents rejected methods that caused suffering of animals during transport and slaughter [1]. Public debate focuses on animal welfare concerns and considerations of more animal-friendly husbandry and meat production systems [2]. Particularly regarding meat production, not only are aspects of animal husbandry of concern, but also animal transport, as well as the handling of live animals in the abattoir [3]. The German Federal Veterinary Chamber (Bundestierärztekammer e. V.) confirms that, regardless of the size of the abattoir, deficiencies in animal welfare can be identified between the processing steps of unloading and bleeding [4]. The results of this systematic review and the resulting compilation of animal welfare aspects served as the basis for an expert survey to determine which work steps during transport and slaughter are particularly relevant to animal welfare, and which of these relevant work steps could be effectively improved through employee training. The result of this expert survey in turn serves as the basis for a scientific project to produce training material for animal transporters and slaughterhouse employees.

### 1.1. Legal Background for Transport and Slaughter

Animal welfare during transport and slaughter is embodied in European regulations that are mandatory in all member states of the European Union (EU). Regulation (Reg.) (EC) No 1/2005 [5] sets requirements for the transport of live animals. In Germany, these are specified by national law [6]. Usually, cattle are transported from the farm to the abattoir for food production. The term ‘transport’ comprises the entire transport process from the loading of the first animal on the farm to unloading the last animal at the destination [5]. Transport of animals in an economic context over distances of more than 65 km must be carried out by approved transportation companies (Art. 10 & 11 of Reg. (EC) No. 1/2005) with approved long-distance transport vehicles (Art. 18 of Reg. (EC) No. 1/2005), and exclusively by transport personnel holding a certificate of competence (Art. 17 of Reg. (EC) No. 1/2005) [5]. Transport of animals is only allowed if they are fit for transport. The requirements are defined in Annex I of the regulation mentioned [5]. The fitness for transport must be assessed by the farmer as well as by the carrier personnel prior to loading. Animals not fit for transport must remain on the farm and, depending on the severity of the injuries, must get a provided treatment or be euthanized. Under certain conditions, defined in Annex II, Section I, Chapter VI of Reg. (EC) No 853/2004, such animals may also be emergency slaughtered [7]. Furthermore, bans on rough handling, such as kicking and hitting the animals during transport, are in place [5,8]. In addition, species-specific requirements regarding the conditions of the transport vehicle and the composition of the animal groups are defined [5].

Reg. (EC) No. 1099/2009 on the protection of animals at the time of killing regulates, amongst other aspects, the handling of animals during stunning and slaughter [9]. It lays down provisions on approved stunning methods for each respective animal species used for food production and other mandatory provisions for stunning and slaughtering. In Germany, the provisions of this regulation are implemented and supplemented by the Animal Welfare Slaughter Ordinance [10].

According to § 4a of the German Animal Protection Act, slaughter of animals is exclusively allowed after stunning, associated loss of consciousness and sensibility, and immediate exsanguination [8]. Exceptions to slaughter without stunning for religious reasons exist but require separate approval by the competent authority in each single case as an (usually temporary) exemption [8]. Stunning and slaughtering of animals should only be carried out by persons with a certificate of competence (Art. 21 of Regulation No. 1099/2009) [9]. Abattoirs slaughtering more than 1000 livestock units per year must designate an animal welfare officer to supervise animal welfare compliance during handling, stunning, and slaughtering. Additionally, standard operation procedures for animal handling must be created and personnel compliance must be supervised by animal welfare officers (Art. 17 of Reg. (EC) No 1099/2009) [9].

Compliance with these legal requirements is a prerequisite for animal welfare-compliant transport.

In addition to the legal regulations, supplementary scientific or instructional literature exists. The status of current scientific research on animal welfare measures in Europe during transport and slaughter is summarized in this article as a systematic literature review.

### 1.2. Aim of the Review

The aim of this review was to identify animal welfare-relevant aspects such as human-animal interactions that have been described and studied in the published literature on the transport and slaughter of cattle in Europe over the past twelve years.

## 2. Materials and Methods

This systematic literature review was conducted as part of the joint research project ‘eSchulTS2’ (development of target group-specific learning modules to improve animal welfare during the transport and slaughter of cattle and pigs). A systematic review provides an overview of the scientific literature on a specific topic using defined, comprehensible, and repeatable searching criteria [11]. In this type of systematic literature search, the procedure is guided by the Cochrane Guidelines Manual [12], which defines the standard methods for reviews, and the PRISMA-P (Preferred Reporting Items for Systematic Reviews and Meta-Analyses Protocols) protocol [13], which enables a transparent and replicable procedure.

Firstly, the search strategy was defined. This included the definition of search terms as well as inclusion and exclusion criteria for publications. The publication period was limited to 2010 to 2022, as the currently valid European regulation on the protection of animals at the time of killing (Reg. (EC) No. 1099/2009) was published in 2009 and has been mandatory since 2013 after the respective transition period [9]. Therefore, we decided to include studies from 2010, which were conducted within the scope of the Euro-pean legal requirements relating to the transport and slaughter of animals [5,9,10]. Abattoirs within the EU are also more comparable than abattoirs from other continents in terms of size and equipment. In order to assume comparability of transport conditions, only so-called short-distance transports, lasting less than 8 h according to the EU regulation, were considered, to which the same legal framework conditions apply. In addition, it is stipulated nationally in Germany that the transport duration of livestock animals for slaughter should not exceed 8 h [6]. Reviews and studies on stun-free or religion-motivated slaughter of cattle were not included.

Although it is well known that there is a variety of relevant literature available on the mentioned topic, comprising detailed manuals on animal-welfare-relevant instructions, exclusively literature from peer-reviewed journals was included in the evaluation in order to ensure scientific validity.

In the next step, the databases used for the literature search were determined. Important criteria were free accessibility and search mask availability in German or English. PubMed^®^ (National Library of Medicine, Bethesda, MD, USA) and Web of Science™ (IPAN GmbH, Munich, Germany) were selected as English-language databases, enabling access to very extensive literature collections with and without contractual ties to publishers. In addition, Livivo was selected as a database that provides German-language articles. In the following step, the search terms, including their combinations in both English and German (Table 1), were determined in order to capture the largest possible number of relevant articles.

**Figure 1:**
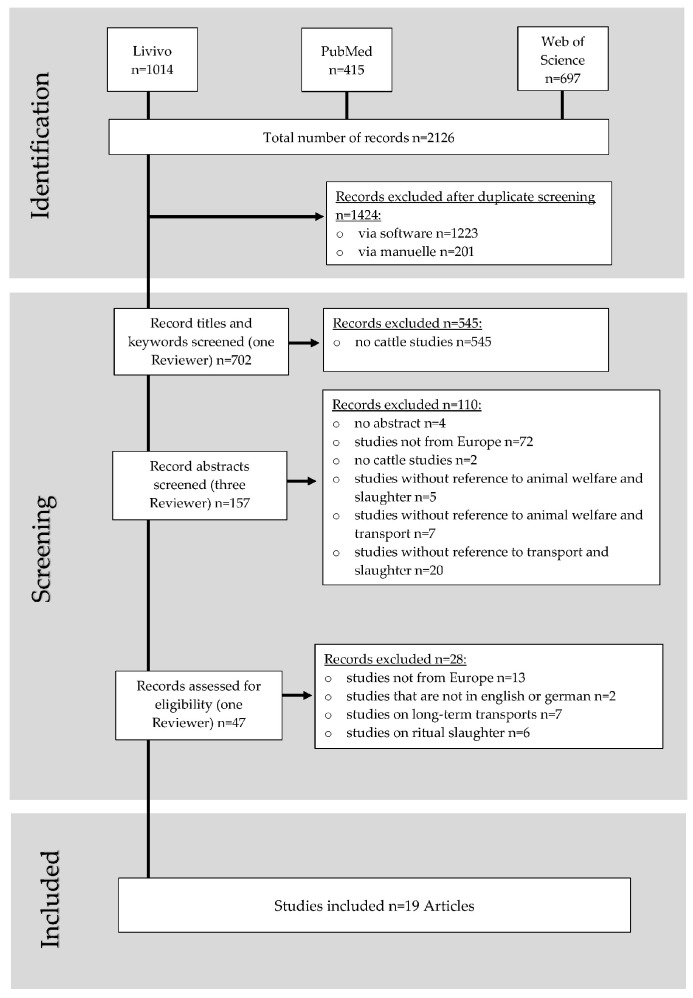
Flowchart of the review process.

A total of 2126 articles were initially retrieved from the three databases using the mentioned search terms. Removal of duplicates resulted in 702 publications (see Supplementary Material S3), which were checked for relevance by scanning both titles and keywords. The abstracts of the remaining 157 articles were assessed independently by three reviewers for relevance to content. The procedure was documented by means of a review protocol (see Supplementary Materials S1 and S2), divergent assessments were discussed within the reviewer group, and a final consensus was formed on which publications should be excluded or included. Finally, the full texts of 47 articles were assessed according to relevance, of which 19 were included in the final review (Figure 1, see Supplementary Material S4).

## 3. Results

The presentation of the results firstly includes two studies (both from Denmark) dealing with animal welfare- and transport-relevant aspects (Table 2). Secondly, 17 studies dealing with welfare concerns in the context of abattoir management, as well as stunning and slaughtering, within the last 12 years are included. Three studies originated from Sweden and France, respectively. Then, there were two studies each from Germany, Italy, the Czech Republic, and the Netherlands, and one study each from Switzerland, the United Kingdom, and Poland (Table 2). The order of the results presented below is based on the process along the slaughter chain.

### 3.1. Animal Welfare during Transport

Dahl-Pedersen et al. [14] studied dairy cows after transport. Due to a significant increase in the proportion of lame cows after transport, they recommend the exclusion of cows with pelvic asymmetries and non-specific hind leg lameness from transport [14]. Cows determined to be transported over relatively long distances were typically loaded onto a transport vehicle at the respective farms at night [14], several hours or more after the last milking routine [14]. Transport distances of more than 100 km and a high milk yield in early lactation (<100 day of lactation) were identified as risk factors for increased spontaneous milk leakage during transport [14]. Therefore, it is recommended to milk cows immediately before loading in order to avoid increased udder pressure, which is associated with pain and discomfort [14]. At the same time, Dahl-Pedersen et al. [14] suggested a need for further research on dairy cows that were legally considered fit for transport and, as a result, the further development of the concept of fitness for transport, as well as a more detailed consideration of the animal welfare implications of transporting cattle.

Herskin et al. [31] used a questionnaire survey to describe the knowledge of fitness for transport of dairy cows among livestock drivers. A total of 94% of the drivers reported that they were aware of the regulation on fitness for transport [31]. A total of 35% said that they had at least frequent doubts about the fitness of certain cows for transport, and only 52% of respondents answered correctly to specific questions about the legislation on fitness for transport [31]. Livestock drivers need additional training to improve the welfare of animals being transported [31].

### 3.2. Animal Welfare at Slaughter

The assessment of aspects of animal welfare in the context of slaughter already begins at the time point of unloading, as part of the ante mortem inspection, and can also continue in lairage pens at the abattoir, and during stunning. This includes assessment of the surrounding environment with its constructional conditions and environmental factors. In addition, the behaviour of the animals and physiological parameters, such as stress hormones, can be used for stress assessment.

#### 3.2.1. Animal Welfare at Lairage Pen and Driveway

Observing and analyzing tumbling, slipping, and the backward movements of cattle at abattoirs can provide insight into the connection between cattle handling and stress-related behaviours [15,16,17]. Tumbles were observed in about 1% of the animals in a study by Hultgren et al. [16], with heifers and bulls having a 3.2 times lower risk of falling than dairy cows. Cattle were most likely to move backwards in the driveway [16]. Backward movements of an animal may occur if distractions due to noise, the presence of people, conspecifics, or darkness are present in front of the animal [15,17]. Backward movements in the stunning box were observed significantly more often in bulls than in cows or heifers. It was pointed out that males were more difficult to handle and, thus, experienced a lower level of animal welfare at the abattoir [16].

Cattle to which electric prods were applied several times on their way to the stunning box showed elevated serum cortisol levels. The blood samples required were collected during exsanguination. However, these values could not be clearly statistically linked to specific stressors, such as the use of electric prods [18]. Bourguet et al. [15] evaluated the use of electric prods prior to slaughter and found a large variability in the number of electric shocks with an average of 7.1 ± 0.2 electric shocks per animal. According to their evaluation, the authors recommend the use of electric prodding only for the smallest possible proportion of animals on their way to the stunning box [15]. Sex can also have an impact on the expression of stress parameters [18]. For example, heifers driven forward had the highest cortisol levels (>90 ng/mL) compared to bulls and steers, while the type of use (crossbreeds, beef cattle, and dairy cattle) had no influence on the blood serum levels examined [18].

It was shown by electrocardiogram (ECG) that the animals’ heart rates were highest shortly before slaughter, probably due to the approach to the stunning box [19].

In lairage, vocalization due to hunger, social reasons, or pain, e.g., resulting from excessive pressure of fixation devices on the head or body in the stunning box, could be observed [15].

Bourguet et al. [15] observed long lairage times of cattle in a French abattoir (average 20.2 ± 1.9 h). Male animals had a shorter lairage time than females and were slaughtered first, as they were more actively perceived by the abattoir personnel [15].

Hultgren et al. [16] found no significant associations between animal behaviour and personnel-related action. This could suggest that the construction of the abattoir or events not directly targeting the animals, such as noise or people walking by, are more significant in affecting animal welfare than direct animal-human interactions [16].

It was observed that employees who felt more pressure in their personal work situation, e.g., lack of time, tended to be more forceful in handling the animals, which can lead to increased stress [17,20]. The presence of observers, however, can lead to an observer effect or bias. In this case, the employees adapted their behaviour and, as subjects of a scientific study, avoided actions contrary to animal welfare. However, in general, employees quickly become accustomed to the presence of observers and rough handling still occurred [16]. If employees at the abattoir are trained in gentle handling, this can reduce the use of rough handling methods, such as the unauthorized use of electric prods [17,20].

The floor in the lairage pens, driveway, and stunning box must be slip-resistant and maintained in such a way that the animals cannot slip or tumble [20]. In addition, the use of rubber mats can reduce the risk of injuries [20].

The side barriers of the stunning box should be adjustable to the size of the cattle in order to avoid several cattle entering simultaneously [20]. To reduce stress, the cattle should not visually perceive animals already slaughtered before entering the stunning box. This can be achieved, e. g., by installing visual protection devices [20].

Other stress factors at abattoirs are loud noises, which were tested using a sound level meter via smartphone app at an abattoir [21]. The recommended maximum value of 80 dB for humans and animals was frequently exceeded [21]. In three abattoirs, values of 88.8 dB, 92.3 dB, and 93.0 dB were measured on average, especially in the stunning area [21]. There was often a lack of sound isolation on walls and machines. The authors also suggested better training of personnel to reduce the noise level and, thus, stress to animals and humans [21]. Exceeding the recommended maximum noise level could lead to physiological reactions in humans and animals, such as stress by excitation of the nervous system [21].

Other environmental factors include ventilation and light. Depending on the type of ventilation, moving elements such as ventilator blades and the light effects of their shadows on the floor or walls can cause irritation to the animals, which should be eliminated [20]. Furthermore, the combination of excessive light intensity and draught can cause the animals to flinch, especially in front of the entrance to the stunning box [20].

#### 3.2.2. Animal Welfare during Stunning and Bleeding

Criteria for the correct stunning procedure, using penetrating captive bolt devices, include deviations from the optimal shot position in centimeters, the entrance angle of the shot, and if a re-shot is necessary [22,32]. The optimal position for penetrating captive bolt devices in cattle is oriented towards the intersection of two imaginary connecting lines between the center of the horn buds and the center of the contralateral eye [22,32]. With an angle of 90° to the skull surface, a maximum angular and lateral deviation of 10°, and 0 to 2.5 cm from the optimal touch down point, the brainstem should be effectively damaged with a high probability [22]. The number and placement of shots can serve as an indirect quality control measure of the stunning procedure [22]. Macroscopic post-mortem examination of bovine skulls can also be used as a tool to assess possible reasons for insufficient stunning by determining the area of the brain destroyed by the penetrating captive bolt [23] and the number and precision of the shots [22,32]. This method of verification can be carried out on frozen cattle skulls at the abattoir itself if a freezer and band saw are available [23].

Von Wenzlawowicz et al. [24] found that shot accuracy is less critical when powerful penetrating captive bolt devices are used. Heavy captive bolt devices with rounded top surfaces have an increased risk of being shot inaccurately or not perpendicular to the skull surface, which can lead to insufficient stunning, especially in heavy cattle > 600 kg [24].

Sticking for exsanguination within less than 60 s after stunning was more important in a neck cut than in a thoracic cut, since the latter leads to greater blood loss in relation to the body weight within 60 s [24]. In situations in which stunning conditions are not optimal, a well-executed sticking by thoracic cut can reliably prevent cattle from re-awakening after a captive bolt shot [24].

The absence of active behaviour, body functions, and various reflexes can be assessed as indicators of consciousness and, thus, of the effectiveness of stunning.

One sign of ineffective stunning after using a captive bolt device is the animal’s attempt to rear from an abnormal position. This reaction is caused by the righting reflex [25]. These movements are purposive but often difficult to differentiate from involuntary movements, such as paddling [26]. However, the immediate collapse of an animal after a correctly executed captive bolt shot is described as a good indicator of unconsciousness after stunning, as it is easily visible [26]. Type of stunning and fixation of the animal must be considered since mechanical stunning leads to an immediate collapse, while head or body fixation impedes indicator assessment [26].

The following reflexes, listed in cranial to caudal order, were examined in further studies: threat reflex, lid reflex, palpebral reflex, corneal reflex, and righting reflex.

The threat reflex is provoked by the hand being moved quickly towards the cattle’s eyes, which leads to closing the eyes or pulling back of the head in conscious animals [25]. The threat reflex is lost at an early and incomplete stage of stunning when both eyelid and corneal reflexes may still be present. Therefore, although the test may have good sensitivity, it is recommended not to be used alone to assess the stunning effect and further research in other contexts and on other species is recommended [26].

In order to test the eyelid or palpebral reflex, the eyelashes or medial corner of the eye are touched [25,27]. The physiological response in insufficiently stunned animals is to close the eyelids [15,25]. The eyelid reflex is lost before the corneal reflex [15]. When testing the corneal reflex, the cornea is touched directly, and the eye should not show any reaction if the stunning was successful [25]. In contrast to the corneal reflex, standardization and interpretation of the presence or absence of the eyelid reflex may be more difficult [26].

The loss of the corneal reflex is considered an indicator of deep unconsciousness [15]. It is considered a valid indicator but should be interpreted together with other indicators of unconsciousness. It must be considered that injuries, e.g., on the ocular surface, can lead to the loss of the corneal reflex as well [15].

The withdrawal reflex is provoked by forcefully pressing two fingers or a tool into the nasal septum or the tips of the ears to trigger immediate head withdrawal [25].

Eye following is a clear sign of consciousness, as an object is visually fixed and observed [15]. After a successful captive bolt shot and collapse of the animal, the eyes are rigid and wide open, with immobile bulbi and eyelids [26]. If movements of the eye bulbi occur, this may indicate that parts of the brainstem and cortex are still intact and, thus, stunning was not sufficient [26]. Bulbus rotation with visible sclerae can be seen as an indication of ineffective stunning or a sign of incomplete loss of consciousness [15]. Nystagmus—rapid movements from side to side—is an indication of ineffective stunning as well [28].

The absence of rhythmic breathing is also mentioned as an indicator of unconsciousness [26]. This refers to focused inhalation and exhalation and should not be confused with gasping. The respiratory muscles are innervated via the medulla oblongata, which is located in the lower part of the brainstem. The rhythm of breathing is stimulated by the reticular formation [26]. Contrarily, gasping manifests as vigorous inspiration and is triggered by brain ischemia or hypoxia [26]. If uncertain, breathing can be visualized by the fogging of a mirror placed in front of the animal’s nose [29].

Successful stunning is indicated by immediate collapse after the stunning shot, followed by a phase of tonic and then clonic muscle contractions, loss of rhythmic breathing, threat, and corneal reflexes, as well as the absence of vocalization, and fixed eye bulbi [28]. Most of the indicators can be assessed visually and can therefore be easily used to check stunning success in abattoirs [30].

## 4. Discussion

The Animal Health and Welfare Committee of the European Food Safety Authority (EFSA) identified and characterized a total of 40 risks to cattle welfare during slaughter [33]. In total, 39 of the 40 risks were caused by humans and are mainly associated with lack of skills or fatigue [33]. The EFSA recommends preventive measures, such as livestock driver training to encourage cautious driving with regard to animal welfare during transport, or training of abattoir staff in the handling of animals [3,33]. In contrast to the human-animal interactions, other aspects such as the constructional conditions of a livestock transport vehicle or those of the abattoir, can only be affected indirectly by individuals or with a financial effort.

### 4.1. Transport

One study shows that there is a need for further research on dairy cows and their assessment of fitness for transport [14]. An assessment of cattle fitness for transport is mandatory, otherwise loading is not allowed. Livestock transport drivers must have the necessary knowledge for such an assessment. This can be particularly challenging in dairy cows because, unlike healthy beef cattle, they are usually slaughtered for reasons of poor health, i.e., claw, udder, or metabolic disorders, and performance, such as fertility problems, which are the most common reasons for animal disposals [34]. It is not unusual for drivers, farmers, and veterinarians to disagree on the assessment of the fitness for transport of individual animals. However, the assessment of the driver is important, as they are responsible for compliance with the legal provision. The animals are usually presented to a veterinarian for assessment only when they are unloaded at the abattoir. Herskin et al. [31] concluded that drivers need additional training and assessment tools to optimize animal welfare during transport. Transport personnel and their interactions with cattle play a very important role along the entire transport process, which consists of planning and preparing the transport, loading the animals, the transport itself, and unloading the animals at the abattoir [35]. For this reason, animals must be transported by trained personnel holding a certificate of competence [5] to ensure proper assessment of fitness for transport, as well as loading and unloading [33]. Livestock transport drivers should be aware of the needs, perception, and sensibilities of animals, and should handle them in the proper way [36]. Not all participants of training courses already have experience in handling livestock, which emphasizes the necessity of adequate and sufficient training, e.g., when loading or driving the animals [37]. Topics to be covered during training are: basic knowledge of the species to be transported, requirements for the livestock transport vehicle and loading equipment, the conduct of livestock transport, the effects of driving on animal welfare, first aid procedures for the animal, and aspects of work safety [5]. Transport companies must plan the transport carefully, which includes taking into account the actual transport route, driving, and break times, as well as information on weather and traffic conditions. In addition to the above-mentioned fitness assessment, the stocking density of the truck has to be determined according to the legal requirements [5]. Critically, none of the studies adequately investigated the effects of driving style on short-distance livestock transport. The literature merely mentions poor road conditions leading to balance problems in cattle [14], but the way in which speed, starting, and braking behaviour affects cattle is not explored in depth. In the field of long-distance livestock transport, the behaviour and heart rate of cattle during starting and braking processes was investigated and the authors showed that lying cattle stood up during starting and braking and that the heart rate increased [38]. A study from Australia found that shifting gears, starting, and braking can lead to anxious and tensed behaviour in cattle [39]. Other studies focused on driving style in sheep and pig transport [40,41].

Unfortunately, our search did not reveal any studies with other important animal welfare aspects, such as the condition of livestock transport vehicles or heat stress of cattle during transport. Livestock transport vehicles must be designed to avoid suffering, injury, or exposure to extreme temperatures, allow for easy cleaning and disinfection, exhibit non-slip floor surfaces, and be equipped with a light source to supervise the animals [5]. Careful transport planning, considering weather conditions, can help to avoid extreme temperatures in the transporter, while driving at night during summer heat periods or using forced ventilation should also be considered [42]. In this context, transport planning should also include detailed route and time planning.

No studies from Europe in the examined time period were found which dealt with the short-distance transport of pregnant cows or the group composition of cattle during transport. Reg. (EC) No 1/2005 further specifies that groups of animals unfamiliar to each other should not be mixed [5]. It is recommended that the social environment of the cattle be maintained as far as possible during transport [5,42]. Husbandry conditions have an impact on fear reactions, as intensively reared cattle familiar with human contact will usually react with less stress to a human presence than extensively reared cattle [43]. For the latter animals, the transport itself and the confrontation with humans will probably be more stressful [43]. Handling extensively reared animals is usually more difficult and, therefore, requires qualified and animal-welfare-conscious personnel [44]. Regarding pregnant cattle, Reg. (EC) No 1/2005 requires that they be given 10% more space during transport and animals in which 90% or more of the expected gestation period has elapsed or which have given birth in the last seven days may not be transported [5]. The maximum height of vehicles in Germany is 4 m [45]. Multi-deck road vehicles present a particular challenge in terms of space above the animals’ heads. In a study in which pregnant heifers were assessed during transport, no skin abrasions had been observed with a large distance (40 cm) to the ceiling [46]. The authors recommended keeping a distance of more than 20 cm between shoulder height and the ceiling to avoid injuries and additional stress in cattle [46].

### 4.2. Slaughter

Different studies show that vocalization and fearful behaviour in cattle, e.g., aggression, backward movements, and slipping, can be an expression of stress [15,16,17]. Loud vocalization in cattle is rare in a calm herd but should not be dismissed as insignificant in the context of transport and stress, as vocalization often seems to be associated with separation, pain, or anxiety [47]. Grandin [48] observed in abattoirs that when 95% or more of the cattle are forced to move with an electric prod due to backward movements or refusing to move forward, vocalization following the use of the electric prod increased significantly. The study also showed that in abattoirs where personnel did not make a lot of noise, like shouting or whistling, the lowest number of problems moving cattle to the stun box occurred [48]. The assessment of vocalization in commercial cattle abattoirs can be used to identify problems in the plant [48].

Noise is caused by the cattle and staff, as well as by technical equipment in the adjacent abattoir. The volume of surrounding noise can be determined by means of a sound level meter and should be evaluated regularly for animal and staff protection reasons. Noise reduction through sound damping is technically possible but often difficult to implement in practice due to hygiene requirements (e.g., easy-to-clean surfaces). Once an abattoir has been built, constructional conditions can often only be changed with a financial effort.

Stress hormones, such as cortisol, can provide information on both acute (e.g., measured in saliva or blood) and chronic (measured in hair) stress [49,50]. The glucocorticoid cortisol is secreted by the hypothalamic-pituitary-adrenal axis and can activate catabolic metabolic processes [51]. For the interpretation of cortisol levels, the diurnal pattern of cortisol release has to be taken into account, as well as the potentially stressful situation. Catecholamines are synthesized, particularly in the adrenal medulla. They serve as neurotransmitters and have an evolutionary-biological effect, primarily on the fight-or-flight response by sympathetic activation [51]. Probst et al. [18] and Bourguet et al. [19] have shown that the cortisol levels and heart rate of cattle were increased during the approach to the stunning box. An Australian study [52] showed that plasma cortisol levels (blood taken at ventral neck incision) were increased in cases when cattle turned their head down, vocalized a lot, and when abattoir personnel pushed the animals extensively.

Determining parameters such as cortisol and catecholamine levels, as well as heart rate, could be meaningful indicators of stress in animals but are not routinely feasible and implementable at the abattoir. Blood or urine sampling from the lairage pens seems to be, likewise, possible and useful mainly in the context of scientific studies.

In the studies on stunning [22,23,24,25,32], only the method of stunning with captive bolt devices was mentioned. Before stunning, the slaughter personnel must ensure that the captive bolt device is in proper condition [6]. Its area of use and maintenance guidelines (cleaning and functional inspection at least once per day) are laid down in Reg. (EC) No 1099/2009 [9]. Captive bolt devices are regulated by Directive 2006/42/EC and must have a CE mark [53]. Random sampling of the skulls of slaughtered cattle can provide information on the shot position, the angle of the shot, and the number of stun shots fired [23]; another possibility is monitoring via video supervision for retrospective assessment. To illustrate the optimal shot position, the assessment of macroscopic lesions on bovine skulls can serve as training material for slaughter personnel [23]. The number of shots used versus animals slaughtered per day should also be checked regularly, as this provides information on how often additional bullets had to be fired.

Animals must be stunned properly before slaughter and loss of consciousness has to be maintained [9]. Indicators that serve to immediately verify the effectiveness of stunning, such as reflex and behaviour assessment, are very important. Verhoeven et al. [25] validate the interpretation of reflexes using electroencephalography (EEG) electrodes. After captive bolt stunning, the cattle showed no reflexes and the EEG recordings confirmed that the animals were unconscious [25]. Since EEG administration is too time-consuming for routine use at the abattoir, the recording was only carried out for scientific purposes and validation of procedures. The loss of reflexes can provide information on the state of consciousness after mechanical stunning and, in contrast to an EEG, can also be used time-efficiently in routine procedures at the abattoir. The immediate collapse after correct stunning is an easily visible indicator well-suited for verification, provided that the animals are not fixed in the stunning box [26].

In general, no reflex should be assessed alone regarding unconsciousness. The EFSA [3] recommends that the combinations of immediate collapse, loss of muscle tone, loss of breathing, presence of tonic spasms, loss of eyelid and corneal reflex, fixed eyes, and absence of vocalization should be checked to verify unconsciousness. If stunning success is questionable, re-stunning should be applied immediately [3].

In order to obtain an economic benefit from slaughtered cattle, the carcass must be in good condition. Bruising is caused by vascular ruptures, which can occur as a result of being hit or beaten by another animal, bumped during transport, or due to forceful human-animal interactions [54]. Animal welfare officers at the abattoir must be trained to recognize bruises and injuries on the living animal and on the carcass. In addition, certain metabolic processes, such as glycolysis, can change the quality of the meat and dark, firm, dry (DFD) meat can result. DFD meat is formed when the glycogen reserves in the muscle have already been largely depleted due to prolonged stressful situations in conjunction with exhaustion before slaughter. After slaughter, if glycogen reserves are too low, only a small amount of lactic acid can be formed via glycolysis and the pH value in the muscles drops only slightly, resulting in a high final pH value, which is associated with DFD meat [55]. Triggers for such stressful situations can be the common stabling of female and male cattle, affecting factors of the transport itself (temperature and driving style), the duration of unloading, and the skill level of the driver [56].

## 5. Conclusions

This systematic literature review provides an overview of the literature from the last twelve years on animal welfare during transport and slaughter of cattle within the legal framework of the EU. The aspects identified as relevant for the improvement of animal welfare, as well practically useful in terms of implementation, should be incorporated into the development of training material in order to counteract animal welfare concerns.

The effects of driving style and road conditions, specifically, on cattle should be considered and further scientific research would be desirable in this regard. Animal transporters should, therefore, receive extensive training in analyzing their own driving style and its effects on the animals being transported. At the same time, an expansion of the training regarding the assessment of the transport ability of cattle is desirable and a standardization of the assessment is necessary.

Gentle handling of animals has a major impact on animal welfare at any time of transport, or during lairage and slaughter process. According to the EFSA, the main threats to animal welfare are related to a lack of personnel skills or training, resulting in improper handling and poorly designed facilities [3]. The EFSA concluded that this lack of skills or training poses a serious animal welfare problem.

The availability of appropriate training material, with particular attention to the educational background and different language skills of animal transporters and slaughterhouse staff, is a key factor and influencing opportunity for improving animal welfare during transport and slaughter. This review of the available literature is therefore a contribution to the identification of animal-welfare-relevant and educable training sessions.

## Figures and Tables

**Table 1:** Search term combinations for the systematic literature review on animal welfare during transport and slaughter of cattle.

Language	Search Term Combinations				
English	(Cattle OR Bovine)	AND	(Animal Welfare OR Welfare)	AND	(Slaughter OR Slaughterhouse OR Abattoir OR Lairage OR Bleeding OR Stunning)
	(Cattle OR Bovine)	AND	(Animal Welfare OR Welfare)	AND	(Transport)
German	(Rind)	AND	(Tierwohl OR Tierschutz)	AND	(Schlachtung OR Schlachthaus OR Schlachtbetrieb OR Schlachten OR Schlachthof OR Wartestall OR Tötung OR Betäubung OR Entblutung)
	(Rind)	AND	(Tierwohl OR Tierschutz)	AND	(Lebendtiertransport OR Tiertransport OR Viehtransport OR Transport)

**Table 2:** Studies (*n* = 19) which were included in the results. These studies are from Europe, focus on cattle welfare, and have done research on slaughter or transport.

	Selection Process of the Included Articles				
Reference	Animal Species	Country of Origin of Study	Slaughter Reference	Transport Reference	Animal Welfare Reference
Dahl-Pedersen et al., 2018 [14]	Dairy Cows	Denmark	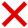	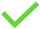	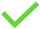
Bourguet et al., 2011 [15]	Cattle	France	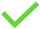	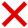	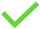
Hultgren et al., 2014 [16]	Cattle	Sweden	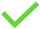	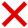	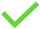
Hultgren et al. 2020 [17]	Cattle	Sweden	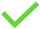	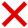	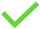
Probst et al., 2014 [18]	Cattle	Switzerland	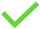	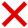	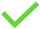
Bourguet et al., 2010 [19]	Cattle	France	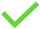	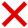	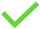
Disanto et al., 2014 [20]	Cattle	Italy	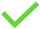	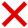	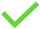
Iulietto et al., 2018 [21]	Cattle, Pig	Italy	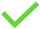	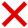	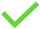
Fries et al., 2012 [22]	Cattle	Germany	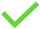	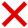	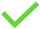
Grist, 2019 [23]	Cattle	United Kingdom	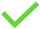	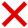	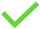
von Wenzlawowicz et al., 2012 [24]	Cattle, Pig	Germany	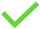	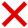	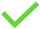
Verhoeven et al., 2016 [25]	Veal Calves	Netherlands	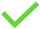	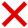	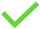
Terlouw et al., 2016 [26]	Cattle	France	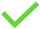	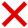	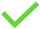
Verhoeven et al., 2015 [27]	Cattle, Pig	Netherlands	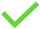	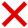	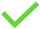
Atkinson et al., 2013 [28]	Cattle	Sweden	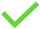	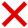	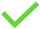
Borzuta et al., 2019 [29]	Cattle, Sheep, Pig, Poultry	Poland	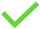	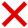	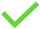
Vecerek et al., 2020 [30]	Cattle	Czech Republic	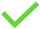	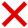	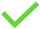
Herskin et al., 2017 [31]	Cattle, Pig	Denmark	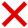	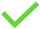	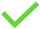

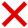
 = is not addressed in the study 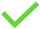 = is addressed in the study.

## Data Availability

Data sharing not applicable.

## Supplementary Materials

The following supporting information can be downloaded at: https://www.mdpi.com/article/10.3390/ani13121974/s1, S1: PRISMA 2020 Checklist for Nicolaisen et al. “Animal welfare during transport and slaughter of cattle: a systematic review of studies in the European legal framework” [57]; S2: Search protocol; S3: List of all records; S4: List of included publications.
