# Supplementary material for: Animal Welfare during Transport and Slaughter of Cattle: A Systematic Review of Studies in the European Legal Framework
Source: Animals (Basel). 2023 Jun 13;13(12):1974. doi: 10.3390/ani13121974 (PMC10295209; doi:10.3390/ani13121974)
Supplement: Supplementary file 1 [file animals-13-01974-s001.zip › S2.pdf]

Animal welfare during transport and slaughter of cattle: a systematic review of studies in the European legal framework

S2: Search Protocol Systematic Review

Search term combinations for Databases Livivo, Pubmed® and Web of Science™:

- (cattle OR bovine) AND (animal welfare OR welfare) AND (slaughter OR slaughterhouse OR abattoir OR lairage OR bleeding OR stunning)
- (cattle OR bovine) AND (animal welfare OR welfare) AND (transport)
- (Rind) AND (Tierwohl OR Tierschutz) AND (Schlachtung OR Schlachthaus OR Schlachtbetrieb OR schlachten OR Schlachthof OR Warte-stall OR Tötung OR Betäubung OR Entblutung)
- (Rind) AND (Tierwohl OR Tierschutz) AND (Lebendtiertransport OR Tiertransport OR Viehtransport OR Transport).

Database Livivo

First Search 11.08.2020

Advanced Search	Settings
Years	2010 until date of search
Subjects	-
Document Types	Article
Language	German or English
Database	-
Related Terms	-

Repeated Search 14.02.2022

Advanced Search	Settings
Years	2020 until date of search
Subjects	-
Document Types	Article
Language	German or English
Database	-
Related Terms	-

Database Pubmed®

First Search 14.08.2020

Advanced Search	Settings
Years	2010 until date of search
Text availability	-
Article attribute	-
Document Types	Case Reports; Classical Article; Clinical Study; Clinical Trial Protocol; Clinical Trial, Phase I; Clinical Trial, Phase II; Clinical Trial, Phase III; Clinical Trial, Phase IV; Clinical Trial, Veterinary; Comparative Study; Controlled Clinical Trial; Corrected and Republished Article; Dataset; English

	Abstract; Evaluation Study; Government Publication; Guideline; Interactive Tutorial; Introductory Journal Article; Journal Article; Legislation; Multicenter Study; Observational Study; Observational Study, Veterinary; Practice Guideline; Pragmatic Clinical Trial; Preprint; Twin Study; Validation Study
Publication date	-
Language	German or English

Repeated Search 14.02.2022

Advanced Search	Settings
Years	2020 until date of search
Text availability	-
Article attribute	-
Document Types	Case Reports; Classical Article; Clinical Study; Clinical Trial Protocol; Clinical Trial, Phase I; Clinical Trial, Phase II; Clinical Trial, Phase III; Clinical Trial, Phase IV; Clinical Trial, Veterinary; Comparative Study; Controlled Clinical Trial; Corrected and Republished Article; Dataset; English Abstract; Evaluation Study; Government Publication; Guideline; Interactive Tutorial; Introductory Journal Article; Journal Article; Legislation; Multicenter Study; Observational Study; Observational Study, Veterinary; Practice Guideline; Pragmatic Clinical Trial; Preprint; Twin Study; Validation Study
Publication date	-
Language	German or English

Database Web of Science™

First Search 18.08.2020

Advanced Search	Settings
Years	2010 until date of search
Categories	-
Document Types	Article
Language	German or English
Publishers	-

Repeated Search 16.02.2022

Advanced Search	Settings
Years	2020, 2021, 2022
Categories	-
Document Types	Article
Language	German or English
Publishers	-
