# Supplementary material for: Animal Welfare during Transport and Slaughter of Cattle: A Systematic Review of Studies in the European Legal Framework
Source: Animals (Basel). 2023 Jun 13;13(12):1974. doi: 10.3390/ani13121974 (PMC10295209; doi:10.3390/ani13121974)
Supplement: Supplementary file 1 [file animals-13-01974-s001.zip › S3.pdf]

Animal welfare during transport and slaughter of cattle: a systematic review of studies in the European legal framework

S3: List of all records

No.	Authors	Year	Titel	Journal	DOI
1.	S. K. J. Hultgren J, Babol J, Berg C	2022	Animal Welfare and Food Safety When Slaughtering Cattle Using the Gunshot Method	Animals (Basel)	10.3390/ani12040492
2.	V. K. Vecerek, J.; Voslarova, E.; Volfova, M.; Machovcova, Z.; Konvalinova, J.; Vecerkova, L.	2020	The Impact of Deviation of the Stun Shot from the Ideal Point on Motor Paralysis in Cattle	Animals (Basel)	10.3390/ani10020280
3.	V. Vecerek; J. Kamenik; E. Voslarova; L. Vecerkova; Z. Machovcova; M. Volfova; J. Konvalinova	2020	The occurrence of reflexes and reactions in cattle following stunning with a captive bolt at the slaughterhouse	Anim Sci J	10.1111/asj.13373
4.	J. Stojkov; M. A. G. von Keyserlingk; T. Duffield; D. Fraser	2020	Fitness for transport of cull dairy cows at livestock markets	J Dairy Sci	10.3168/jds.2019-17454
5.	J. Stojkov; M. A. G. von Keyserlingk; T. Duffield; D. Fraser	2020	Management of cull dairy cows: Culling decisions, duration of transport, and effect on cow condition	Journal of dairy science	
6.	A. Sharma; C. Schuetze; C. J. C. Phillips	2020	The Management of Cow Shelters (Gaushalas) in India, Including the Attitudes of Shelter Managers to Cow Welfare	Animals	10.3390/ani10020211
7.	J. B. Schwantes; P. Quevedo; M. F. D'Avila; M. B. Molento; D. A. S. Graichen	2020	Fasciola hepatica in Brazil: genetic diversity provides insights into its origin and geographic dispersion	Journal of Helminthology	10.1017/s0022149x19000774
8.	L. Schneider; N. Volkmann; N. Kemper; B. Spindler	2020	Feeding Behavior of Fattening Bulls Fed Six Times per Day Using an Automatic Feeding System	Front Vet Sci	10.3389/fvets.2020.00043
9.	A. Sandelin	2020	Field trial to evaluate the effect of an intranasal respiratory vaccine protocol on bovine respiratory disease incidence and growth in a commercial calf rearing unit	BMC veterinary research. 2020 Dec., v. 16, no. 1	
10.	S. M. Roche; D. L. Renaud; R. Genore; D. A. Shock; C. Bauman; S. Croyle; H. W. Barkema; J. Dubuc; G. P. Keefe; D. F. Kelton	2020	Canadian National Dairy Study: Describing Canadian dairy producer practices and perceptions surrounding cull cow management	Journal of dairy science	

11.	M. Rice; L. M. Hemsworth; P. H. Hemsworth; G. J. Coleman	2020	The Impact of a Negative Media Event on Public Attitudes Towards Animal Welfare in the Red Meat Industry	Animals: an open access journal from MDPI	
12.	B. B. Padalino, R.; Tullio, D.; Zappaterra, M.; Costa, L. N.; Bozzo, G.	2020	Protection of Animals during Transport: Analysis of the Infringements Reported from 2009 to 2013 during On-Road Inspections in Italy	Animals (Basel)	10.3390/ani10020356
13.	M. V. Noriega; G. C. M. de la Lama	2020	Implications, trends, and prospects for long-distance transport in cattle. Review	Revista Mexicana De Ciencias Pecuarias	10.22319/rmcp.v11i2.4767
14.	Y. Z. Njisane; F. E. Mukumbo; V. Muchenje	2020	An outlook on livestock welfare conditions in African communities - A review	Asian-Australasian Journal of Animal Sciences	10.5713/ajas.19.0282
15.	L. Navone; R. Speight	2020	Enzymatic removal of dags from livestock: an agricultural application of enzyme technology	Applied microbiology and biotechnology	
16.	N. Nani; Z. Rania Samir	2020	Biochemical and immunological investigation of fascioliasis in cattle in Egypt	Veterinary World, Vol 13, Iss 5, Pp 923-	
17.	D. Mouzo; R. Rodriguez-Vazquez; J. M. Lorenzo; D. Franco; C. Zapata; M. Lopez-Pedrouso	2020	Proteomic application in predicting food quality relating to animal welfare. A review	Trends in Food Science & Technology	10.1016/j.tifs.2020.03.029
18.	T. Meschik; B. Senft; A. Rabitsch; J. Troxler; J. Baumgartner	2020	Cattle exports from the European Union to non-EU countries: current status and challenges	Wiener Tierarztliche Monatsschrift	
19.	D. M. Meléndez; S. Marti; D. B. Haley; T. D. Schwinghamer; K. S. Schwartzkopf-Genswein	2020	Effect of transport and rest stop duration on the welfare of conditioned cattle transported by road	PloS one	
20.	J. W. McFadden	2020	Review: Lipid biology in the periparturient dairy cow: contemporary perspectives	Animal: an international journal of animal bioscience	
21.	C. Masmeijer	2020	Effects of glycerol-esters of saturated short- and medium chain fatty acids on immune, health and growth variables in veal calves	Preventive veterinary medicine. 2020 May, v. 178	
22.	M. Marahrens; I. Schwarzlose; J. Knöll; A. Biermann; D. Möhrlein; J. Hartung	2020	Aktuelle Untersuchungen zum Tierschutz bei der Betäubung von Schlachtschweinen und Rindern<br><br>[Keynote]		
23.	L. Magrin; F. Gottardo; G. Cozzi; C. Bergsten	2020	Wider slot in pens with fully slatted rubber mat flooring for fattening bulls: Effects on animal hygiene, health and welfare	Livestock Science	10.1016/j.livsci.2020.103989

24.	L. Magrin; M. Brscic; L. Armato; B. Contiero; A. Lotto; G. Cozzi; F. Gottardo	2020	Risk factors for claw disorders in intensively finished Charolais beef cattle	Preventive Veterinary Medicine	10.1016/j.prevetmed.2019.104864
25.	D. E. Lowe; A. W. Gordon; F. O. Lively	2020	Effect of overlaying rubber on fully slatted concrete floors on hoof health and lying postures in finishing dairy-origin bulls offered two contrasting diets	Animal	10.1017/s1751731119002702
26.	N. Losada-Espinosa; G. C. Miranda-De la Lama; L. X. Estevez-Moreno	2020	Stockpeople and Animal Welfare: Compatibilities, Contradictions, and Unresolved Ethical Dilemmas	Journal of Agricultural & Environmental Ethics	10.1007/s10806-019-09813-z
27.	J. F. Lindahl; C. E. Vrentas; R. P. Deka; R. A. Hazarika; H. Rahman; R. G. Bambal; J. S. Bedi; C. Bhattacharya; P. Chaduhuri; N. M. Fairoze; R. S. Gandhi; J. P. S. Gill; N. K. Gupta; M. Kumar; S. Londhe; M. Rahi; P. K. Sharma; R. Shome; R. Singh; K. Srinivas; B. B. Swain	2020	Brucellosis in India: results of a collaborative workshop to define One Health priorities	Trop Anim Health Prod	10.1007/s11250-019-02029-3
28.	R. A. Laven; M. C. Jermy	2020	Measuring the torque required to cause vertebral dislocation in cattle tails	N Z Vet J	10.1080/00480169.2019.1685019
29.	H. Khaneghahi Abyaneh; A. Dabaghian; M. Rezaeigolestani; D. Amanollahi	2020	Compliance with OIE animal welfare standards in slaughterhouses in Tehran Province, Iran: An introductory survey	J Appl Anim Welf Sci	10.1080/10888705.2019.1577735
30.	N. N. Jonsson; H. J. Ferguson; H. H. C. Koh-Tan; C. A. McCartney; R. C. Cernat; E. M. Strachan; W. Thomson; T. J. Snelling; C. D. Harvey; I. Andonovic; C. Michie; R. J. Wallace	2020	Postmortem observations on rumen wall histology and gene expression and ruminal and caecal content of beef cattle fattened on barley-based rations	Animal	10.1017/s1751731119002878
31.	J. C. Imlan; U. Kaka; Y. M. Goh; Z. Idrus; E. A. Awad; A. A. Abubakar; T. Ahmad; H. N. Q. Nizamuddin; A. Q. Sazili	2020	Effects of Slaughter Knife Sharpness on Blood Biochemical and Electroencephalogram Changes in Cattle	Animals (Basel)	10.3390/ani10040579
32.	R. M. Hyde; M. J. Green; V. E. Sherwin; C. Hudson; J. Gibbons; T. Forshaw; M. Vickers; P. M. Down	2020	Quantitative analysis of calf mortality in Great Britain	Journal of dairy science	

33.	J. Hultgren; K. A. Segerkvist; C. Berg; A. H. Karlsson; B. Algers	2020	Animal handling and stress-related behaviour at mobile slaughter of cattle	Preventive Veterinary Medicine	10.1016/j.prevetmed.2020.104959
34.	A. Hopker; N. Pandey; J. Goswami; S. Hopker; R. Saikia; A. Jennings; D. Saikia; N. Sargison; R. Marsland	2020	Colostrum provision and care of calves among smallholder farmers in the Kaziranga region of Assam, India	PloS one	
35.	L. Herve	2020	To what extent does the composition of batches formed at the sorting facility influence the subsequent growth performance of young beef bulls? A French observational study	Preventive veterinary medicine. 2020 Mar., v. 176	
36.	M. Hässig; S. Betschart	2020	Ethik in der Nutztierhaltung: Die Rolle der Tierärzte im Tierschutz am Beispiel Rind	Schweizer Archiv fur Tierheilkunde	
37.	A. Fuseini; T. G. Knowles	2020	The ethics of Halal meat consumption: preferences of consumers in England according to the method of slaughter	The Veterinary record	
38.	K. D. Ferreira; A. C. Furtado; H. P. Flores; P. R. D. de Oliveira; A. G. Goncalves; D. M. de Oliveira	2020	Cattle loading rates in different truck models and their relationship with bruises on bovine carcasses	Ciencia Rural	10.1590/0103-8478cr20190819
39.	D.-C. Emma; S. W. Renee; A. F. Patricia; L. B. Anne; W. M. David; C. Teresa	2020	Developing an Animal Welfare Assessment Protocol for Livestock Transported by Sea	Animals, Vol 10, Iss 705, p	
40.	L. N. Edwards-Callaway; M. S. Calvo-Lorenzo	2020	Animal welfare in the U.S. slaughter industry-a focus on fed cattle	Journal of animal science	
41.	H. Cuthbertson; G. Tarr; K. Loudon; S. Lomax; P. White; P. McGreevy; R. Polkinghorne; L. A. González	2020	Using infrared thermography on farm of origin to predict meat quality and physiological response in cattle (Bos Taurus) exposed to transport and marketing	Meat science	
42.	M. Costa	2020	Comprehensive evaluation and implementation of improvement actions in bovine abattoirs to reduce pathogens exposure	Preventive veterinary medicine. 2020 Mar., v. 176	
43.	T. Collins; C. Stockman; J. O. Hampton; A. Barnes	2020	Identifying animal welfare impacts of livestock air transport	Aust Vet J	10.1111/avj.12927
44.	S. L. Collins; J. Kull; C. Benham; P. Krawczel; K. D. Donohue; M. Caldwell	2020	Comparison of penetrating and non-penetrating captive bolt in an alternative occipital approach in calves	Animal Welfare	10.7120/09627286.29.1.059

45.	Q. M. Chen; K. X. Qu; Z. J. Ma; J. X. Zhan; F. W. Zhang; J. F. Shen; Q. Q. Ning; P. Jia; J. C. Zhang; N. B. Chen; H. Chen; B. Z. Huang; C. Z. Lei	2020	Genome-Wide Association Study Identifies Genomic Loci Associated With Neurotransmitter Concentration in Cattle	Frontiers in Genetics	10.3389/fgene.2020.00139
46.	J. A. Cervantes-Cazares; C. Perez-Linares; F. Figueroa-Saavedra; A. R. Tamayo-Sosa; A. Barreras-Serrano; F. G. Rios-Rincon; E. Sanchez-Lopez; I. C. Garcia-Reynoso; P. M. Peraza; A. L. Villanueva; L. A. Garcia-Vega	2020	Comparison of surgical castration at birth versus immunocastration on carcass and meat traits in growing Holstein males	Revista Mexicana De Ciencias Pecuarias	10.22319/rmcp.v11i2.4885
47.	A. A. Carrasco-García; V. T. Pardío-Sedas; G. G. León-Banda; C. Ahuja-Aguirre; P. Paredes-Ramos; B. C. Hernández-Cruz; V. Vega Murillo	2020	Effect of stress during slaughter on carcass characteristics and meat quality in tropical beef cattle	Asian-Australasian journal of animal sciences	
48.	S. Buczinski; J. Dubuc; V. Bourgeois; P. Baillargeon; N. Côté; G. Fecteau	2020	Validation of serum gamma-glutamyl transferase activity and body weight information for identifying dairy calves that are too young to be transported to auction markets in Canada	J Dairy Sci	10.3168/jds.2019-17601
49.	G. Bozzo; E. Bonerba; R. Barrasso; R. Roma; F. Luposella; N. Zizzo; G. Tantillo	2020	Evaluation of the Occurrence of False Aneurysms During Halal Slaughtering and Consequences on the Animal's State of Consciousness	Animals (Basel)	10.3390/ani10071183
50.	J. Bokma; R. Boone; P. Deprez; B. Pardon	2020	Short communication: Herd-level analysis of antimicrobial use and mortality in veal calves: Do herds with low usage face higher mortality?	Journal of Dairy Science	10.3168/jds.2019-16764
51.	R. B. Barrasso, Elisabetta; Ceci, Edmondo; Roma, Rocco; Alò, Antonio; Mottola, Anna; Marchetti, Patrizia; Celano, Gaetano Vitale; Bozzo, Giancarlo	2020	Evaluation of the animal welfare during religious slaughtering	Italian journal of food safety	
52.	A. Alavi; F. Darki; M. M. R. Bidgoli; D. Zare-Abdollahi; A. Moini; M. M. Shahshahani; J. Fischer; E. Elahi	2020	Mutation in ALOX12B likely cause of POI and also ichthyosis in a large Iranian pedigree	Mol Genet Genomics	10.1007/s00438-020-01663-z

53.	M. R. Alam; M. J. Islam; A. Amin; A. H. Shaikat; M. R. Pasha; R. E. Doyle	2020	Animal-Based Welfare Assessment of Cattle and Water Buffalo in Bangladeshi Slaughterhouses	J Appl Anim Welf Sci	10.1080/10888705.2019.1620608
54.	I. Zulkifli; Z. Wakiman; A. Q. Sazili; Y. M. Goh; A. Jalila; Z. Zunita; E. A. Awad	2019	Effect of shackling, electrical stunning and halal slaughtering method on stress-linked hormones in broilers	South African Journal of Animal Science	10.4314/sajas.v49i3.20
55.	I. Zulkifli; A. A. Abubakar; A. Q. Sazili; Y. M. Goh; J. C. Imlan; U. Kaka; A. B. Sabow; E. A. Awad; A. H. Othman; R. Raghazali; C. J. C. Phillips; H. N. Quaza Nizamuddin; H. Mitin	2019	The Effects of Sea and Road Transport on Physiological and Electroencephalographic Responses in Brahman Crossbred Heifers	Animals (Basel)	10.3390/ani9050199
56.	J. Zhou; L. Liu; C. J. Chen; M. Zhang; X. Lu; Z. Zhang; X. Huang; Y. Shi	2019	Genome-wide association study of milk and reproductive traits in dual-purpose Xinjiang Brown cattle	BMC genomics	
57.	E. Zhou; I. Conejeros; U. Gärtner; S. Mazurek; C. Hermosilla; A. Taubert	2019	Metabolic requirements of Besnoitia besnoiti tachyzoite-triggered NETosis	Parasitology research	
58.	A. F. Zakki; A. Windyandari; Suharto; A. Ramadhan	2019	COMPARATIVE STUDY ON CATAMARAN AND MONOHULL FOR THE HULL FORM DESIGN OF LIVESTOCK CARRIER	Journal of Engineering Science and Technology	
59.	S. A. Woolley; K. L. M. Eager; I. M. Häfliger; A. Bauer; C. Drögemüller; T. Leeb; B. A. O'Rourke; I. Tammen	2019	An ABCA12 missense variant in a Shorthorn calf with ichthyosis fetalis	Animal genetics	
60.	I. Wolfram; G. Forstenpointner; F. J. M. Smulders; P. Paulsen	2019	The development of meat demand and subsequent changes in the transport of slaughter animals in Austria in the period 1850-1910	Wiener Tierarztliche Monatsschrift	
61.	R. D. Warner	2019	Review: Analysis of the process and drivers for cellular meat production	Animal	10.1017/s1751731119001897
62.	D. R. Wagner; H. C. Kline; M. S. Martin; L. R. Alexander; T. Grandin; L. N. Edwards-Callaway	2019	The effects of bolt length on penetration hole characteristics, brain damage and specified-risk material dispersal in finished cattle stunned with a penetrating captive bolt stunner	Meat science	
63.	P. Vale	2019	The Expansion of Intensive Beef Farming to the Brazilian Amazon	Global environmental change. 2019 July, v. 57	
64.	J. Tunstall; K. Mueller; D. G. White; J. W. H. Oultram; H. M. Higgins	2019	Lameness in Beef Cattle: UK Farmers' Perceptions, Knowledge, Barriers, and Approaches to Treatment and Control	Frontiers in Veterinary Science	10.3389/fvets.2019.00094

65.	K. Tighe; N. Piggott; O. Cacho; S. Mounter; R. Villano	2019	Does Consumer Interest in the Live Export Trade Affect Australian Meat Demand?	Australasian Agribusiness Review	
66.	H. Tergast	2019	Tierwohl und Rentabilität verbinden<br><br>Thünen-Institut schätzt Folgen eines Verbots der ganzjährigen Anbindehaltung		
67.	A. Small; J. Lea; D. Niemeyer; J. Hughes; D. McLean; J. McLean; J. Ralph	2019	Development of a microwave stunning system for cattle 2: Preliminary observations on behavioural responses and EEG	Res Vet Sci	10.1016/j.rvsc.2018.11.010
68.	A. Sharma; U. Kennedy; C. Schuetze; C. J. C. Phillips	2019	The Welfare of Cows in Indian Shelters	Animals	10.3390/ani9040172
69.	P. Schnyder; L. Schönecker; G. Schüpbach-Regula; M. Meylan	2019	[Transport of veal calves from birth farms to veal farms and calf management in Swiss dairy farms]	Schweiz Arch Tierheilkd	10.17236/sat00214
70.	M. Sánchez-Hidalgo	2019	Associations between Pre-Slaughter and Post-Slaughter Indicators of Animal Welfare in Cull Cows	Animals. 2019 Sept. 02, v. 9, no. 9	
71.	S. Ryu; R. J. Soares Magalhães; B. C. Chun	2019	The impact of expanded brucellosis surveillance in beef cattle on human brucellosis in Korea: an interrupted time-series analysis	BMC Infect Dis	10.1186/s12879-019-3825-6
72.	N. H. Rutherford; F. O. Lively; G. Arnott	2019	Evaluation of rumen temperature as a novel indicator of meat quality: Rumen temperature and haematological indicators of stress during the pre-slaughter period as predictors of instrumental meat quality in bulls	Meat science	
73.	L. S. Rocha; D. M. Silva; M. P. Silva; P. M. P. Vidigal; J. C. F. Silva; S. T. Guerra; M. G. Ribeiro; T. A. d. O. Mendes; A. d. O. B. Ribon	2019	Comparative genomics of Staphylococcus aureus associated with subclinical and clinical bovine mastitis	PloS one	
74.	A. M. Reiche; J. L. Oberson; P. Silacci; J. Messadène-Chelali; H. D. Hess; F. Dohme-Meier; P. A. Dufey; E. M. C. Terlouw	2019	Pre-slaughter stress and horn status influence physiology and meat quality of young bulls	Meat science	
75.	C. Reiber; P. Baisch; M. Chagunda; A. Valle Zárate	2019	Ökologische und konventionelle Milchviehhaltung im Vergleich<br><br>Haltungsbedingungen, Tierwohlindikatoren und Leistung von Fleckvieh und Holstein Kühen		

76.	J. Regenstine; M. N. Riaz; M. M. Chaudry	2019	The Religious Slaughter of Animals A US Perspective on Regulations and Animal Welfare Guidelines	Handbook of Halal Food Production	
77.	I. C. Pritsch; E. C. A. Stanula; A. dos Anjos; J. A. Bertot; M. B. Molento	2019	Fascioliasis in buffaloes: A 5-year forecast analysis of the disease based on a 15-year survey in Brazil	Revista Brasileira De Parasitologia Veterinaria	10.1590/s1984-29612019040
78.	H. A. O'Neill	2019	A review on the involvement of catecholamines in animal behaviour	South African Journal of Animal Science	10.4314/sajas.v49i1.1
79.	A. H. O'Connor; E. A. M. Bokkers; I. J. M. de Boer; H. Hogeveen; R. Sayers; N. Byrne; E. Ruelle; L. Shalloo	2019	Associating cow characteristics with mobility scores in pasture-based dairy cows	J Dairy Sci	10.3168/jds.2018-15719
80.	S. Ninomiya	2019	Grooming Device Effects on Behaviour and Welfare of Japanese Black Fattening Cattle	Animals. 2019 Apr. 23, v. 9, no. 4	
81.	O. Nina Dam; T. Nils; T. Peter Thorup; H. Hans	2019	Evaluation of the performance of register data as indicators for dairy herds with high lameness prevalence	Acta Veterinaria Scandinavica, Vol 61, Iss 1, Pp 1-	
82.	S. S. Nielsen; P. Sandøe; S. U. Kjølsted; J. S. Agerholm	2019	Slaughter of Pregnant Cattle in Denmark: Prevalence, Gestational Age, and Reasons	Animals: an open access journal from MDPI	
83.	S. S. Nielsen; M. A. Krogh; S. L. Munch; N. Capion	2019	Effect of non-perforating abomasal lesions on reproductive performance, milk yield and carcass weight at slaughter in Danish Holstein cows	Prev Vet Med	10.1016/j.prevetmed.2019.04.001
84.	K. Muri; S. M. Stubsjoen; G. Vasdal; R. O. Moe; E. G. Granquist	2019	Associations between qualitative behaviour assessments and measures of leg health, fear and mortality in Norwegian broiler chicken flocks	Applied Animal Behaviour Science	10.1016/j.applanim.2018.12.010
85.	S. L. Munch	2019	Prevalence of abomasal lesions in Danish Holstein cows at the time of slaughter	American Dairy Science Association Journal of dairy science. 2019 June, v. 102, no. 6	
86.	K. Mõtus; T. Niine; A. Viltrop; U. Emanuelson	2019	Herd-Level on-Farm Mortality in Extensively Managed Beef Herds	Journal of applied animal welfare science : JAAWS	
87.	T. F. Moreira; R. R. Nicolino; R. M. Meneses; G. V. Fonseca; L. M. Rodrigues; E. J. Facury Filho; A. U. Carvalho	2019	Risk factors associated with lameness and hoof lesions in pasture-based dairy cattle systems in southeast Brazil	J Dairy Sci	10.3168/jds.2018-16215
88.	S. Millward; K. Mueller; R. Smith; H. M. Higgins	2019	A Post-mortem Survey of Bovine Female Reproductive Tracts in the UK	Front Vet Sci	10.3389/fvets.2019.00451

89.	F. S. Mendonça; R. Z. Vaz; F. N. Vaz; W. S. Leal; I. D. B. Silveira; J. Restle; A. A. Boligon; F. F. Cardoso	2019	Causes of bruising in carcasses of beef cattle during farm, transport, and slaughterhouse handling in Brazil	Anim Sci J	10.1111/asj.13151
90.	D. M. Melendez; S. Marti; D. Gellatly; W. Nordi; D. Haley; T. Schwinghamer; K. S. Schwartzkopf-Genswein	2019	Effects of transport time and rest stop duration on welfare indicators of beef cattle travelling by road	Journal of Animal Science	
91.	E. Meili	2019	Hof- und Weideschlachtung - Alternative zu Tiertransport		
92.	E. McLoughlin	2019	Knowing cows: Transformative mobilizations of human and non-human bodies in an emotionography of the slaughterhouse	Gender Work and Organization	10.1111/gwao.12247
93.	K. May; K. Brügemann; C. Strube; S. König	2019	Strategien der Parasitenbekämpfung beim Milchvieh in Weideproduktionssystemen<br><br>Was kann die Zucht leisten?		
94.	A. Mato; R. Rodríguez-Vázquez; M. López-Pedrouso; S. Bravo; D. Franco; C. Zapata	2019	The first evidence of global meat phosphoproteome changes in response to pre-slaughter stress	BMC genomics	
95.	C. Masmeijer; B. Devriendt; T. Rogge; K. van Leenen; L. De Cremer; B. Van Ranst; P. Deprez; E. Cox; B. Pardon	2019	Randomized field trial on the effects of body weight and short transport on stress and immune variables in 2- to 4-week-old dairy calves	J Vet Intern Med	10.1111/jvim.15482
96.	S. Mann; S. Beciu; G. A. Arghiroiu	2019	Farm animals against open borders: uncovering discrepancies between narratives and evidence regarding actors and motives in the animal trade	Ciencia Rural	10.1590/0103-8478cr20180567
97.	L. Magrin	2019	Health, behaviour and growth performance of Charolais and Limousin bulls fattened on different types of flooring	Animal. 2019 Nov., v. 13, no. 11	
98.	D. E. Lowe	2019	The effect of diet and covering fully slatted concrete floors with rubber strips on the intake, performance and cleanliness of dairy-origin bulls	Animal. 2019 Sept., v. 13, no. 9	
99.	K. M. W. Loudon; G. Tarr; D. W. Pethick; I. J. Lean; R. Polkinghorne; M. Mason; F. R. Dunshea; G. E. Gardner; P. McGilchrist	2019	The Use of Biochemical Measurements to Identify Pre-Slaughter Stress in Pasture Finished Beef Cattle	Animals	10.3390/ani9080503

100.	J. Loredo-Osti; E. Sánchez-López; A. Barreras-Serrano; F. Figueroa-Saavedra; C. Pérez-Linares; M. Ruiz-Albarrán; M. Domínguez-Muñoz	2019	An evaluation of environmental, intrinsic and pre- and post-slaughter risk factors associated to dark-cutting beef in a Federal Inspected Type slaughter plant	Meat Sci	10.1016/j.meatsci.2018.12.007
101.	J. Liu; L. Toma; A. P. Barnes; A. Stott	2019	Farmers' Uptake of Animal Health and Welfare Technological Innovations. Implications for Animal Health Policies	Frontiers in veterinary science	
102.	Z. Lin; C. He; D. R. Magstadt; V. L. Cooper; M. D. Kleinhenz; J. S. Smith; P. J. Gorden; L. W. Wulf; J. F. Coetzee	2019	Tissue residue depletion and estimation of extralabel meat withdrawal intervals for tulathromycin in calves after pneumatic dart administration	J Anim Sci	10.1093/jas/skz231
103.	N. E.-C. Lily; W. Jennifer; B. T. Cassandra	2019	Culling Decisions and Dairy Cattle Welfare During Transport to Slaughter in the United States	Frontiers in Veterinary Science, Vol	
104.	G. N. Levina	2019	Effects of Heifer Age and Liveweight at the Final Stage of Puberty and the First Service Conception on Cow Productivity and Welfare	Russian agricultural sciences. 2019 Mar., v. 45, no. 2	
105.	F. Leiber; A. Müller; V. Maurer; C. Schader; A. Bieber	2019	Organic dairy farming<br><br>towards sustainability		
106.	A. Lamey	2019	The Animal Ethics of Temple Grandin: A Protectionist Analysis	Journal of agricultural & environmental ethics. 2019 Feb., v. 32, no. 1	
107.	K. E. Koralesky; D. Fraser	2019	Perceptions of on-farm emergency slaughter for dairy cows in British Columbia	J Dairy Sci	10.3168/jds.2018-14814
108.	M. Knock; G. A. Carroll	2019	The Potential of Post-Mortem Carcass Assessments in Reflecting the Welfare of Beef and Dairy Cattle	Animals : an open access journal from MDPI	
109.	H. C. Kline; D. R. Wagner; L. N. Edwards-Callaway; L. R. Alexander; T. Grandin	2019	Effect of captive bolt gun length on brain trauma and post-stunning hind limb activity in finished cattle Bos taurus	Meat science	
110.	J. P. Kamenik, Vaclav; Pyszko, Martin; Voslarova, Eva	2019	Cattle stunning with a penetrative captive bolt device: A review	Animal science journal = Nihon chikusan Gakkaiho	
111.	H. Jumba	2019	Gender perspectives in economic assessment of East Coast Fever vaccination on household welfare among smallholder dairy cattle farmers in Uasin Gishu County, Kenya		

112.	M. Jorquera-Chavez; S. Fuentes; F. R. Dunshea; E. C. Jongman; R. D. Warner	2019	Computer vision and remote sensing to assess physiological responses of cattle to pre-slaughter stress, and its impact on beef quality: A review	Meat Sci	10.1016/j.meatsci.2019.05.007
113.	A. K. Howell; C. M. McCann; F. Wickstead; D. J. L. Williams	2019	Co-infection of cattle with Fasciola hepatica or F. gigantica and Mycobacterium bovis: A systematic review	PloS one	
114.	S. Hoischen-Taubner; L. Fingerhut; V. Uhlig; D. Möller; A. Sundrum	2019	Herausforderungen und Chancen eines innovativen Konzeptes zur Bewertung von Tierschutzleistungen		
115.	J. Hanley; A. Garcia-Ara; W. Wapenaar	2019	Cattle and sheep farmers' opinions on the provision and use of abattoir rejection data in the United Kingdom	The Veterinary record	
116.	A. Grist; J. A. Lines; R. Bock; T. G. Knowles; S. B. Wotton	2019	An Examination of the Performance of Blank Cartridges Used in Captive Bolt Devices for the Pre-Slaughter Stunning and Euthanasia of Animals	Animals	10.3390/ani9080552
117.	A. Grist	2019	Macroscopic Examination of Multiple-Shot Cattle Heads_An Animal Welfare Due Diligence Tool for Abattoirs Using Penetrating Captive Bolt Devices?	Animals. 2019 June 06, v. 9, no. 6	
118.	N. Grisel; B. Viviana; G. Carmen; J. C. P. Clive	2019	Physiological and Behavioural Responses of Cattle to High and Low Space, Feed and Water Allowances During Long Distance Transport in the South of Chile	Animals, Vol 9, Iss 5, p	
119.	B. Grimard; A. de Boyer des Roches; M. Coignard; A. Lehébel; A. Chuiton; L. Mounier; I. Veissier; R. Guatteo; N. Bareille	2019	Relationships between welfare and reproductive performance in French dairy herds	Veterinary journal (London, England : 1997)	
120.	T. J. Gibson; S. E. O. Oliveira; F. A. D. Costa; N. G. Gregory	2019	Electroencephalographic assessment of pneumatically powered penetrating and non-penetrating captive-bolt stunning of bulls	Meat science	
121.	J. A. B. Garcia; R. Z. Vaz; F. N. Vaz; J. Restle; F. S. Mendonca	2019	Pre-slaughter factors associated with severe bruising in different primary commercial cuts of bovine carcasses	Revista Ciencia Agronomica	10.5935/1806-6690.20190080
122.	A. Fuseini	2019	The brain, unconsciousness and death: a critical appraisal with regard to halal meat production	Animal Welfare	10.7120/09627286.28.2.165
123.	A. G. Fouad Ali Abdullah, Borilova; Iva, Steinhauserova	2019	Halal Criteria Versus Conventional Slaughter Technology	Animals, Vol 9, Iss 8, p	
124.	G. De Rosa	2019	Different assessment systems fail to agree on the evaluation of dairy cattle welfare at farm level	Livestock science. 2019 Nov., v. 229	

125.	V. A. de Lima; M. C. Ceballos; N. G. Gregory; M. Da Costa	2019	Effect of different catching practices during manual upright handling on broiler welfare and behavior	Poultry Science	10.3382/ps/pez284
126.	K. Das	2019	Effects of new technology on the current manufacturing process of yogurt-to increase the overall marketability of yogurt	Lebensmittel-Wissenschaft + [i.e. und] Technologie. 2019 July, v. 108	
127.	R. J. Cox; P. Nol; C. K. Ellis; M. V. Palmer	2019	Research with Agricultural Animals and Wildlife	ILAR journal	
128.	J. E. Coombe	2019	Antimicrobial stewardship in the dairy industry: responding to the threat of antimicrobial resistance	Australian veterinary journal. 2019 July, v. 97, no. 7	
129.	M. S. Cockram	2019	Fitness of animals for transport to slaughter	Canadian Veterinary Journal-Revue Veterinaire Canadienne	
130.	F. Cirone; B. Padalino; D. Tullio; P. Capozza; M. Lo Surdo; G. Lanave; A. Pratelli	2019	Prevalence of Pathogens Related to Bovine Respiratory Disease Before and After Transportation in Beef Steers: Preliminary Results	Animals (Basel)	10.3390/ani9121093
131.	K. Cimer; S. March; J. Brinkmann; S. Fetscher; D. Gieseke; L. Schrader; A. Schubbert; U. Schultheiß; R. Zapf; U. Knierim	2019	Tierschutzindikatoren für die betriebliche Eigenkontrolle _ Impulse für die Ökologische Landwirtschaft		
132.	C. D. S. Carvalho; M. D. da Costa; M. C. M. Goncalves; T. A. Diniz; G. C. da Silva; H. C. Ferreira; K. C. B. Pereira; A. L. D. Castro; L. V. Santos	2019	Animal and workers' welfare during agricultural fair	Journal of Animal Behaviour and Biometeorology	10.31893/2318-1265jabb.v7n2p78-85
133.	L. V. Burn; A. T. Ramos; A. P. M. Veiga; S. E. Moron; F. M. Cordova; F. R. C. Miotto; E. B. Viana; F. C. Zimermann; S. Minharro; N. R. Stefanine; L. F. Sousa	2019	Evaluation of muscle tissue and liver glycogen of cattle submitted to transport over long distances and subjected to emergency slaughter	Arquivo Brasileiro De Medicina Veterinaria E Zootecnia	10.1590/1678-4162-10233
134.	A. Budzik; T. Budzik	2019	Ethical Aspects of Sustainable Development in Polish Enterprises Transporting Animals for Slaughter	European Journal of Sustainable Development	10.14207/ejsd.2019.v8n3p347
135.	J. Brinkmann; K. Cimer; S. March; S. Ivermeyer; A.	2019	Praxistaugliche Tierschutzindikatoren für die betriebliche Eigenkontrolle - ein Vorschlag für die deutsche Milch- und Mastrinderhaltung		

	Pelzer; U. Schultheiß; R. Zapf; C. Winckler				
136.	V. M. Bravo; T. G. Knowles; C. Gallo	2019	Factors Affecting the Welfare of Calves in Auction Markets	Animals	10.3390/ani9060333
137.	U. Braun; A. Wiest; T. Lutz; B. Riond; M. Stirn; M. Hilbe; M. R. Baumgartner; T. M. Binz	2019	Hair cortisol concentration in veal calves reared under two different welfare production labels	Research in veterinary science	
138.	K. Borzuta; D. Lisiak; P. Janiszewski; E. Grzeskowiak	2019	THE PHYSIOLOGICAL ASPECTS, TECHNIQUE AND MONITORING OF SLAUGHTER PROCEDURES AND THEIR EFFECTS ON MEAT QUALITY - A REVIEW	Annals of Animal Science	10.2478/aoas-2019-0039
139.	J. A. Bethancourt-Garcia; R. Z. Vaz; F. N. Vaz; W. B. Silva; L. L. Pascoal; F. S. Mendonca; C. C. da Vara; A. J. C. Nunez; J. Restle	2019	Pre-slaughter factors affecting the incidence of severe bruising in cattle carcasses	Livestock Science	10.1016/j.livsci.2019.02.009
140.	A. A. Barragan; J. Lakritz; M. K. Carman; S. Bas; E. Hovingh; G. M. Schuenemann	2019	Short communication: Assessment of biomarkers of inflammation in the vaginal discharge of postpartum dairy cows diagnosed with clinical metritis	Journal of dairy science	
141.	F. Baier; T. Grandin; T. Engle; L. Edwards-Callaway	2019	Evaluation of Hair Characteristics and Animal Age on the Impact of Hair Cortisol Concentration in Feedlot Steers	Frontiers in Veterinary Science	10.3389/fvets.2019.00323
142.	J. W. Aleri; B. C. Hine; M. F. Pyman; P. D. Mansell; W. J. Wales; B. Mallard; M. A. Stevenson; A. D. Fisher	2019	Associations between immune competence, stress responsiveness, and production in Holstein-Friesian and Holstein-Friesian × Jersey heifers reared in a pasture-based production system in Australia	J Dairy Sci	10.3168/jds.2018-14578
143.	N. I. Agbeboh; J. L. OlaJide; I. O. Oladele; S. O. Babarinsa	2019	Kinetics of moisture sorption and improved tribological performance of keratinous fiber-reinforced ortho-phthalic polyester biocomposites	Journal of Natural Fibers	10.1080/15440478.2018.1434849
144.	V. K. Ya; G. S. Yu; L. Y. Bozhyk	2018	Cross-border dissemination of lumpy skin disease risc analysis for Ukraine		
145.	L. Xiaofei; Z. Sarah; S. Michelle; J. C. P. Clive	2018	Perception of animal welfare issues during Chinese transport and slaughter of livestock by a sample of stakeholders in the industry	PLoS ONE, Vol 13, Iss 6, p e	

146.	E. E. B. Wigham, Andy; Wotton, Steve	2018	Assessing cattle welfare at slaughter - Why is it important and what challenges are faced?	Meat science	
147.	J. Wambui; P. Lamuka; E. Karuri; J. Matofari	2018	Animal Welfare Knowledge, Attitudes, and Practices of Stockpersons in Kenya	Anthrozoos	10.1080/08927936.2018.1482111
148.	B. J. Walmsley	2018	A review of factors influencing key biological components of maternal productivity in temperate beef cattle	Animal production science. 2018, v. 58, no. 1	
149.	K. von Holleben; Y. Schneider; M. von Wenzlawowicz	2018	Study on the eligibility of a pneumatic captive bolt stunner for cattle stunning under routine conditions (80/h)	Fleischwirtschaft	
150.	B. Viviana; G. Carmen; A.-J. Gerardo	2018	Effects of Short Transport and Prolonged Fasting in Beef Calves	Animals, Vol 8, Iss 10, p	
151.	S. J. Van Schyndel; C. A. Bauman; O. B. Pascottini; D. L. Renaud; J. Dubuc; D. F. Kelton	2018	Reproductive management practices on dairy farms: The Canadian National Dairy Study 2015	Journal of dairy science	
152.	M. M. J. van Riet; E. J. Bos; B. Ampe; P. Bikker; D. Vanhauteghem; F. Van Bockstaele; P. Cornillie; W. Van Den Broeck; G. Du Laing; D. Maes; F. A. M. Tuyttens; G. P. J. Janssens; S. Millet	2018	Long-term impact of zinc supplementation in sows: Impact on claw quality	Journal of Swine Health and Production	
153.	M. Valadez-Noriega; L. X. Estévez-Moreno; A. A. Rayas-Amor; M. S. Rubio-Lozano; F. Galindo; G. C. Miranda-de la Lama	2018	Livestock hauliers' attitudes, knowledge and current practices towards animal welfare, occupational wellbeing and transport risk factors: A Mexican survey	Preventive veterinary medicine	
154.	K. Uttara; S. Arvind; J. C. P. Clive	2018	The Sheltering of Unwanted Cattle, Experiences in India and Implications for Cattle Industries Elsewhere	Animals, Vol 8, Iss 5, p	
155.	C. M. Tramontini; R. M. Cardozo; J. D. Arieira	2018	Identification of productive systems of beef cattle in the northwest Region of Parana, Brazil	Semina-Ciencias Agrarias	10.5433/1679-0359.2018v39n3p1319
156.	G. Temple	2018	Welfare Problems in Cattle, Pigs, and Sheep that Persist Even Though Scientific Research Clearly Shows How to Prevent Them	Animals, Vol 8, Iss 7, p	

157.	J. Stojkov; G. Bowers; M. Draper; T. Duffield; P. Duivenvoorden; M. Groleau; D. Haupstein; R. Peters; J. Pritchard; C. Radom; N. Sillett; W. Skippon; H. Trepanier; D. Fraser	2018	Management of cull dairy cows-Consensus of an expert consultation in Canada	Journal of Dairy Science	10.3168/jds.2018-14919
158.	J. Stojkov; G. Bowers; M. Draper; T. Duffield; P. Duivenvoorden; M. Groleau; D. Haupstein; R. Peters; J. Pritchard; C. Radom; N. Sillett; W. Skippon; H. Trépanier; D. Fraser	2018	Hot topic: Management of cull dairy cows-Consensus of an expert consultation in Canada	Journal of dairy science	
159.	H. Stella Maris; E. A. M. K. Rick; J. C. M. v. E. Frank	2018	Relationship between Methods of Loading and Unloading, Carcass Bruising, and Animal Welfare in the Transportation of Extensively Reared Beef Cattle	Animals, Vol 8, Iss 7, p	
160.	S. Stalder; X. Sidler; M. Hassig	2018	Milk production without use of antibiotics	Schweizer Archiv Fur Tierheilkunde	10.17236/sat00188
161.	S. Stalder; X. Sidler; M. Hässig	2018	Deskriptive Studie zur antibiotikafreien Milchproduktion beim Rind	Schweizer Archiv fur Tierheilkunde	
162.	J. K. Shearer	2018	Euthanasia of Cattle: Practical Considerations and Application	Animals	10.3390/ani8040057
163.	A. Segura; M. Bertoni; P. Auffret; C. Klopp; O. Bouchez; C. Genthon; A. Durand; Y. Bertin; E. Forano	2018	Transcriptomic analysis reveals specific metabolic pathways of enterohemorrhagic Escherichia coli O157:H7 in bovine digestive contents	BMC Genomics	10.1186/s12864-018-5167-y
164.	R. Schmitz	2018	Changes of performance, energy efficiency, ruminal fermentation and animal health depending on energy concentration in roughage and amounts of concentrates in dairy cows during early lactation ; Beeinflussung der Leistung, der Energieverwertung, der Pansenfermentation sowie der Tiergesundheit durch die Energiekonzentration im Grobfutter sowie die Kraftfuttermenge bei Milchkühen während der Frühlaktation		
165.	A. A. Sardar; P. Saha; M. Chatterjee; D. K. Bera; P. Biswas; D. Maji; S. K. Guha; N. Basu; A. K. Maji	2018	Insecticide susceptibility status of Phlebotomus argentipes and polymorphisms in voltage-gated sodium channel (vgsc) gene in Kala-azar endemic areas of West Bengal, India	Acta Trop	10.1016/j.actatropica.2018.06.005

166.	D. Salilew-Wondim; M. Saeed-Zidane; M. Hoelker; S. Gebremedhn; M. Poirier; H. O. Pandey; E. Tholen; C. Neuhoff; E. Held; U. Besenfelder; V. Havlicek; F. Rings; E. Fournier; D. Gagné; M. A. Sirard; C. Robert; A. Gad; K. Schellander; D. Tesfaye	2018	Genome-wide DNA methylation patterns of bovine blastocysts derived from in vivo embryos subjected to in vitro culture before, during or after embryonic genome activation	BMC Genomics	10.1186/s12864-018-4826-3
167.	C. Saegerman; S. Bertagnoli; G. Meyer; J. P. Ganière; P. Caufour; K. De Clercq; P. Jacquiet; G. Fournié; C. Hautefeuille; F. Etore; J. Casal	2018	Risk of introduction of lumpy skin disease in France by the import of vectors in animal trucks	PLoS One	10.1371/journal.pone.0198506
168.	A. B. M. Rubayet Bostami; H. S. Mun; C. J. Yang	2018	Loin eye muscle physico-chemical attributes, sensory evaluation and proximate composition in Korean Hanwoo cattle subjected to slaughtering along with stunning with or without pithing	Meat Sci	10.1016/j.meatsci.2018.06.032
169.	K. Reimus; T. Orro; U. Emanuelson; A. Viltrop; K. Mõtus	2018	On-farm mortality and related risk factors in Estonian dairy cows	Preventive veterinary medicine	
170.	M. C. Queiroga	2018	Local and systemic humoral response to ovine mastitis caused by Staphylococcus epidermidis	Sage Open Medicine	10.1177/2050312118801466
171.	L. Pederson; J. Yates; A. Wieman	2018	Preparation and Response to Truck Accidents on Highways Involving Cattle	The Veterinary clinics of North America. Food animal practice	
172.	M. Paolo; G. H. Ian; R. Gustaf; M. H. Saidou; N. Victor Ngu; N. T. Vincent; L. M. Kenton; M. d. B. Barend; P. Thibaud	2018	Drivers of Live Cattle Price in the Livestock Trading System of Central Cameroon	Frontiers in Veterinary Science, Vol	
173.	J. H. Pagella; R. W. Mayes; F. J. Perez-Barberia; E. R. Orskov	2018	The development of an intraruminal nylon bag technique using non-fistulated animals to assess the rumen degradability of dietary plant materials	Animal	10.1017/s1751731117001203
174.	B. Padalino; D. Tullio; S. Cannone; G. Bozzo	2018	Road Transport of Farm Animals: Mortality, Morbidity, Species and Country of Origin at a Southern Italian Control Post	Animals : an open access journal from MDPI	

175.	S. E. O. Oliveira; N. G. Gregory; F. A. Dalla Costa; T. J. Gibson; O. A. Dalla Costa; M. J. R. Paranhos da Costa	2018	Effectiveness of pneumatically powered penetrating and non-penetrating captive bolts in stunning cattle	Meat science	
176.	S. E. O. Oliveira; F. A. Dalla Costa; T. J. Gibson; O. A. D. Costa; A. Coldebella; N. G. Gregory	2018	Evaluation of brain damage resulting from penetrating and non-penetrating stunning in Nelore Cattle using pneumatically powered captive bolt guns	Meat science	
177.	C. L. Olds; K. L. Mason; G. A. Scoles	2018	Rhipicephalus appendiculatus ticks transmit Theileria parva from persistently infected cattle in the absence of detectable parasitemia: implications for East Coast fever epidemiology	Parasit Vectors	10.1186/s13071-018-2727-6
178.	Y. Narayanan	2018	Cow Protectionism and Bovine Frozen-Semen Farms in India Analyzing Cruelty, Speciesism, and Climate Change	Society & Animals	10.1163/15685306-12341481
179.	A. K. G. Moorman; T. F. Duffield; M. A. Godkin; D. F. Kelton; J. Rau; D. B. Haley	2018	Associations between the general condition of culled dairy cows and selling price at Ontario auction markets	Journal of dairy science	
180.	N. S. Minka; J. O. Ayo	2018	Effects of different road conditions on rectal temperature, behaviour and traumatic injuries during transportation of different crosses of temperate/tropical breeds of heifers	Animal Production Science	10.1071/an16400
181.	F. Mikael; J. Annie; S. Stefan; F. Patrik; R. Mikael; W. Uno	2018	Route optimization as an instrument to improve animal welfare and economics in pre-slaughter logistics	PLoS ONE, Vol 13, Iss 3, p e	
182.	F. S. Mendonca; R. Z. Vaz; F. F. Cardoso; J. Restle; F. N. Vaz; L. L. Pascoal; F. A. Reimann; A. A. Boligon	2018	Pre-slaughtering factors related to bruises on cattle carcasses	Animal Production Science	10.1071/an16177
183.	E. Meili	2018	Mit Kuhmast die Fleischimporte halbieren		
184.	C. Meichtry; U. Glauser; M. Glardon; S. G. Ross; I. Lechner; B. P. Kneubuehl; D. Gascho; C. Spadavecchia; A. von Rotz; A. Stojiljkovic; M. H. Stoffel	2018	Assessment of a specifically developed bullet casing gun for the stunning of water buffaloes	Meat science	
185.	J. G. Maples; J. L. Lusk; D. S. Peel	2018	Unintended consequences of the quest for increased efficiency in beef cattle: When bigger isn't better	Food Policy	10.1016/j.foodpol.2017.11.005

186.	L. Magrin; M. Brscic; L. Armato; B. Contiero; G. Cozzi; F. Gottardo	2018	An overview of claw disorders at slaughter in finishing beef cattle reared in intensive indoor systems through a cross-sectional study	Preventive veterinary medicine	
187.	T. L. Lee; C. D. Reinhardt; S. J. Bartle; E. F. Schwandt; M. S. Calvo-Lorenzo; C. Vahl; J. A. Hagenmaier; M. J. Ritter; G. J. Vogel; D. U. Thomson	2018	An epidemiological investigation to determine the prevalence and clinical manifestations of slow-moving finished cattle presented to slaughter facilities	Translational Animal Science	10.1093/tas/txy056
188.	B. Lecorps; S. Kappel; D. M. Weary; M. A. G. von Keyserlingk	2018	Dairy calves' personality traits predict social proximity and response to an emotional challenge	Sci Rep	10.1038/s41598-018-34281-2
189.	C. H. Krog; J. S. Agerholm; S. S. Nielsen	2018	Fetal age assessment for Holstein cattle	PLoS One	10.1371/journal.pone.0207682
190.	K. E. Koralesky; D. Fraser	2018	Use of on-farm emergency slaughter for dairy cows in British Columbia	J Dairy Sci	10.3168/jds.2017-14320
191.	R. Khan; H. F. Guo; S. H. A. Raza; A. Rahman; M. Ayaz; L. S. Zan	2018	Halal slaughtering, welfare, and empathy in farm animals: a review	Tropical Animal Health and Production	10.1007/s11250-018-1644-1
192.	M. P. Keane; M. McGee; E. G. O'Riordan; A. K. Kelly; B. Earley	2018	Performance and welfare of steers housed on concrete slatted floors at fixed and dynamic (allometric based) space allowances	Journal of animal science	
193.	N. S. A. Jalil; A. V. Tawde; S. Zito; M. Sinclair; C. Fryer; Z. Idrus; C. J. C. Phillips	2018	Attitudes of the public towards halal food and associated animal welfare issues in two countries with predominantly Muslim and non-Muslim populations	Plos One	10.1371/journal.pone.0204094
194.	M. F. Iulietto; P. Sechi; C. M. Gaudenzi; L. Grispoldi; M. Ceccarelli; S. Barbera; B. T. Cenci-Goga	2018	Noise assessment in slaughterhouses by means of a smartphone app	Italian Journal of Food Safety	10.4081/ijfs.2018.7053
195.	S. Hüttel; R. Bürger; M. Stark; O. Kaufmann; N. Irrgang; D. Seifert; J. Zeitz; S. Ittner; S. Drexler	2018	Ökonomische, ökologische und Tierwohlaspekte der Weidehaltung von Hochleistungskühen		
196.	E. Humer; J. R. Aschenbach; V. Neubauer; I. Kroger; R. Khiaosa-ard; W. Baumgartner; Q. Zebeli	2018	Signals for identifying cows at risk of subacute ruminal acidosis in dairy veterinary practice	Journal of Animal Physiology and Animal Nutrition	10.1111/jpn.12850

197.	B. Hilana dos Santos Sena; D. Bruno Stéfano Lima; A. Aline Melgaço Bezerra de; A. Aline Zorzan de; C. Rafaella Jacinta de Bento; A. Adriano Braga Brasileiro de; M. Adriana Morato; B. João Paulo; L. Paula Rodrigues; G. Félix Hilário Diaz; M. Concepta; B. Donald; B. Francisco Ernesto Moreno	2018	Hemato-biochemical profile of meat cattle submitted to different types of pre-loading handling and transport times	International Journal of Veterinary Science and Medicine, Vol 6, Iss 1, Pp 90-	
198.	T. M. Hicks; S. O. Knowles; M. M. Farouk	2018	Global Provisioning of Red Meat for Flexitarian Diets	Frontiers in nutrition	
199.	T. Grandin	2018	Livestock-handling assessments to improve the welfare of cattle, pigs and sheep	Animal Production Science	10.1071/an16800
200.	I. F. Gorlov; A. V. Randelin; A. A. Mosolov; V. N. Hramova; V. A. Baranikov; B. K. Bolaev; I. V. Vladimtseva; A. B. Mulik; E. Y. Zlobina	2018	Glimalask-Vet" Feed Supplement For Reducing The Technological Stresses And Improving The Animal Welfare And Meat Quality In Beef Cattle Breeding"	Research Journal of Pharmaceutical Biological and Chemical Sciences	
201.	M. Glardon; B. K. Schwenk; F. Riva; A. von Holzena; S. G. Ross; B. P. Kneubuehl; M. H. Stoffel	2018	Energy loss and impact of various stunning devices used for the slaughtering of water buffaloes	Meat Science	10.1016/j.meatsci.2017.09.014
202.	A. Gin; T. Sato; A. Tohei; R. Miura; H. Mizutani; H. Amao; Y. Yamada; S. Kamiya; I. Yosimura; M. Mochizuki	2018	Study of stress in dairy cattle during student practical training on a farm	Japanese Journal of Veterinary Research	10.14943/jjvr.66.2.63
203.	J. N. Gilliam; J. Woods; J. Hill; J. K. Shearer; J. Reynolds; J. D. Taylor	2018	Evaluation of the CASH Dispatch Kit combined with alternative shot placement landmarks as a single-step euthanasia method for cattle of various ages	Animal Welfare	10.7120/09627286.27.3.225
204.	D. Gieseke	2018	Einfluss von Haltung und Management auf das Tierwohl in der Milchviehhaltung		
205.	G. Fordyce; H. McMillan; N. McGrath	2018	Postoperative healing and behaviour when surgical swabs are applied to calf dehorning wounds	Aust Vet J	10.1111/avj.12771
206.	C. Fischer-Tenhagen; M. Ladwig-Wiegard; W. Heuwieser; C. Thöne-Reineke	2018	Short communication: Is hair cortisol a potential indicator for stress caused by chronic lameness in dairy cows?	J Dairy Sci	10.3168/jds.2017-13967

207.	A. Ferlazzo	2018	The contribution of total and free iodothyronines to welfare maintenance and management stress coping in Ruminants and Equines: Physiological ranges and reference values	Elsevier Ltd Research in veterinary science. 2018 June, v. 118	
208.	E. Elahi; M. Abid; H. M. Zhang; W. J. Cui; S. Ul Hasson	2018	Domestic water buffaloes: Access to surface water, disease prevalence and associated economic losses	Preventive Veterinary Medicine	10.1016/j.prevetmed.2018.03.021
209.	J. S. Drouillard	2018	Current situation and future trends for beef production in the United States of America - A review	Asian-Australasian journal of animal sciences	
210.	R. G. de Queiroz; C. H. d. F. Domingues; M. E. A. Canozzi; R. G. Garcia; C. F. Ruviaro; J. O. J. Barcellos; J. A. R. Borges	2018	How do Brazilian citizens perceive animal welfare conditions in poultry, beef, and dairy supply chains?	PloS one	
211.	L. de la Cruz; T. J. Gibson; I. Guerrero-Legarreta; F. Napolitano; P. Mora-Medina; D. Mota-Rojas	2018	The welfare of water buffaloes during the slaughter process: A review	Livestock Science	10.1016/j.livsci.2018.03.014
212.	K. H. Dahl-Pedersen, M. S.; Houe, H.; Thomsen, P. T.	2018	Risk Factors for Deterioration of the Clinical Condition of Cull Dairy Cows During Transport to Slaughter	Front Vet Sci	10.3389/fvets.2018.00297
213.	K. Dahl-Pedersen; L. Foldager; M. S. Herskin; H. Houe; P. T. Thomsen	2018	Lameness scoring and assessment of fitness for transport in dairy cows: Agreement among and between farmers, veterinarians and livestock drivers	Research in veterinary science	
214.	T. Collins	2018	A Systematic Review of Heat Load in Australian Livestock Transported by Sea	Animals. 2018 Sept. 27, v. 8, no. 10	
215.	L. Carpentier	2018	Automatic cough detection for bovine respiratory disease in a calf house	IAgrE Biosystems engineering. 2018 Sept., v. 173	
216.	E. A. Buddle; H. J. Bray; R. A. Ankeny	2018	I Feel Sorry for Them": Australian Meat Consumers' Perceptions about Sheep and Beef Cattle Transportation"	Animals : an open access journal from MDPI	
217.	M. Brscic; M. K. Kirchner; U. Knierim; B. Contiero; F. Gottardo; C. Winckler; G. Cozzi	2018	Risk factors associated with beef cattle losses on intensive fattening farms in Austria, Germany and Italy	Veterinary Journal	10.1016/j.tvjl.2018.08.002
218.	G. B. Bozzo, Roberta; Marchetti, Patrizia; Roma, Rocco; Samoilis, Giorgio;	2018	Analysis of Stress Indicators for Evaluation of Animal Welfare and Meat Quality in Traditional and Jewish Slaughtering	Animals : an open access journal from MDPI	

	Tantillo, Giuseppina; Ceci, Edmondo				
219.	A. C. Borsanelli; D. F. Lappinb; L. Viora; D. Bennett; I. S. Dutra; B. W. Brandt; M. P. Riggio	2018	Microbiomes associated with bovine periodontitis and oral health	Veterinary Microbiology	10.1016/j.vetmic.2018.03.016
220.	S. E. Blum; R. J. Goldstone; J. P. R. Connolly; M. Répérant-Ferter; P. Germon; N. F. Inglis; O. Krifucks; S. Mathur; E. Manson; K. McLean; P. Rainard; A. J. Roe; G. Leitner; D. G. E. Smith	2018	Postgenomics Characterization of an Essential Genetic Determinant of Mammary Pathogenic Escherichia coli	mBio	10.1128/mBio.00423-18
221.	A. Bergschmidt; T. Lindena; S. Neuenfeldt; H. Tergast	2018	Folgenabschätzung eines Verbots der ganzjährigen Anbindehaltung von Milchkühen		
222.	A. Benzertiha; B. Kieronczyk; M. Rawski; A. Jozefiak; J. Mazurkiewicz; D. Jozefiak; M. S. Messikh; S. Swiatkiewicz	2018	Cultural and practical aspects of halal slaughtering in food production	Medycyna Weterynaryjna-Veterinary Medicine-Science and Practice	10.21521/mw.6023
223.	R. Armengol; L. Fraile	2018	Descriptive study for culling and mortality in five high-producing Spanish dairy cattle farms (2006-2016)	Acta veterinaria Scandinavica	
224.	M. Alam; M. Hasanuzzaman; M. M. Hassan; T. M. Rakib; M. E. Hossain; M. H. Rashid; M. A. Sayeed; L. B. Philips; M. A. Hoque	2018	Assessment of transport stress on cattle travelling a long distance (≈648 km), from Jessore (Indian border) to Chittagong, Bangladesh	Vet Rec Open	10.1136/vetreco-2017-000248
225.	M. A. Al-Mamun; R. L. Smith; A. Nigsch; Y. H. Schukken; Y. T. Gröhn	2018	A data-driven individual-based model of infectious disease in livestock operation: A validation study for paratuberculosis	PloS one	
226.	K. Adamczyk	2018	Dairy cattle welfare as a result of human-animal relationship _ a review	Annals of animal science. 2018 July 01, v. 18, no. 3	
227.		2018	Scottish CVO 'disappointed' with P&O decision	Vet Rec	10.1136/vr.k3903
228.	B. Zhou; G. Liu; Q. He; B. Li; X. Yu	2017	Dacin, one metalloproteinase from Deinagkistrodon acutus venom inhibiting contraction of mouse ileum muscle	BMC Biochem	10.1186/s12858-017-0086-0

229.	R. Zapf; U. Schultheiß; U. Knierim; J. Brinkmann; L. Schrader	2017	Tierwohl messen im Nutztierbestand - Leitfäden für die betriebliche Eigenkontrolle ; Assessing farm animal welfare _ guidelines for on-farm self-assessment		
230.	M. E. Youngers	2017	Case Study: Prevalence of horns and bruising in feedlot cattle at slaughter	Professional animal scientists. 2017 Feb., v. 33, no. 1	
231.	B. K. Wilson; C. J. Richards; D. L. Step; C. R. Krehbiel	2017	Best management practices for newly weaned calves for improved health and well-being	Journal of animal science	
232.	J. C. Thomas; J. R. Young; K. Schemann; P. Chankhamthong; S. Khounsy; S. Nampanya; P. A. Windsor; R. D. Bush	2017	Investigating baseline red meat slaughter operator capacity and directions for development in Lao PDR	Tropical animal health and production	
233.	N. Takeuchi-Storm; M. Denwood; T. V. A. Hansen; T. Halasa; E. Rattenborg; J. Boes; H. L. Enemark; S. M. Thamsborg	2017	Farm-level risk factors for Fasciola hepatica infection in Danish dairy cattle as evaluated by two diagnostic methods	Parasit Vectors	10.1186/s13071-017-2504-y
234.	V. Simova; E. Voslarova; V. Vecerek; A. Passantino; I. Bedanova	2017	Effects of travel distance and season of the year on transport-related mortality in cattle	Animal science journal = Nihon chikusan Gakkaiho	
235.	S. J. Schuetze	2017	Review: Transportation of commercial finished cattle and animal welfare considerations	Professional animal scientists. 2017 Oct., v. 33, no. 5	
236.	K. J. Schiffer; S. K. Retz; B. Algers; O. Hensel	2017	Assessment of stun quality after gunshot used on cattle: a pilot study on effects of diverse ammunition on physical signs displayed after the shot, brain tissue damage and brain haemorrhages	Animal Welfare	10.7120/09627286.26.1.095
237.	L. Scherer; B. Tomasik; O. Rueda; S. Pfister	2017	Framework for integrating animal welfare into life cycle sustainability assessment	The international journal of life cycle assessment	
238.	J. K. M. Probst, Eric: Spengler Neff, Anet	2017	Auswirkungen von Stressoren vor der Schlachtung auf Rinder bei zwei verschiedenen Schlachtmethoden (Bolzenschuss im kleinen Schlachthof und Kugelschuss auf der Weide)		

239.	P. S. Pozzi; W. Geraisy; M. P. Markovich	2017	Observation of Certain Parameters with Animal Welfare Consequences During the Implementation of Shechita	Israel Journal of Veterinary Medicine	
240.	T. Perruchoud; A. Maeschli; H. Bachmann; M. Walkenhorst; G. Schüpbach; M. Mevissen; P. Zanolari	2017	Diagnose, Therapie und prophylaktische Massnahmen der Gebärparese beim Rind<br><br>Ergebnisse der Online-Umfrage bei Schweizer Tierärzten		
241.	J. Pempek; D. Trearchis; M. Masterson; G. Habing; K. Proudfoot	2017	Veal calf health on the day of arrival at growers in Ohio	J Anim Sci	10.2527/jas2017.1642
242.	S. E. O. Oliveira; N. G. Gregory; F. A. Dalla Costa; T. J. Gibson; M. J. R. Paranhos da Costa	2017	Efficiency of low versus high airline pressure in stunning cattle with a pneumatically powered penetrating captive bolt gun	Meat Sci	10.1016/j.meatsci.2017.04.007
243.	Y. Z. Njisane; V. Muchenje	2017	Pre-slaughter effects on bleed-out times and some behavioural and physiological responses of Nguni and non-descript steers	South African Journal of Animal Science	10.4314/sajas.v47i1.12
244.	Y. Z. Njisane; V. Muchenje	2017	Farm to abattoir conditions, animal factors and their subsequent effects on cattle behavioural responses and beef quality - A review	Asian-Australasian journal of animal sciences	
245.	S. S. Nielsen; M. J. Denwood; B. Forkman; H. Houe	2017	Selection of Meat Inspection Data for an Animal Welfare Index in Cattle and Pigs in Denmark	Animals : an open access journal from MDPI	
246.	S. Nampanya	2017	Smallholder large ruminant health and production in Lao PDR: challenges and opportunities for improving domestic and regional beef supply	Animal production science. 2017, v. 57, no. 6	
247.	S. More; D. Bicout; A. Botner; A. Butterworth; P. Calistri; K. Depner; S. Edwards; B. Garin-Bastuji; M. Good; C. G. Schmidt; V. Michel; M. A. Miranda; S. S. Nielsen; A. Velarde; H. H. Thulke; L. Sihvonen; H. Spoolder; J. A. Stegeman; M. Raj; P. Willeberg; D. Candiani; C. Winckler; E. P. A. H. W. AHAW	2017	Animal welfare aspects in respect of the slaughter or killing of pregnant livestock animals (cattle, pigs, sheep, goats, horses)	Efsa Journal	10.2903/j.efsa.2017.4782
248.	M. A. Moggy; E. A. Pajor; W. E. Thurston; S. Parker; A. M. Greter; K. S. Schwartzkopf-	2017	Management practices associated with stress in cattle on western Canadian cow-calf operations: A mixed methods study	Journal of animal science	

	Genswein; J. R. Campbell; M. C. Windeyer				
249.	G. C. Miranda-de la Lama; L. X. Estevez-Moreno; W. S. Sepulveda; M. C. Estrada-Chavero; A. A. Rayas-Amor; M. Villarroel; G. A. Maria	2017	Mexican consumers' perceptions and attitudes towards farm animal welfare and willingness to pay for welfare friendly meat products	Meat Science	10.1016/j.meatsci.2016.12.001
250.	P. L. Meyer-Glitza, Edmund	2017	Zweijährige Laktationen durch einmal ausgesetzte Kalbung eine Fallstudie		
251.	D. McLean; L. Meers; J. Ralph; J. S. Owen; A. Small	2017	Development of a microwave energy delivery system for reversible stunning of cattle	Research in veterinary science	
252.	S. P. McCulloch; M. J. Reiss	2017	The Development of an Animal Welfare Impact Assessment (AWIA) Tool and Its Application to Bovine Tuberculosis and Badger Control in England	Journal of Agricultural & Environmental Ethics	10.1007/s10806-017-9684-5
253.	S. P. McCulloch	2017	Bovine Tuberculosis and Badger Culling in England: An Animal Rights-Based Analysis of Policy Options	Journal of agricultural & environmental ethics. 2017 Aug., v. 30, no. 4	
254.	S. P. McCulloch	2017	Bovine Tuberculosis Policy in England: Would a Virtuous Government Cull Mr Badger?	Journal of agricultural & environmental ethics. 2017 Aug., v. 30, no. 4	
255.	S. Marti; R. E. Wilde; D. Moya; C. E. M. Heuston; F. Brown; K. S. Schwartzkopf-Genswein	2017	Effect of rest stop duration during long-distance transport on welfare indicators in recently weaned beef calves	Journal of animal science	
256.	S. Marti; J. A. Jackson; N. Slootmans; E. Lopez; A. Hodge; M. Pérez-Juan; M. Devant; S. Amatayakul-Chantler	2017	Effects on performance and meat quality of Holstein bulls fed high concentrate diets without implants following immunological castration	Meat Sci	10.1016/j.meatsci.2016.11.013
257.	S. March; A. Bergschmidt; C. Renziehausen; J. Brinkmann	2017	Indikatoren für eine ergebnisorientierte Honorierung von Tierschutzleistungen		
258.	M.-S. A. Manuel; J. M. Simon; B. M. David; J. H. Alison	2017	Challenges facing the veterinary profession in Ireland<br><br>3. emergency and casualty slaughter certification	Irish Veterinary Journal, Vol 70, Iss 1, Pp 1-	
259.	V. I. Levakhin; I. F. Gorlov; E. A. Azhmuldinov; Y. I. Levakhin;	2017	Change in physiological parameters of calves of various breeds under the transport and pre-slaughter stress	Nusantara Bioscience	10.13057/nusbiosci/n090101

	G. K. Duskaev; E. Y. Zlobina; E. V. Karpenko				
260.	M. P. Keane; M. McGee; E. G. O'Riordan; A. K. Kelly; B. Earley	2017	Effect of space allowance and floor type on performance, welfare and physiological measurements of finishing beef heifers	Animal : an international journal of animal bioscience	
261.	V. Juan Pérez; R.-B. Wilberto; P. René Patiño; B.-A. Luz	2017	Diagnostic study of bovine welfare during transport to a beneficiary plant in the Caribbean region of Colombia	Revista Colombiana de Ciencia Animal Recia, Vol 9, Iss 2, Pp 323-	
262.	D. L. Hoeksma; M. A. Gerritzen; A. M. Lokhorst; P. M. Poortvliet	2017	An extended theory of planned behavior to predict consumers' willingness to buy mobile slaughter unit meat	Meat science	
263.	M. S. Herskin; A. Hels; I. Anneberg; P. T. Thomsen	2017	Livestock drivers' knowledge about dairy cow fitness for transport - A Danish questionnaire survey	Research in veterinary science	
264.	J. A. Hagenmaier; C. D. Reinhardt; S. J. Bartle; J. N. Henningson; M. J. Ritter; M. S. Calvo-Lorenzo; G. J. Vogel; C. A. Guthrie; M. G. Siemens; D. U. Thomson	2017	Effect of handling intensity at the time of transport for slaughter on physiological response and carcass characteristics in beef cattle fed ractopamine hydrochloride	Journal of animal science	
265.	H. Greter; A. A. Batil; B. N. Ngandolo; I. O. Alfaroukh; D. D. Moto; J. Hattendorf; J. Utzinger; J. Zinsstag	2017	Human and livestock trematode infections in a mobile pastoralist setting at Lake Chad: added value of a One Health approach beyond zoonotic diseases research	Transactions of the Royal Society of Tropical Medicine and Hygiene	
266.	T. Grandin	2017	A Major Change	Animal Ethics Reader, 3rd Edition	
267.	T. Grandin	2017	On-farm conditions that compromise animal welfare that can be monitored at the slaughter plant	Meat Sci	10.1016/j.meatsci.2017.05.004
268.	H. H. Giese	2017	Integration von simulations-basierten Trainings zum Erwerb der praktischen Fertigkeit der transrektalen gynäkologischen Untersuchung beim Rind unter Berücksichtigung von Tierwohlaspekten		
269.	T. J. Gibson; E. L. Jackson	2017	The economics of animal welfare	Revue Scientifique Et Technique-Office International Des Epizooties	10.20506/rst.36.1.2616
270.	S. Gehringer; M. Müller; J. Maierl	2017	Morphologische Untersuchungen tiefreichender Klauenerkrankungen beim Rind	Tierärztliche Praxis G: Großtiere/Nutztiere	

271.	F. Fusi; A. Angelucci; V. Lorenzi; L. Bolzoni; L. Bertocchi	2017	Assessing circumstances and causes of dairy cow death in Italian dairy farms through a veterinary practice survey (2013-2014)	Prev Vet Med	10.1016/j.prevetmed.2017.01.004
272.	A. Fuseini; S. B. Wotton; P. J. Hadley; T. G. Knowles	2017	The compatibility of modern slaughter techniques with halal slaughter: a review of the aspects of 'modern' slaughter methods that divide scholarly opinion within the Muslim community	Animal Welfare	10.7120/09627286.26.3.301
273.	A. Fuseini; S. B. Wotton; P. J. Hadley; T. G. Knowles	2017	The perception and acceptability of pre-slaughter and post-slaughter stunning for Halal production: The views of UK Islamic scholars and Halal consumers	Meat science	
274.	M. Freick; A. Kunze; O. Passarge; J. Weber; S. Geidel	2017	Metritis vaccination in Holstein dairy heifers using a herd-specific multivalent vaccine - Effects on uterine health and fertility in first lactation	Animal reproduction science	
275.	L. F. S. Fonseca; D. F. J. Gimenez; D. B. Dos Santos Silva; R. Barthelson; F. Baldi; J. A. Ferro; L. G. Albuquerque	2017	Differences in global gene expression in muscle tissue of Nellore cattle with divergent meat tenderness	BMC Genomics	10.1186/s12864-017-4323-0
276.	B. Earley; K. Buckham Sporer; S. Gupta	2017	Invited review: Relationship between cattle transport, immunity and respiratory disease	Animal : an international journal of animal bioscience	
277.	C. A. Duthie; M. Haskell; J. J. Hyslop; A. Waterhouse; R. J. Wallace; R. Roehe; J. A. Rooke	2017	The impact of divergent breed types and diets on methane emissions, rumen characteristics and performance of finishing beef cattle	Animal	10.1017/s1751731117000301
278.	K. Dieho	2017	Aspects of rumen adaptation in dairy cattle<br><br>morphological, functional, and gene expression changes of the rumen papillae and changes of the rumen microbiota during the transition period		
279.	A. Demartoto; R. B. Soemanto; S. Zunariyah	2017	Zoo agent's measure in applying the five freedoms principles for animal welfare	Veterinary World	10.14202/vetworld.2017.1026-1034
280.	M. S. Dawkins	2017	Animal welfare and efficient farming: is conflict inevitable?	Animal Production Science	10.1071/an15383
281.	U. Dämmgen; W. Brade; H.-D. Haenel; C. Rösemann; J. Dämmgen; U. Meyer	2017	Emissionen aus der Milchrinderhaltung und ihre Beeinflussung durch das Herdenmanagement		
282.	P. D'Amico; N. Vitelli; B. Cenci Goga; D. Nucera; F. Pedonese; A. Guidi; A. Armani	2017	Meat from cattle slaughtered without stunning sold in the conventional market without appropriate labelling: A case study in Italy	Meat science	

283.	R. G. Cruz-Monterrosa; V. Resendiz-Cruz; A. A. Rayas-Amor; M. Lopez; G. C. Miranda-de La Lama	2017	Bruises in beef cattle at slaughter in Mexico: implications on quality, safety and shelf life of the meat	Tropical Animal Health and Production	10.1007/s11250-016-1173-8
284.	E. Cresswell; J. Remnant; A. Butterworth; W. Wapenaar	2017	Injection-site lesion prevalence and potential risk factors in UK beef cattle	The Veterinary record	
285.	A. Y. Chulayo; V. Muchenje	2017	Activities of some stress enzymes as indicators of slaughter cattle welfare and their relationship with physico-chemical characteristics of beef	Animal : an international journal of animal bioscience	
286.	I. B. Christoph-Schulz; A.-K. Rovers; N. Brümmer; D. Saggau	2017	SocialLab - Nutztierhaltung im Spiegel der Gesellschaft		
287.	E. Byrd; N. O. Widmar; J. Fulton	2017	Of Fur, Feather, and Fin: Human's Use and Concern for Non-Human Species	Animals : an open access journal from MDPI	
288.	J. Broucek; M. Uhrincat; S. Mihina; M. Soch; A. Mrekajova; A. Hanus	2017	Dairy Cows Produce Less Milk and Modify Their Behaviour during the Transition between Tie-Stall to Free-Stall	Animals	10.3390/ani7030016
289.	M. E. Berry	2017	Ensuring safety and quality in the production of beef Volume 1<br><br>Safety	Burleigh Dodds Series in Agricultural Science	
290.	M. Baldassarre; M. Naldi; M. Domenicali; S. Volo; M. Pietra; F. Dondi; P. Caraceni; A. Peli	2017	Simple and rapid LC-MS method for the determination of circulating albumin microheterogeneity in veal calves exposed to heat stress	Journal of pharmaceutical and biomedical analysis	
291.	E. Armano	2017	Mapping Precariousness, Labour Insecurity and Uncertain Livelihoods<br><br>Subjectivities and Resistance		
292.	T. S. Waghorn; C. M. Miller; D. M. Leathwick	2016	Confirmation of ivermectin resistance in Ostertagia ostertagi in cattle in New Zealand	Veterinary parasitology	
293.	M. Verhoeven	2016	Assessing unconsciousness in livestock at slaughter		
294.	J. Twardoń; W. Zalewski; T. Nowicki; B. Dębski; G. Dejneka; J. Mrowiec; K. Zalewski	2016	The investigation of correlation among selected biochemical parameters and vital signs in dairy herd to design the bio-cybernetic dairy cow model	Pol J Vet Sci	10.1515/pjvs-2016-0087
295.	T. R. Troxel	2016	Temperature, relative humidity, and dew point of 6 commercial trailer compartments during summer transportations of beef calves in the mid-South	Professional animal scientists. 2016 Aug., v. 32, no. 4	

296.	C. Terlouw; C. Bourguet; V. Deiss	2016	Consciousness, unconsciousness and death in the context of slaughter. Part II. Evaluation methods	Meat science	
297.	D. Stoian; A. Rodas; J. Arguello	2016	Prosperity prospects in contested forest areas<br><br>evidence from community forestry development in Guatemala and Nicaragua		
298.	D. Sorin Daniel; R. Oana Lucia; M. Marian; H. Gilad; T. Alexandra	2016	A Comparison Between Ritual and Conventional Slaughter in Regard to Animal Welfare and Meat Hygienic Quality	Bulletin of University of Agricultural Sciences and Veterinary Medicine Cluj-Napoca: Veterinary Medicine, Vol 73, Iss 2, Pp 376-	
299.	V. Simova; E. Voslarova; A. Passantino; I. Bedanova; V. Vecerek	2016	Mortality rates in different categories of cattle during transport for slaughter	Berliner Und Munchener Tierarztliche Wochenschrift	10.2376/0005-9366-15106
300.	V. Simova; V. Vecerek; A. Passantino; E. Voslarova	2016	Pre-transport factors affecting the welfare of cattle during road transport for slaughter - a review	Acta Veterinaria Brno	10.2754/avb201685030303
301.	G. E. Simon; B. R. Hoar; C. B. Tucker	2016	Assessing cow-calf welfare. Part 1: Benchmarking beef cow health and behavior, handling; and management, facilities, and producer perspectives	Journal of animal science	
302.	B. K. Schwenk; I. Lechner; S. G. Ross; D. Gascho; B. P. Kneubuehl; M. Glardon; M. H. Stoffel	2016	Magnetic resonance imaging and computer tomography of brain lesions in water buffaloes and cattle stunned with handguns or captive bolts	Meat science	
303.	K. Schwartzkopf-Genswein	2016	Symposium Paper: Transportation issues affecting cattle well-being and considerations for the future11Presented at the Cattle Transportation Symposium sponsored by the Beef Checkoff Program, National Cattlemen_s Beef Association, and Colorado State University, Ft. Collins, Colorado, May 2015	Professional animal scientists. 2016 Dec., v. 32, no. 6	
304.	M. H. Santana; M. C. Freua; D. N. Do; R. V. Ventura; H. N. Kadarmideen; J. B. Ferraz	2016	Systems genetics and genome-wide association approaches for analysis of feed intake, feed efficiency, and performance in beef cattle	Genet Mol Res	10.4238/gmr15048930
305.	M. Ross; T. M. Widowski; D. B. Haley	2016	The effects of feeding space on the behavioural responses of cattle during rest periods offered as part of long-distance transportation	Animal Welfare	10.7120/09627286.25.2.217

306.	E. Razzuoli; E. Olzi; P. Calà; S. Cafazzo; D. Magnani; A. Vitali; N. Lacetera; L. Archetti; F. Lazzara; A. Ferrari; L. Nanni Costa; M. Amadori	2016	Innate immune responses of young bulls to a novel environment	Veterinary immunology and immunopathology	
307.	N. D. Otten; T. Rousing; H. Houe; P. T. Thomsen; J. T. Sorensen	2016	Comparison of animal welfare indices in dairy herds based on different sources of data	Animal Welfare	10.7120/09627286.25.2.207
308.	C. Orgel; I. Ruddat; M. Hoedemaker	2016	Prävalenz von Lahmheiten unterschiedlichen Grades in der Frühlaktation von Milchkühen und deren Einfluss auf Fruchtbarkeitsparameter	Tierarztliche Praxis. Ausgabe G, Grosstiere/Nutztiere	
309.	J. Okajima; K. Shibata; E. Takahashi; T. Nagafuchi; K. Okajima; N. Nonaka	2016	Current status and its epidemiological consideration of Fasciola and Eurytrema infections in beef cattle of Japan	J Vet Med Sci	10.1292/jvms.15-0469
310.	S. Novelli; P. Sechi; S. Mattei; M. F. Iulietto; G. B. T. Cenci	2016	Report on religious slaughter practices in Italy	Veterinaria Italiana	10.12834/VetIt.189.920.1
311.	I. Nastasijevic; I. Tomasevic; N. Smigic; D. Milicevic; Z. Petrovic; I. Djekic	2016	Hygiene assessment of Serbian meat establishments using different scoring systems	Food Control	10.1016/j.foodcont.2015.10.034
312.	H. Mollenhorst; P. B. M. Berentsen; H. Berends; W. J. J. Gerrits; J. M. de Boer	2016	Economic and environmental effects of providing increased amounts of solid feed to veal calves	Journal of Dairy Science	10.3168/jds.2014-9212
313.	M. A. S. Mitchell, H. A. M.	2016	Results of a desk study on best practices for animal transport		
314.	B. Mepham	2016	Morality, morbidity and mortality: an ethical analysis of culling nonhuman animals	End of Animal Life: A Start for Ethical Debate: Ethical and Societal Considerations on Killing Animals	10.3920/978-90-8686-808-7_8
315.	F. S. Mendonca; R. Z. Vaz; W. S. Leal; J. Restle; L. L. Pascoal; M. B. Vaz; G. D. Farias	2016	Genetic group and horns presence in bruises and economic losses in cattle carcasses	Semina-Ciencias Agrarias	10.5433/1679-0359.2016v37n6p4265
316.	P. McDermott; A. McKevitt	2016	Analysis of the operation of on farm emergency slaughter of bovine animals in the Republic of Ireland	Irish Veterinary Journal	10.1186/s13620-016-0063-8
317.	P. Maurer; E. Lucker; K. Riehn	2016	Slaughter of pregnant cattle in German abattoirs - current situation and prevalence: a cross-sectional study	Bmc Veterinary Research	10.1186/s12917-016-0719-3

318.	A. Marco-Ramell; A. M. de Almeida; S. Cristobal; P. Rodrigues; P. Roncada; A. Bassols	2016	Proteomics and the search for welfare and stress biomarkers in animal production in the one-health context	Molecular bioSystems	
319.	P. Malafaia; T. A. L. Granato; R. M. Costa; V. C. de Souza; D. F. A. Coste; C. H. Tokarnia	2016	Major health problems and their economic impact on beef cattle under two different feedlot systems in Brazil	Pesquisa Veterinaria Brasileira	10.1590/s0100-736x2016000900008
320.	M. Lava; G. Schupbach-Regula; A. Steiner; M. Meylan	2016	Antimicrobial drug use and risk factors associated with treatment incidence and mortality in Swiss veal calves reared under improved welfare conditions	Preventive Veterinary Medicine	10.1016/j.prevetmed.2016.02.002
321.	M. Lava; B. Pardon; G. Schüpbach-Regula; K. Keckeis; P. Deprez; A. Steiner; M. Meylan	2016	Effect of calf purchase and other herd-level risk factors on mortality, unwanted early slaughter, and use of antimicrobial group treatments in Swiss veal calf operations	Preventive veterinary medicine	
322.	J. L. Khol; T. Schafbauer; T. Wittek	2016	Zeitpunkt und Durchführung der tierschutzkonformen Nottötung beim Rind	Tierarztliche Praxis. Ausgabe G, Grosstiere/Nutztiere	
323.	A. Hund; T. Beer; T. Wittek	2016	Labmagenulzera bei Schlachtrindern in Österreich	Tierärztliche Praxis G: Großtiere/Nutztiere	
324.	L. E. Hulbert; S. J. Moisá	2016	Stress, immunity, and the management of calves	Journal of dairy science	
325.	A. C. Homem; M. C. Neto; R. S. B. Pinheiro; W. Koury; M. Estremote; L. Z. Camerro; A. C. Donofre; J. N. P. Puoli	2016	Influence of concentrate levels in diet and body biotypes on productive variables of Guzera beef cattle	Semina-Ciencias Agrarias	10.5433/1679-0359.2016v37n6p4305
326.	B. C. Hernandez-Cruz; A. A. Carrasco-Garcia; C. Ahuja-Aguirre; L. Lopez-deBuen; S. Rojas-Maya; F. Montiel-Palacios	2016	Faecal cortisol concentrations as indicator of stress during intensive fattening of beef cattle in a humid tropical environment	Tropical Animal Health and Production	10.1007/s11250-015-0966-5
327.	J. O. Hampton; B. Jones; A. L. Perry; C. J. Miller; Q. Hart	2016	Integrating animal welfare into wild herbivore management: lessons from the Australian Feral Camel Management Project	Rangeland Journal	10.1071/rj15079
328.	N. Hakansson; P. Flisberg; B. Algers; A. Jonsson; M. Ronnqvist; U. Wennergren	2016	Improvement of animal welfare by strategic analysis and logistic optimisation of animal slaughter transportation	Animal Welfare	10.7120/09627286.25.2.255
329.	M. Gotz	2016	Animal Welfare What makes Cattle afraid The Ethologist Temple Grandin sees the Trigger of the Slaughter Animals as Autistic	Fleischwirtschaft	

330.	J. N. Gilliam; J. K. Shearer; R. J. Bahr; S. Crochik; J. Woods; J. Hill; J. Reynolds; J. D. Taylor	2016	Evaluation of brainstem disruption following penetrating captive-bolt shot in isolated cattle heads: comparison of traditional and alternative shot-placement landmarks	Animal Welfare	10.7120/09627286.25.3.347
331.	N. Ghavi Hossein-Zadeh	2016	Effect of dystocia on subsequent reproductive performance and functional longevity in Holstein cows	Journal of animal physiology and animal nutrition	
332.	J. B. Garner; M. L. Douglas; S. R. Williams; W. J. Wales; L. C. Marett; T. T. Nguyen; C. M. Reich; B. J. Hayes	2016	Genomic Selection Improves Heat Tolerance in Dairy Cattle	Sci Rep	10.1038/srep34114
333.	C. B. Gallo; S. M. Huertas	2016	Main animal welfare problems in ruminant livestock during preslaughter operations: a South American view	Animal	10.1017/s1751731115001597
334.	A. Fuseini; T. G. Knowles; J. A. Lines; P. J. Hadley; S. B. Wotton	2016	The stunning and slaughter of cattle within the EU: a review of the current situation with regard to the halal market	Animal Welfare	10.7120/09627286.25.3.365
335.	A. Fuseini; T. G. Knowles; P. J. Hadley; S. B. Wotton	2016	Halal stunning and slaughter: Criteria for the assessment of dead animals	Meat Science	10.1016/j.meatsci.2016.04.033
336.	M. M. Farouk; K. M. Pufpaff; M. Amir	2016	Industrial halal meat production and animal welfare: A review	Meat Science	10.1016/j.meatsci.2016.04.023
337.	F. El Allaki; N. Harrington; K. Howden	2016	Assessing the sensitivity of bovine tuberculosis surveillance in Canada's cattle population, 2009-2013	Prev Vet Med	10.1016/j.prevetmed.2016.10.012
338.	R. E. Doyle; G. J. Coleman; D. M. McGill; M. Reed; W. Ramdani; P. H. Hemsworth	2016	Investigating the welfare, management and human-animal interactions of cattle in four Indonesian abattoirs	Animal Welfare	10.7120/09627286.25.2.191
339.	F. C. Dorea; F. Vial	2016	Animal health syndromic surveillance: a systematic literature review of the progress in the last 5 years (2011-2016)	Veterinary Medicine-Research and Reports	10.2147/vmrr.S90182
340.	R. J. Derscheid; R. D. Dewell; G. A. Dewell; K. E. Kleinhenz; L. C. Shearer; J. N. Gilliam; J. P. Reynolds; Y. Sun; J. K. Shearer	2016	Validation of a portable pneumatic captive bolt device as a one-step method of euthanasia for use in depopulation of feedlot cattle	Journal of the American Veterinary Medical Association	
341.	S. R. Clegg; J. Bell; S. Ainsworth; R. W. Blowey; N. J. Bell; S. D. Carter; N. J. Evans	2016	Isolation of digital dermatitis treponemes from cattle hock skin lesions	Veterinary dermatology	

342.	T. Clarke; J. R. Pluske; P. A. Fleming	2016	Are observer ratings influenced by prescription? A comparison of Free Choice Profiling and Fixed List methods of Qualitative Behavioural Assessment	Applied Animal Behaviour Science	10.1016/j.applanim.2016.01.022
343.	A. Y. Chulayo; G. Bradley; V. Muchenje	2016	Effects of transport distance, lairage time and stunning efficiency on cortisol, glucose, HSPA1A and how they relate with meat quality in cattle	Meat science	
344.	H. Boytchev	2016	Die Seucheninsel		
345.	J. W. Aleri; B. C. Hine; M. F. Pyman; P. D. Mansell; W. J. Wales; B. Mallard; A. D. Fisher	2016	Periparturient immunosuppression and strategies to improve dairy cow health during the periparturient period	Res Vet Sci	10.1016/j.rvsc.2016.07.007
346.	Z. A. Aghwan; A. U. Bello; A. A. Abubakar; J. C. Imlan; A. Q. Sazili	2016	Efficient halal bleeding, animal handling, and welfare: A holistic approach for meat quality	Meat Science	10.1016/j.meatsci.2016.06.028
347.	R. Zapf; U. Schultheiß; W. Achilles; L. Schrader; U. Knierim; H. J. Herrmann; J. Brinkmann; C. Winckler	2015	Indikatoren für die betriebliche Eigenkontrolle auf Tiergerechtheit _ Beispiel Milchkühe; Indicators for on-farm self-assessment of animal welfare _ Example dairy cows		
348.	C. Wrenzycki; H. Stinshoff	2015	Bedeutung der Biotechnologie beim Rind in Europa	Tierarztliche Praxis. Ausgabe G, Grosstiere/Nutztiere	
349.	K. Wilhelm; J. Wilhelm; M. Fürll	2015	Use of thermography to monitor sole haemorrhages and temperature distribution over the claws of dairy cattle	Vet Rec	10.1136/vr.101547
350.	T. Wilfred; E. N. Hezron	2015	A survey of the causes of cattle organs and/or carcass condemnation, financial losses and magnitude of foetal wastage at an abattoir in Dodoma, Tanzania	Onderstepoort Journal of Veterinary Research, Vol 82, Iss 1, Pp e1-e	
351.	J. Warren; A. R. Owen; A. Glanvill; A. Francis; G. Maboni; R. J. Nova; W. Wapenaar; C. Rees; S. Tötemeyer	2015	A new bovine conjunctiva model shows that Listeria monocytogenes invasion is associated with lysozyme resistance	Veterinary microbiology	
352.	F. Vial; S. Schärrer; M. Reist	2015	Risk factors for whole carcass condemnations in the Swiss slaughter cattle population	PloS one	
353.	M. T. W. Verhoeven, Gerritzen, M. A., Hellebrekers, L. J., Kemp, B.	2015	Indicators used in livestock to assess unconsciousness after stunning a review	Animal	

354.	M. Verdú; A. Bach; M. Devant	2015	Effect of concentrate feeder design on performance, eating and animal behavior, welfare, ruminal health, and carcass quality in Holstein bulls fed high-concentrate diets	Journal of animal science	
355.	D. van der Spek; J. A. van Arendonk; H. Bovenhuis	2015	Genetic relationships between claw health traits of dairy cows in different parities, lactation stages, and herds with different claw disorder frequencies	J Dairy Sci	10.3168/jds.2015-9561
356.	A. van der Linden; S. J. Oosting; G. W. J. van de Ven; I. J. M. de Boer; M. K. van Ittersum	2015	A framework for quantitative analysis of livestock systems using theoretical concepts of production ecology	Agricultural Systems	10.1016/j.agsy.2015.06.007
357.	C. B. Tucker; J. F. Coetzee; J. M. Stookey; D. U. Thomson; T. Grandin; K. S. Schwartzkopf-Genswein	2015	Beef cattle welfare in the USA: identification of priorities for future research	Animal health research reviews	
358.	P. R. Tozer; T. L. Marsh; E. V. Perevodchikov	2015	Economic Welfare Impacts of Foot-and-Mouth Disease in the Canadian Beef Cattle Sector	Canadian Journal of Agricultural Economics-Revue Canadienne D Agroeconomie	10.1111/cjag.12041
359.	F. Tiezzi; K. L. Parker-Gaddis; J. B. Cole; J. S. Clay; C. Maltecca	2015	A genome-wide association study for clinical mastitis in first parity US Holstein cows using single-step approach and genomic matrix re-weighting procedure	PloS one	
360.	D. U. Thomson; G. H. Loneragan; J. N. Henningson; S. Ensley; B. Bawa	2015	Description of a novel fatigue syndrome of finished feedlot cattle following transportation	Journal of the American Veterinary Medical Association	
361.	E. M. C. Terlouw; C. Bourguet; V. Deiss; C. Mallet	2015	Origins of movements following stunning and during bleeding in cattle	Meat science	
362.	E. S. Swai; A. A. Hayghaimo; A. A. Hassan; B. S. Mhina	2015	The slaughter of increased numbers of pregnant cows in Tanga abattoir, Tanzania: A cause for concern?	The Onderstepoort journal of veterinary research	
363.	M. Stock	2015	Tierschutz in der DDR<br><br>Hintergründe zur Entwicklung des Tierschutzes und seiner Organisation. Exemplarische Analyse der Haltungsbedingungen der Tierarten Rind und Schwein unter Tierschutzgesichtspunkten		
364.	S. Starosta	2015	Amtliche Schlachthofbefunde als Datengrundlage für ein Tierwohl-Monitoring<br><br>Potentiale und Grenzen		

365.	S. Starosta	2015	Potenziale derzeitiger Befunderhebung _ Verwendung der offiziellen Schlachttier- und Fleischuntersuchungsstatistik für einen Monitoring-Bericht der Tiergerechtheit		
366.	K. R. Stackhouse-Lawson; C. B. Tucker; M. S. Calvo-Lorenzo; F. M. Mitloehner	2015	Effects of growth-promoting technology on feedlot cattle behavior in the 21 days before slaughter	Applied Animal Behaviour Science	10.1016/j.applanim.2014.11.001
367.	J. Schwarz	2015	Klinisch-neurologische Untersuchungen zur Effektivität der Bolzenschussbetäubung bei Jungbullen und deren Potenzial zur Entwicklung eines automatischen Überwachungssystems ; Assessment of the efficiency of captive-bolt stunning in young bulls and the potential to develop an automatic monitoring system		
368.	K. J. Schiffer	2015	On-farm slaughter of cattle via gunshot method	Berichte aus der Agrarwissenschaft	
369.	J. K. M. Probst, Eric: Müller, Nils	2015	Das Pilotprojekt _Kugelschuss auf der Weide_ in der Schweiz		
370.	C. J. C. Phillips; J. C. Petherick	2015	The Ethics of a Co-regulatory Model for Farm Animal Welfare Research	Journal of Agricultural & Environmental Ethics	10.1007/s10806-014-9524-9
371.	C. Perez-Linares; A. Barreras; E. Sanchez; B. Herrera; F. Figueroa-Saavedra	2015	The effect of changing the pre-slaughter handling on bovine cattle DFD meat	Revista Mvz Cordoba	10.21897/rmvz.39
372.	G. Pannwitz	2015	Standardized analysis of German cattle mortality using national register data	Prev Vet Med	10.1016/j.prevetmed.2014.11.020
373.	T. Noffsinger; K. Lukasiewicz; L. Hyder	2015	Feedlot Processing and Arrival Cattle Management	The Veterinary clinics of North America. Food animal practice	
374.	S. Mullan	2015	Comments on the dilemma in the February issue: 'Emergency exsanguination of working equids'	In Practice	10.1136/inp.h972
375.	C. J. Mpamhanga; S. B. Wotton	2015	The effects of pre-slaughter restraint (for the purpose of cattle identification) on post-slaughter responses and carcass quality following the electrical stun/killing of cattle in a Jarvis Beef stunner	Meat science	
376.	D. Moya; R. Silasi; T. A. McAllister; B. Genswein; T.	2015	Use of pattern recognition techniques for early detection of morbidity in receiving feedlot cattle	Journal of Animal Science	10.2527/jas.2015-8907

	Crowe; S. Marti; K. S. Schwartzkopf-Genswein				
377.	C. Meyer	2015	Animal Welfare Feedback to Raise Beef farmers Interim results of a survey of the Association of the Reisch economy to slaughter Cattle bearing	Fleischwirtschaft	
378.	J. Loth	2015	Untersuchungen zur Belastung von Zuchtrindern bei langen Straßentransporten unter besonderer Berücksichtigung des Mikroklimas im Fahrzeug zu unterschiedlichen Jahreszeiten		
379.	S. H. Lee; J. Y. Lee; D. B. Han; R. M. Nayga	2015	Are Korean consumers willing to pay a tax for a mandatory BSE testing programme?	Applied Economics	10.1080/00036846.2014.993137
380.	M. P. Keane; M. McGee; E. G. O'Riordan; A. K. Kelly; B. Earley	2015	Effect of floor type on hoof lesions, dirt scores, immune response and production of beef bulls	Livestock Science	10.1016/j.livsci.2015.08.002
381.	C. B. Johnson; D. J. Mellor; P. H. Hemsworth; A. D. Fisher	2015	A scientific comment on the welfare of domesticated ruminants slaughtered without stunning	New Zealand veterinary journal	
382.	S. M. Huertas; F. van Eerdenburg; A. Gil; J. Piaggio	2015	Prevalence of carcass bruises as an indicator of welfare in beef cattle and the relation to the economic impact	Veterinary medicine and science	
383.	E. P. o. A. Health; Welfare	2015	Scientific Opinion on lumpy skin disease		
384.	N. S. Hayes; C. A. Schwartz; K. J. Phelps; P. Borowicz; K. R. Maddock-Carlin; R. J. Maddock	2015	The relationship between pre-harvest stress and the carcass characteristics of beef heifers that qualified for kosher designation	Meat science	
385.	J. L. Hardstaff; B. Hasler; J. R. Rushton	2015	Livestock trade networks for guiding animal health surveillance	Bmc Veterinary Research	10.1186/s12917-015-0354-4
386.	T. Grandin; C. Shivley	2015	How Farm Animals React and Perceive Stressful Situations Such As Handling, Restraint, and Transport	Animals (Basel)	10.3390/ani5040409
387.	A. Gomez; N. B. Cook; M. T. Socha; D. Döpfer	2015	First-lactation performance in cows affected by digital dermatitis during the rearing period	Journal of dairy science	
388.	C. Goldhawk; E. Janzen; L. A. González; T. Crowe; J. Kastelic; C. Kehler; M. Siemens; K.	2015	Trailer temperature and humidity during winter transport of cattle in Canada and evaluation of indicators used to assess the welfare of cull beef cows before and after transport	Journal of animal science	

	Ominski; E. Pajor; K. S. Schwartzkopf-Genswein				
389.	T. J. D. Gibson, Nikolaos; Gregory, Neville G.	2015	Effect of neck cut position on time to collapse in halal slaughtered cattle without stunning	Meat science	
390.	T. J. Gibson; C. W. Mason; J. Y. Spence; H. Barker; N. G. Gregory	2015	Factors Affecting Penetrating Captive Bolt Gun Performance	Journal of Applied Animal Welfare Science	10.1080/10888705.2014.980579
391.	M. A. Gerritzen; H. A. Verhoeven; V. A. Hindle	2015	Progress report validation of parameters to determine unconsciousness during slaughter of veal calves		
392.	M. A. Gerritzen	2015	Research concerning slaughter without stunning of ruminants in The Netherlands		
393.	A. B. Garcia; L. Shalloo	2015	Invited review: The economic impact and control of paratuberculosis in cattle	Journal of dairy science	
394.	B. Fufa Sorri; A. Samuel; G. Girma	2015	Effect of Transport Time of up to 12 Hours on Welfare of Cows and Bulls	Journal of Service Science and Management, Vol 08, Iss 02, Pp 161-	
395.	M. Freitag	2015	Slaughter cattle Gravid -Scope and Rear Grande of animal welfare relevant Problems	Tieraerztliche Umschau	
396.	D. Franco; A. Mato; F. J. Salgado; M. Lopez-Pedrouso; M. Carrera; S. Bravo; M. Parrado; J. M. Gallardo; C. Zapata	2015	Tackling proteome changes in the longissimus thoracis bovine muscle in response to pre-slaughter stress	Journal of Proteomics	10.1016/j.jprot.2015.03.029
397.	F. Fazio; V. Ferrantelli; A. Cicero; S. Casella; G. Piccione	2015	Utility of acute phase proteins as bionnarkers of transport stress in ewes and beef cattle	Italian Journal of Food Safety	10.4081/ijfs.2015.4210
398.	T. P. Elliott; J. M. Kelley; G. Rawlin; T. W. Spithill	2015	High prevalence of fasciolosis and evaluation of drug efficacy against Fasciola hepatica in dairy cattle in the Maffra and Bairnsdale districts of Gippsland, Victoria, Australia	Vet Parasitol	10.1016/j.vetpar.2015.02.014
399.	C. Egger-Danner; J. B. Cole; J. E. Pryce; N. Gengler; B. Heringstad; A. Bradley; K. F. Stock	2015	Invited review: overview of new traits and phenotyping strategies in dairy cattle with a focus on functional traits	Animal : an international journal of animal bioscience	

400.	J. G. Diez; C. Saraiva; A. C. Coelho	2015	Cattle farmers' perceptions about livestock policy	Large Animal Review	
401.	D. S. Collins; R. J. Huey	2015	Humane slaughter	Gracey's Meat Hygiene, 11th Edition	
402.	Y. Cheng; C. H. Chou; H. J. Tsai	2015	In vitro gene expression profile of bovine peripheral blood mononuclear cells in early Mycobacterium bovis infection	Exp Ther Med	10.3892/etm.2015.2814
403.	E. Burow	2015	Einfluss der Sommerweidehaltung auf das Tierwohl der Kühe _ Ergebnisse einer Studie über dänische Milchviehbetriebe		
404.	M. Brscic; R. Ricci; P. Prevedello; C. Lonardi; R. De Nardi; B. Contiero; F. Gottardo; G. Cozzi	2015	Synthetic rubber surface as an alternative to concrete to improve welfare and performance of finishing beef cattle reared on fully slatted flooring	Animal : an international journal of animal bioscience	
405.	M. Brscic; F. Gottardo; E. Tessitore; L. Guzzo; R. Ricci; G. Cozzi	2015	Assessment of welfare of finishing beef cattle kept on different types of floor after short- or long-term housing	Animal : an international journal of animal bioscience	
406.	B. M. Boyd; S. D. Shackelford; K. E. Hales; T. M. Brown-Brandl; M. L. Bremer; M. L. Spangler; T. L. Wheeler; D. A. King; G. E. Erickson	2015	Effects of shade and feeding zilpaterol hydrochloride to finishing steers on performance, carcass quality, heat stress, mobility, and body temperature	Journal of animal science	
407.	C. Bourguet; V. Deiss; A. Boissy; E. M. C. Terlouw	2015	Young Blond d'Aquitaine, Angus and Limousin bulls differ in emotional reactivity: Relationships with animal traits, stress reactions at slaughter and post-mortem muscle metabolism	Applied Animal Behaviour Science	10.1016/j.applanim.2014.12.009
408.	J. A. Boles; K. S. Kohlbeck; M. C. Meyers; K. A. Perz; K. C. Davis; J. M. Thomson	2015	The use of blood lactate concentration as an indicator of temperament and its impact on growth rate and tenderness of steaks from Simmental × Angus steers	Meat science	
409.	C. Berg; A. Botner; H. Browman; A. De Koeijer; K. Depner; M. Domingo; C. Ducrot; S. Edwards; C. Fourichon; F. Koenen; S. More; M. Raj; L. Sihvonen; H. Spoolder; J. A. Stegeman; H. H. Thulke; I. Vagsholm; A. Velarde; P. Willeberg; E. P. A. H. W. AHAW	2015	Enzootic bovine leukosis	Efsa Journal	10.2903/j.efsa.2015.4188

410.	B. Bartz; M. Collins; G. Stoddard; A. Appleton; R. Livingood; H. Sobcynski; K. D. Vogel	2015	Assessment of nonpenetrating captive bolt stunning followed by electrical induction of cardiac arrest in veal calves	Journal of animal science	
411.	T. Alföldi	2015	Video: Weideschlachtung - Eine tiergerechte und stressfreie Schlachtmethode (schweizerdeutsch)		
412.	L. F. S. Zuin; P. B. Zuin; A. G. Monzon; M. da Costa; I. R. Oliveira	2014	The multiple perspectives in a dialogical continued education course on animal welfare: Accounts of a team of extension agents and a manager and a cowboy from a rural Brazilian territory	Linguistics and Education	10.1016/j.linged.2014.07.003
413.	A. Wernicki; R. Urban-Chmiel; A. Puchalski; M. Dec	2014	Evaluation of the influence of transport and adaptation stress on chosen immune and oxidative parameters and occurrence of respiratory syndrome in feedlot calves	Bulletin of the Veterinary Institute in Pulawy	10.2478/bvip-2014-0018
414.	K. J. Visser; J. T. N. v. d. Werf; H. Gunnink; V. A. Hindle; J. W. v. Riel; I. D. E. v. Dixhoorn; M. A. Gerritzen	2014	Behaviour of heifers during long distance transport<br><br>comparison between two different transportation schemes		
415.	E. K. Visser; W. Ouweltjes; H. A. M. Spoolder	2014	Hazards and adverse effects for the assessment of animal welfare on farm and during transport<br><br>A preliminary table for bulls, veal calves and slaughter pigs		
416.	E. K. Visser; W. Ouweltjes; H. A. M. Spoolder	2014	Analysis of animal welfare risks from unloading until slaughter<br><br>red meat livestock species		
417.	E. K. Visser	2014	Note on minimum space allowance and compartment height for cattle and pigs during transport		
418.	F. Vial; M. Reist	2014	Evaluation of Swiss slaughterhouse data for integration in a syndromic surveillance system	BMC veterinary research	
419.	A. Velarde; P. Rodriguez; A. Dalmau; C. Fuentes; P. Llonch; K. V. von Holleben; M. H. Anil; J. B. Lambooij; H. Pleiter; T. Yesildere; B. T. Cenci-Goga	2014	Religious slaughter: evaluation of current practices in selected countries	Meat science	
420.	R. Tamura; T. Nemoto; T. Maruta; S. Onizuka; T.	2014	Up-regulation of NaV1.7 sodium channels expression by tumor necrosis factor-α in cultured bovine adrenal chromaffin cells and rat dorsal root ganglion neurons	Anesth Analg	10.1213/ane.0000000000000085

	Yanagita; A. Wada; M. Murakami; I. Tsuneyoshi				
421.	K. D. C. Stärk; S. Alonso; N. Dadios; C. Dupuy; L. Ellerbroek; M. Georgiev; J. Hardstaff; A. Huneau-Salaun; C. Laugier; A. Mateus; A. Nigsch; A. Afonso; A. Lindberg	2014	Strengths and weaknesses of meat inspection as a contribution to animal health and welfare surveillance	Food Control	
422.	B. K. Song; W. H. Nugent; P. F. Moon-Massat; R. N. Pittman	2014	Effects of a hemoglobin-based oxygen carrier (HBOC-201) and derivatives with altered oxygen affinity and viscosity on systemic and microcirculatory variables in a top-load rat model	Microvasc Res	10.1016/j.mvr.2014.07.005
423.	T. M. Sharp; S. R. McLeod; K. E. A. Leggett; T. J. Gibson	2014	Evaluation of a spring-powered captive bolt gun for killing kangaroo pouch young	Wildlife Research	10.1071/wr14094
424.	K. Schwartzkopf-Genswein; T. Grandin	2014	Cattle Transport by Road	Livestock Handling and Transport, 4th Edition	
425.	M. A. Schipp; A. D. Sheridan	2014	Applying the OIE Terrestrial Animal Health Code to the welfare of animals exported from Australia	Revue scientifique et technique (International Office of Epizootics)	
426.	K. J. Schiffer; S. K. Retz; U. Richter; B. Algers; O. Hensel	2014	Assessment of key parameters for gunshot used on cattle: a pilot study on shot placement and effects of diverse ammunition on isolated cattle heads	Animal Welfare	10.7120/09627286.23.4.479
427.	S. Schärrer; P. Presi; J. Hattendorf; N. Chitnis; M. Reist; J. Zinsstag	2014	Demographic model of the Swiss cattle population for the years 2009-2011 stratified by gender, age and production type	PloS one	
428.	F. Samuel; G. Girma; B. Emmanuel; D. A. Elias; H. Ibrahim	2014	Effect of Transportation and Pre-Slaughter Handling on Welfare and Meat Quality of Cattle<br><br>Case Study of Kumasi Abattoir, Ghana	Veterinary Sciences , Vol 1, Iss 3, Pp 174-	
429.	A. Robins; H. Pleiter; M. Latter; C. J. C. Phillips	2014	The efficacy of pulsed ultrahigh current for the stunning of cattle prior to slaughter	Meat Science	10.1016/j.meatsci.2013.10.030
430.	J. Rittirong; P. Prasartkul; R. R. Rindfuss	2014	From whom do older persons prefer support? The case of rural Thailand	Journal of aging studies	
431.	D. J. Rezac; D. U. Thomson; S. J. Bartle; J. B. Osterstock; F. L. Prouty; C. D. Reinhardt	2014	Prevalence, severity, and relationships of lung lesions, liver abnormalities, and rumen health scores measured at slaughter in beef cattle	Journal of animal science	

432.	J. L. Rault; P. H. Hemsworth; P. L. Cakebread; D. J. Mellor; C. B. Johnson	2014	Evaluation of microwave energy as a humane stunning technique based on electroencephalography (EEG) of anaesthetised cattle	Animal Welfare	10.7120/09627286.23.4.391
433.	J. K. Probst; A. S. Neff; E. Hillmann; M. Kreuzer; M. Koch-Mathis; F. Leiber	2014	Relationship between stress-related exsanguination blood variables, vocalisation, and stressors imposed on cattle between lairage and stunning box under conventional abattoir conditions	Livestock Science	10.1016/j.livsci.2014.03.013
434.	M. Pilz; C. Fischer-Tenhagen; M. Grau; W. Heuwieser	2014	Behavioural and physiological assessment of stress reactions during vaginal examination in dairy cows	Tierärztliche Praxis G: Großtiere/Nutztiere	
435.	A. M. Pérez-García; S. Oliván; R. Bover	2014	Subjective well-being in heart failure patients: influence of coping and depressive symptoms	International journal of behavioral medicine	
436.	T. Passler	2014	Euthanasia of farm animals	Farm Animal Anesthesia: Cattle, Small Ruminants, Camelids, and Pigs	
437.	W. Ouweltjes; E. K. Visser; B. Mounaix; S. Messori; M. A. Marahrens; K. Steinkamp; A. Velarde; A. Dalmau; C. Pedernera	2014	Welfare assessment protocol for cattle during transport		
438.	E. Olofsson; M. Noremark; S. S. Lewerin	2014	Patterns of between-farm contacts via professionals in Sweden	Acta Veterinaria Scandinavica	10.1186/s13028-014-0070-2
439.	J. K. Niemi; H. Lehtonen	2014	Livestock product trade and highly contagious animal diseases		
440.	C. D. Neely; D. U. Thomson; C. A. Kerr; C. D. Reinhardt	2014	Effects of three dehorning techniques on behavior and wound healing in feedlot cattle	Journal of Animal Science	10.2527/jas.2013-7424
441.	G. C. Miranda-de la Lama; M. Villarroel; G. A. María	2014	Livestock transport from the perspective of the pre-slaughter logistic chain: a review	Meat science	
442.	J. L. Lyles; M. S. Calvo-Lorenzo	2014	Bill E. Kunkle Interdisciplinary Beef Symposium: Practical developments in managing animal welfare in beef cattle: what does the future hold?	Journal of animal science	
443.	T. G. Knowles; P. D. Warriss; K. Vogel	2014	Stress Physiology of Animals During Transport	Livestock Handling and Transport, 4th Edition	
444.	E. King; L. Green	2014	Identification of indicators of cattle and sheep welfare in abattoirs	The Veterinary record	

445.	E. C. Jongman; K. L. Butler	2014	The Effect of Age, Stocking Density and Flooring during Transport on Welfare of Young Dairy Calves in Australia	Animals : an open access journal from MDPI	
446.	J. W. Hultgren, S.; Berg, C.; Cvek, K.; Kolstrup, C. L.	2014	Cattle behaviours and stockperson actions related to impaired animal welfare at Swedish slaughter plants	Applied Animal Behaviour Science	10.1016/j.applanim.2013.12.005
447.	S. M. Huertas; C. Gallo; F. Galindo	2014	Drivers of animal welfare policy in the Americas	Revue Scientifique Et Technique-Office International Des Epizooties	
448.	K. C. Horton; M. Wasfy; H. Samaha; B. Abdel-Rahman; S. Safwat; M. Abdel Fadeel; E. Mohareb; E. Dueger	2014	Serosurvey for zoonotic viral and bacterial pathogens among slaughtered livestock in Egypt	Vector Borne Zoonotic Dis	10.1089/vbz.2013.1525
449.	J. F. Hocquette; R. Botreau; I. Legrand; R. Polkinghorne; D. W. Pethick; M. Lherm; B. Picard; M. Doreau; E. M. C. Terlouw	2014	Win-win strategies for high beef quality, consumer satisfaction, and farm efficiency, low environmental impacts and improved animal welfare	Animal Production Science	10.1071/an14210
450.	E. F. Hannah; S. S.-G. Karen; G. B. Ken; B. H. Derek	2014	Characteristics of Loads of Cattle Stopping for Feed, Water and Rest during Long-Distance Transport in Canada	Animals, Vol 4, Iss 1, Pp 62-	
451.	T. Grandin	2014	Improving Welfare and Reducing Stress on Animals at Slaughter Plants	Livestock Handling and Transport, 4th Edition	
452.	T. Grandin	2014	A Whole Systems Approach to Assessing Animal Welfare During Handling and Restraint	Livestock Handling and Transport, 4th Edition	
453.	T. Grandin	2014	Animal welfare and society concerns finding the missing link	Meat Sci	10.1016/j.meatsci.2014.05.011
454.	C. Goldhawk; E. Janzen; L. A. González; T. Crowe; J. Kastelic; E. Pajor; K. S. Schwartzkopf-Genswein	2014	Trailer microclimate and calf welfare during fall-run transportation of beef calves in Alberta	Journal of animal science	
455.	C. Goldhawk; T. Crowe; L. A. González; E. Janzen; J. Kastelic; E. Pajor; K. Schwartzkopf-Genswein	2014	Comparison of eight logger layouts for monitoring animal-level temperature and humidity during commercial feeder cattle transport	Journal of animal science	
456.	A. Gavinelli; T. Kennedy; D. Simonin	2014	The application of humane slaughterhouse practices to large-scale culling	Revue Scientifique Et Technique-Office International Des Epizooties	10.20506/rst.33.1.2280

457.	W. K. Fulwider	2014	Dairy Cattle Behaviour, Facilities, Handling, Transport, Automation and Well-being	Livestock Handling and Transport, 4th Edition	
458.	A. R. Frattasi; L. Cesano; M. Botta; D. Pattono	2014	Assessment of the Farm Management of Culling Cattle: A Survey of Existing Practices and Suggestions for Drafting of Best Practices	Italian journal of food safety	
459.	N. H. Franco; M. Magalhaes-Sant'Ana; A. S. Olsson	2014	Welfare and Quantity of Life	Dilemmas in Animal Welfare	
460.	S. F. Foster; K. L. Overall	2014	The welfare of Australian livestock transported by sea	Vet J	10.1016/j.tvjl.2014.03.016
461.	A. D. Fisher; B. H. Stevens; M. J. Conley; E. C. Jongman; M. C. Lauber; S. J. Hides; G. A. Anderson; D. M. Duganzich; P. D. Mansell	2014	The effects of direct and indirect road transport consignment in combination with feed withdrawal in young dairy calves	The Journal of dairy research	
462.	C. C. Disanto, Giuseppe; Varvara, Michele; Fusiello, Nunziana; Fransvea, Armida; Bozzo, Giancarlo; Celano, Gaetano Vitale	2014	Stress Factors During Cattle Slaughter	Italian journal of food safety	
463.	N. Dadios; J. Hardstaff; S. Alonso; K. Staerk; A. Lindberg	2014	Disease monitoring in farmed game: the role of abattoir meat inspection	Trends in Game Meat Hygiene: From Forest to Fork	10.3920/978-90-8686-238-2_4
464.	M. da Costa; S. M. Huertas; A. C. Strappini; C. Gallo	2014	Handling and Transport of Cattle and Pigs in South America	Livestock Handling and Transport, 4th Edition	
465.	M. P. Caulfield; H. Cambridge; S. F. Foster; P. D. McGreevy	2014	Heat stress: a major contributor to poor animal welfare associated with long-haul live export voyages	Veterinary journal (London, England : 1997)	
466.	M. Brscic; P. Prevedello; A. L. Stefani; G. Cozzi; F. Gottardo	2014	Effects of the provision of solid feeds enriched with protein or nonprotein nitrogen on veal calf growth, welfare, and slaughter performance	Journal of dairy science	
467.	R. Blowey; N. Bell	2014	CATTLE WELFARE Rib fractures in slaughter cattle	Veterinary Record	10.1136/vr.g5500
468.	K. Alvåsen; A. Roth; M. Jansson Mörk; C. Hallén Sandgren; P. T. Thomsen; U. Emanuelson	2014	Farm characteristics related to on-farm cow mortality in dairy herds: a questionnaire study	Animal : an international journal of animal bioscience	
469.	K. Alvasen; M. J. Mork; I. R. Dohoo; C. H. Sandgren; P. T. Thomsen; U. Emanuelson	2014	Risk factors associated with on-farm mortality in Swedish dairy cows	Preventive Veterinary Medicine	10.1016/j.prevetmed.2014.08.011

470.	M. Ahsan; B. Hasan; M. Algotsson; S. Sarenbo	2014	Handling and welfare of bovine livestock at local abattoirs in Bangladesh	Journal of applied animal welfare science : JAAWS	
471.		2014	Maintaining local practices vital to TB control, says BVA President	The Veterinary record	
472.		2014	Slaughterhouse on wheels	The Veterinary record	
473.		2014	EFSA recommends a toolbox of indicators for monitoring welfare at slaughter	The Veterinary record	
474.	J. Zhao; J. X. Xu; J. W. Wang; N. Li	2013	Nutritional Composition Analysis of Meat From Human Lactoferrin Transgenic Bulls	Animal Biotechnology	10.1080/10495398.2012.739979
475.	M. Werner; C. Hepp; C. Soto; P. Gallardo; H. Bustamante; C. Gallo	2013	Effects of a long distance transport and subsequent recovery in recently weaned crossbred beef calves in Southern Chile	Livestock Science	10.1016/j.livsci.2012.12.007
476.	L. E. Webb; E. A. M. Bokkers; L. F. M. Heutinck; B. Engel; W. G. Buist; T. B. Rodenburg; N. Stockhofe-Zurwieden; C. G. v. Reenen	2013	Effects of roughage source, amount, and particle size on behavior and gastrointestinal health of veal calves	Journal of Dairy Science	
477.	D. van der Spek; J. A. van Arendonk; A. A. Vallée; H. Bovenhuis	2013	Genetic parameters for claw disorders and the effect of preselecting cows for trimming	J Dairy Sci	10.3168/jds.2013-6833
478.	C. M. Tiplady	2013	Public Response to Media Coverage of Animal Cruelty	Journal of agricultural and environmental ethics. 2013 Aug., v. 26, no. 4	
479.	P. T. Thomsen; J. T. Sorensen	2013	Does short-term road transport affect the locomotion score of dairy cows?	Animal Welfare	10.7120/09627286.22.4.445
480.	G. W. Thomas; P. Jordaan	2013	Pre-slaughter mortality and post-slaughter wastage in bobby veal calves at a slaughter premises in New Zealand	New Zealand veterinary journal	
481.	B. Teke	2013	Shrink and mortality of beef cattle during long distance transportation	Animal Welfare	10.7120/09627286.22.3.379
482.	A. C. Strappini; J. H. M. Metz; C. Gallo; K. Frankena; R. Vargas; I. de Freslon; B. Kemp	2013	Bruises in culled cows: when, where and how are they inflicted?	Animal : an international journal of animal bioscience	
483.	C. A. C. Stockman, T. Barnes, A. L. Miller, D. Wickham, S. L. Beatty, D. T. Blache, D. Wemelsfelder, F. Fleming, P. A.	2013	Flooring and driving conditions during road transport influence the behavioural expression of cattle	Applied Animal Behaviour Science	10.1016/j.applanim.2012.11.003

484.	D. Sorg; K. Danowski; V. Korenkova; V. Rusnakova; R. Küffner; R. Zimmer; H. H. D. Meyer; H. Kliem	2013	Microfluidic high-throughput RT-qPCR measurements of the immune response of primary bovine mammary epithelial cells cultured from milk to mastitis pathogens	Animal : an international journal of animal bioscience	
485.	A. Small; D. McLean; J. S. Owen; J. Ralph	2013	Electromagnetic induction of insensibility in animals: a review	Animal Welfare	10.7120/09627286.22.2.287
486.	B. B. Singh; S. Ghatak; H. S. Banga; J. P. S. Gill; B. Singh	2013	Veterinary urban hygiene: a challenge for India	Revue scientifique et technique (International Office of Epizootics)	
487.	L. A. Sinclair; K. J. Hart; D. Johnson; A. M. Mackenzie	2013	Effect of inorganic or organic copper fed without or with added sulfur and molybdenum on the performance, indicators of copper status, and hepatic mRNA in dairy cows	J Dairy Sci	10.3168/jds.2012-6322
488.	C. Senthilkumaran	2013	Increased annexin A1 and A2 levels in bronchoalveolar lavage fluid are associated with resistance to respiratory disease in beef calves	Veterinary research. 2013 Dec., v. 44, no. 1	
489.	A. Q. Sazili; B. Norbaiyah; I. Zulkifli; Y. M. Goh; M. Lotfi; A. H. Small	2013	Quality Assessment of Longissimus and Semitendinosus Muscles from Beef Cattle Subjected to Non-penetrative and Penetrative Percussive Stunning Methods	Asian-Australas J Anim Sci	10.5713/ajas.2012.12563
490.	G. Salamano; A. Cuccurese; A. Poeta; E. Santella; P. Sechi; V. Cambiotti; B. T. Cenci-Goga	2013	Acceptability of Electrical Stunning and Post-Cut Stunning Among Muslim Communities: A Possible Dialogue	Society & Animals	10.1163/15685306-12341310
491.	M. H. Romero; L. F. Uribe-Velásquez; J. A. Sánchez; G. C. Miranda-de la Lama	2013	Risk factors influencing bruising and high muscle pH in Colombian cattle carcasses due to transport and pre-slaughter operations	Meat science	
492.	C. J. Phillips; E. Santurtun	2013	The welfare of livestock transported by ship	Vet J	10.1016/j.tvjl.2013.01.007
493.	J. D. W. Nicholson; K. L. Nicholson; L. L. Frenzel; R. J. Maddock; R. J. Delmore; T. E. Lawrence; W. R. Henning; T. D. Pringle; D. D. Johnson; J. C. Paschal; R. J. Gill; J. J. Cleere; B. B. Carpenter; R. V. Machen; J. P. Banta; D. S. Hale; D. B. Griffin; J. W. Savell	2013	Survey of transportation procedures, management practices, and health assessment related to quality, quantity, and value for market beef and dairy cows and bulls	Journal of animal science	
494.	K. Nakyinsige; Y. B. C. Man; Z. A. Aghwan; I. Zulkifli; Y. M. Goh; F. Abu Bakar; H. A. Al-Kahtani; A. Q. Sazili	2013	Stunning and animal welfare from Islamic and scientific perspectives	Meat Science	10.1016/j.meatsci.2013.04.006

495.	M. Miele; J. Lever	2013	Civilizing the market for welfare friendly products in Europe? The techno-ethics of the Welfare Quality (R) assessment	Geoforum	10.1016/j.geoforum.2013.04.003
496.	R. McCorkell; K. Wynne-Edwards; J. Galbraith; A. Schaefer; N. Caulkett; S. Boysen; E. Pajor	2013	Transport versus on-farm slaughter of bison: Physiological stress, animal welfare, and avoidable trim losses	Canadian Veterinary Journal-Revue Veterinaire Canadienne	
497.	J. McBrien; E. A. Courcier	2013	Detection of Cysticercus bovis in abattoirs in Northern Ireland between 2001 and 2011	Vet Rec	10.1136/vr.101709
498.	G. la Lama; M. Pascual-Alonso; A. Guerrero; P. Alberti; S. Alierta; P. Sans; J. P. Gajan; M. Villarroel; A. Dalmau; A. Velarde; M. M. Campo; F. Galindo; M. P. Santolaria; C. Sanudo; G. A. Maria	2013	Influence of social dominance on production, welfare and the quality of meat from beef bulls	Meat Science	10.1016/j.meatsci.2013.03.026
499.	E. C. Jongman; K. L. Butler	2013	Ease of moving young calves at different ages	Australian veterinary journal	
500.	N. Jerez-Timaure; M. T. Sulbaran; L. A. de Moreno; A. Rodas-Gonzalez; J. Trompiz; J. Ortega	2013	Detection of quality failures in pork carcass and meat using farm and plant audit	Revista Mexicana De Ciencias Pecuarias	
501.	E. P. o. A. Health; Welfare	2013	Scientific Opinion on monitoring procedures at slaughterhouses for bovines	EFSA Journal, Vol 11, Iss	
502.	E. P. o. B. Hazards	2013	Scientific Opinion on the public health hazards to be covered by inspection of meat (bovine animals)	EFSA Journal, Vol 11, Iss	
503.	J. Häggman; J. Juga	2013	Genetic parameters for hoof disorders and feet and leg conformation traits in Finnish Holstein cows	J Dairy Sci	10.3168/jds.2012-6334
504.	T. Grandin	2013	Making slaughterhouses more humane for cattle, pigs, and sheep	Annual review of animal biosciences	
505.	M. M. Farouk	2013	Advances in the industrial production of halal and kosher red meat	Meat Science	10.1016/j.meatsci.2013.04.028
506.	B. Earley; M. Drennan; E. G. O'Riordan	2013	The effect of road transport in comparison to a novel environment on the physiological, metabolic and behavioural responses of bulls	Res Vet Sci	10.1016/j.rvsc.2013.04.027

507.	C. Dupuy; E. Morignat; X. Maugey; J. L. Vinard; P. Hendrikx; C. Ducrot; D. Calavas; E. Gay	2013	Defining syndromes using cattle meat inspection data for syndromic surveillance purposes: a statistical approach with the 2005-2010 data from ten French slaughterhouses	BMC Vet Res	10.1186/1746-6148-9-88
508.	K. Dörfler; K. Troeger; R. Lautenschläger; H. C. Schönekeß; F. Jäger; E. Lücker	2013	Kinetische Energie und Effektivität verschiedener Bolzenschuss-Betäubungsverfahren ; Kinetic energy and efficiency of different captive bolt stunning methods		
509.	K. Dörfler; K. Troeger; R. Lautenschläger; E. Lücker	2013	Wirksamkeit unterschiedlicher Bolzenschussapparate bei Schlachtrindern		
510.	K. Dorfler; M. Machtolf	2013	Slaughtering Technologies Animal Welfare requires new Solutions IFFA 2013: Developments in Cattle and Pig Slaughtering Technologies	Fleischwirtschaft	
511.	J. S. de Paula; I. C. G. Leite; A. B. de Almeida; G. M. B. Ambrosano; F. L. Mialhe	2013	The impact of socioenvironmental characteristics on domains of oral health-related quality of life in Brazilian schoolchildren	BMC oral health	
512.	R. F. Cooke; T. A. Guarnieri Filho; B. I. Cappellozza; D. W. Bohnert	2013	Rest stops during road transport: impacts on performance and acute-phase protein responses of feeder cattle	Journal of animal science	
513.	G. Cima	2013	Slaughter facilities won’t be able to hold downed calves	J Am Vet Med Assoc	
514.	N. Chapinal; A. Koeck; A. Sewalem; D. F. Kelton; S. Mason; G. Cramer; F. Miglior	2013	Genetic parameters for hoof lesions and their relationship with feet and leg traits in Canadian Holstein cows	J Dairy Sci	10.3168/jds.2012-6071
515.	B. T. Cenci-Goga; P. Sechi; A. Cuccurese; A. Poeta; G. De Angelis; P. Marini; C. Mattiacci; R. Rossi; R. Pezzato; G. Salamano; P. Santori	2013	Religious Slaughter: Data from Surveys and Spot-Check Visits in Italy and Animal Welfare Issues	Society & Animals	10.1163/15685306-12341311
516.	B. v. d. Bogert	2013	Community and genomic analysis of the human small intestine microbiota		
517.	A. Bassler; C. Arnould; A. Butterworth; L. Colin; I. C. d. Jong; V. Ferrante; P. Ferrari; S. A. Haslam; F. Wemelsfelder; H. J. Blokhuis	2013	Potential risk factors associated with contact dermatitis, lameness, negative emotional state, and fear of humans in broiler chicken flocks	Poultry Science	
518.	A. C. Barrier; M. J. Haskell; S. Birch; A. Bagnall; D. J. Bell; J. Dickinson; A. I. Macrae; C. M. Dwyer	2013	The impact of dystocia on dairy calf health, welfare, performance and survival	Veterinary journal (London, England : 1997)	

519.	S. Atkinson; A. Velarde; B. Algers	2013	Assessment of stun quality at commercial slaughter in cattle shot with captive bolt	Animal Welfare	10.7120/09627286.22.4.473
520.	D. N. Annen; C. Wieck; M. Kempen	2013	Animal Welfare in Public and Private Standards and On-Farm Compliance ; Tierwohl in staatlichen Vorschriften und privaten Standards und ihre Einhaltung auf dem landwirtschaftlichen Betrieb		
521.	S. Amatayakul-Chantler; F. Hoe; J. A. Jackson; R. O. Roca; J. E. Stegner; V. King; R. Howard; E. Lopez; J. Walker	2013	Effects on performance and carcass and meat quality attributes following immunocastration with the gonadotropin releasing factor vaccine Bopriva or surgical castration of Bos indicus bulls raised on pasture in Brazil	Meat science	
522.	M. von Wenzlawowicz; K. von Holleben; E. Eser	2012	Identifying reasons for stun failures in slaughterhouses for cattle and pigs: a field study	Animal Welfare	10.7120/096272812x13353700593527
523.	M. A. G. von Keyserlingk; A. Barrientos; K. Ito; E. Galo; D. M. Weary	2012	Benchmarking cow comfort on North American freestall dairies: lameness, leg injuries, lying time, facility design, and management for high-producing Holstein dairy cows	Journal of dairy science	
524.	A. D. Velarde, Antoni	2012	Animal welfare assessment at slaughter in Europe: moving from inputs to outputs	Meat science	
525.	M. Tinsley; F. I. Lewis; F. Brülisauer	2012	Network modeling of BVD transmission	Veterinary research	
526.	E. M. C. Terlouw; C. Bourguet; V. Deiss	2012	Stress at slaughter in cattle: role of reactivity profile and environmental factors	Animal Welfare	10.7120/096272812x13353700593482
527.	B. Techane; G. Girma; S. B. Fufa	2012	Animal Handling during Supply for Marketing and Operations at an Abattoir in Developing Country<br><br>The Case of Gudar Market and Ambo Abattoir, Ethiopia	Journal of Service Science and Management, Vol 05, Iss 01, Pp 59-	
528.	A. C. Strappini; K. Frankena; J. H. Metz; C. Gallo; B. Kemp	2012	Characteristics of bruises in carcasses of cows sourced from farms or from livestock markets	Animal	10.1017/s1751731111001698
529.	A. C. Strappini	2012	Bruises in Chilean cattle<br><br>their characterization, occurrence and relation with pre-slaughter conditions		
530.	R. J. Snyder; B. M. Perdue; D. M. Powell; D. L. Forthman; M. A. Bloomsmith; T. L. Maple	2012	Behavioral and Hormonal Consequences of Transporting Giant Pandas From China to the United States	Journal of Applied Animal Welfare Science	10.1080/10888705.2012.624046

531.	K. S. Schwartzkopf-Genswein	2012	Road transport of cattle, swine and poultry in North America and its impact on animal welfare, carcass and meat quality: A review	Meat science. 2012 Nov., v. 92, no. 3	
532.	A. L. Schaefer; N. J. Cook; C. Bench; J. B. Chabot; J. Colyn; T. Liu; E. K. Okine; M. Stewart; J. R. Webster	2012	The non-invasive and automated detection of bovine respiratory disease onset in receiver calves using infrared thermography	Res Vet Sci	10.1016/j.rvsc.2011.09.021
533.	B. E. Rollin	2012	Veterinary medical ethics. An ethicist's commentary on the treatment of downer animals	The Canadian veterinary journal = La revue vétérinaire canadienne	
534.	S. Robertson	2012	Convenience" euthanasia -- a comment"	The Canadian veterinary journal = La revue vétérinaire canadienne	
535.	P. Prevedello; M. Brscic; E. Schiavon; G. Cozzi; F. Gottardo	2012	Effects of the provision of large amounts of solid feeds to veal calves on growth and slaughter performance and intravitam and postmortem welfare indicators	Journal of animal science	
536.	J. M. Pestana; A. S. H. Costa; S. V. Martins; C. M. Alfaia; S. P. Alves; P. A. Lopes; R. J. B. Bessa; J. A. M. Prates	2012	Effect of slaughter season and muscle type on the fatty acid composition, including conjugated linoleic acid isomers, and nutritional value of intramuscular fat in organic beef	Journal of the science of food and agriculture	
537.	M. J. R. Paranhos da Costa	2012	Strategies to promote farm animal welfare in Latin America and their effects on carcass and meat quality traits	Meat science. 2012 Nov., v. 92, no. 3	
538.	H. A. O'Neill; E. C. Webb; L. Frylinck; P. Strydom	2012	Urinary catecholamine concentrations in three beef breeds at slaughter	South African Journal of Animal Science	10.4314/sajas.v42i5.21
539.	D. Muñoz; A. Strappini; C. Gallo	2012	Indicadores de bienestar animal para detectar problemas en el cajón de insensibilización de bovinos Animal welfare indicators to detect problems in the cattle stunning box	Archivos de Medicina Veterinaria, Vol 44, Iss 3, Pp 297-	
540.	G. C. Miranda-de la Lama; I. G. Leyva; A. Barreras-Serrano; C. Pérez-Linares; E. Sánchez-López; G. A. María; F. Figueroa-Saavedra	2012	Assessment of cattle welfare at a commercial slaughter plant in the northwest of Mexico	Tropical animal health and production	
541.	G. Limon; J. Guitian; N. G. Gregory	2012	A review of the humaneness of puntilla as a slaughter method	Animal Welfare	10.7120/096272812x13353700593248

542.	H. Leruste; M. Brscic; L. F. M. Heutinck; E. K. Visser; M. Wolthuis-Fillerup; E. A. M. Bokkers; N. Stockhofe-Zurwieden; G. Cozzi; F. Gottardo; B. J. Lensink; C. G. van Reenen	2012	The relationship between clinical signs of respiratory system disorders and lung lesions at slaughter in veal calves	Preventive veterinary medicine	
543.	S. M. Lee; J. Y. Kim; E. J. Kim	2012	Effects of Stocking Density or Group Size on Intake, Growth, and Meat Quality of Hanwoo Steers (Bos taurus coreanae)	Asian-Australasian Journal of Animal Sciences	10.5713/ajas.2012.12254
544.	E. Lambooij; J. T. N. van der Werf; H. G. M. Reimert; V. A. Hindle	2012	Compartment height in cattle transport vehicles	Livestock Science	10.1016/j.livsci.2012.05.014
545.	E. Lambooij, Werf, J. T. N. van der, Reimert, H. G. M., Hindle, V. A.	2012	Restraining and neck cutting or stunning and neck cutting of veal calves	Meat Science	
546.	I. N. K. Lage; P. V. R. Paulino; C. V. Pires; S. D. J. Villela; M. d. S. Duarte; S. d. C. Valadares Filho; M. F. Paulino; B. A. Maia; L. H. P. Silva; C. R. V. Teixeira	2012	Intake, digestibility, performance, and carcass traits of beef cattle of different gender	Tropical animal health and production	
547.	C. B. Johnson; T. J. Gibson; K. J. Stafford; D. J. Mellor	2012	Pain perception at slaughter	Animal Welfare	10.7120/096272812x13353700593888
548.	V. M. James; J. L. Gill; M. Topf; R. J. Harvey	2012	Molecular mechanisms of glycine transporter GlyT2 mutations in startle disease	Biological chemistry	
549.	Q. Ibrahim Hamdu; A. Elias; B. Emanuel; B. Techane; G. Girma; F. Samuel	2012	Animal Supply and Logistics Activities of Abattoir Chain in Developing Countries<br><br>The Case of Kumasi Abattoir, Ghana	Journal of Service Science and Management, Vol 05, Iss 01, Pp 20-	
550.	L. C. Hoffman; J. Lühl	2012	Causes of cattle bruising during handling and transport in Namibia	Meat science	
551.	S. J. Hauge; C. Kielland; G. Ringdal; E. Skjerve; O. Nafstad	2012	Factors associated with cattle cleanliness on Norwegian dairy farms	Journal of dairy science	
552.	C. J. Griffin	2012	Animal maiming, intimacy and the politics of shared life: the bestial and the beastly in eighteenth- and early nineteenth-century England	Transactions of the Institute of British Geographers	10.1111/j.1475-5661.2011.00464.x

553.	N. G. Gregory; M. von Wenzlawowicz; K. Holleben; H. R. Fielding; T. J. Gibson; L. Mirabito; R. Kolesar	2012	Complications during shechita and halal slaughter without stunning in cattle	Animal Welfare	10.7120/096272812x13353700593680
554.	T. Grandin	2012	Developing measures to audit welfare of cattle and pigs at slaughter	Animal welfare. 2012 Aug., v. 21, no. 3	
555.	T. Grandin	2012	Auditing animal welfare and making practical improvements in beef-, pork- and sheep-slaughter plants	Animal welfare. 2012 June, v. 21, no. 2	
556.	T. Grandin	2012	Effect of animal welfare audits of slaughter plants by a major fast food company on cattle handling and stunning practices	Journal of the American Veterinary Medical Association	
557.	L. A. González; K. S. Schwartzkopf-Genswein; M. Bryan; R. Silasi; F. Brown	2012	Space allowance during commercial long distance transport of cattle in North America	Journal of animal science	
558.	L. A. González; K. S. Schwartzkopf-Genswein; M. Bryan; R. Silasi; F. Brown	2012	Relationships between transport conditions and welfare outcomes during commercial long haul transport of cattle in North America	Journal of animal science	
559.	L. A. González; K. S. Schwartzkopf-Genswein; M. Bryan; R. Silasi; F. Brown	2012	Benchmarking study of industry practices during commercial long haul transport of cattle in Alberta, Canada	Journal of animal science	
560.	J. N. Gilliam	2012	Captive-bolt euthanasia of cattle: determination of optimal-shot placement and evaluation of the Cash Special Euthanizer Kit for euthanasia of cattle	Animal welfare. 2012 June, v. 21, no. 2	
561.	B. Gardiner	2012	Live exports in the spotlight again	Australian veterinary journal	
562.	R. S. Fries, K.; Lotz, F.; Arndt, G.	2012	Application of captive bolt to cattle stunning - a survey of stunner placement under practical conditions	Animal : an international journal of animal bioscience	
563.	L. Floyd	2012	HOW ABOLISHING (OR AT LEAST REFORMING) AUSTRALIA'S LIVE EXPORT CATTLE TRADE SUPPORTS HONG KONG ANIMAL LAW REFORM AND WHY THAT IS DESIRABLE FOR BOTH JURISDICTIONS	Hong Kong Law Journal	
564.	J. Efken; H. Bäurle; G. Haxsen; C. Niemann; C. Tamasy	2012	Der Markt für Fleisch und Fleischprodukte		
565.	B. Earley; M. Murray; D. J. Prendiville; B. Pintado; C. Borque; E. Canali	2012	The effect of transport by road and sea on physiology, immunity and behaviour of beef cattle	Research in veterinary science	

566.	R. Dwinger; B. Lambooij	2012	A brief summary of European legislation regarding animal welfare	Berliner und Munchener tierarztliche Wochenschrift	
567.	I. J. H. Duncan; M. Park; A. E. Malleau	2012	Global Animal Partnership's 5-Step (TM) Animal Welfare Rating Standards: a welfare-labelling scheme that allows for continuous improvement	Animal Welfare	10.7120/096272812x13345905673926
568.	P. Y. Daoust; C. Caraguel	2012	The Canadian harp seal hunt: observations on the effectiveness of procedures to avoid poor animal welfare outcomes	Animal Welfare	10.7120/09627286.21.4.445
569.	M. Cullinane	2012	Veterinary certificates for emergency or casualty slaughter bovine animals in the Republic of Ireland: are the welfare needs of certified animals adequately protected?	Animal welfare. 2012 June, v. 21, no. 2	
570.	L. M. Collins	2012	Welfare risk assessment: the benefits and common pitfalls	Animal welfare. 2012 May, v. 21, no. 1	
571.	G. J. Coleman	2012	Human-animal relationships at sheep and cattle abattoirs	Animal welfare. 2012 June, v. 21, no. 2	
572.	J. F. Coetzee; L. N. Edwards; R. A. Mosher; N. M. Bello; A. M. O'Connor; B. Wang; B. Kukanich; D. A. Blasi	2012	Effect of oral meloxicam on health and performance of beef steers relative to bulls castrated on arrival at the feedlot	Journal of animal science	
573.	G. Cima	2012	Supreme Court strikes down Calif. slaughter rules	Journal of the American Veterinary Medical Association	
574.	S. Cafazzo; D. Magnani; P. Cala; E. Razzuoli; G. Gerardi; D. Bernardini; M. Amadori; L. N. Costa	2012	Effect of short road journeys on behaviour and some blood variables related to welfare in young bulls	Applied Animal Behaviour Science	10.1016/j.applanim.2012.03.009
575.	M. Brscic; H. Leruste; L. F. M. Ruis-Heutinck; E. A. M. Bokkers; M. Wolthuis-Fillerup; N. Stockhofe; F. Gottardo; B. J. Lensink; G. Cozzi; C. G. v. Reenen	2012	Prevalence of respiratory disorders in veal calves and potential risk factors	Journal of Dairy Science	
576.	I. Blanco-Penedo; M. Lopez-Alonso; R. F. Shore; M. Miranda; C. Castillo; J. Hernandez; J. L. Benedito	2012	Evaluation of organic, conventional and intensive beef farm systems: health, management and animal production	Animal	10.1017/s1751731112000298

577.	A. C. Barrier	2012	Short communication: Survival, growth to weaning, and subsequent fertility of live-born dairy heifers after a difficult birth	Journal of dairy science. 2012 Nov., v. 95, no. 11	
578.	C. Bähler; A. Steiner; A. Luginbühl; A. Ewy; H. Posthaus; D. Strabel; T. Kaufmann; G. Regula	2012	Risk factors for death and unwanted early slaughter in Swiss veal calves kept at a specific animal welfare standard	Research in veterinary science	
579.	G. Arnott; D. Roberts; J. A. Rooke; S. P. Turner; A. B. Lawrence; K. M. D. Rutherford	2012	Board invited review: The importance of the gestation period for welfare of calves: maternal stressors and difficult births	Journal of animal science	
580.		2012	Consultation on proposals to stop testing healthy slaughter cattle for BSE	The Veterinary record	
581.	G. G. Yegutkin; M. Helenius; E. Kaczmarek; N. Burns; S. Jalkanen; K. Stenmark; E. V. Gerasimovskaya	2011	Chronic hypoxia impairs extracellular nucleotide metabolism and barrier function in pulmonary artery vasa vasorum endothelial cells	Angiogenesis	10.1007/s10456-011-9234-0
582.	C. Wülbeck	2011	Effekte von Natriumhydrogenkarbonat auf hochleistende Milchkühe in der Frühlaktation bei grassilagebasierten gemischten Rationen		
583.	K. D. Vogel; J. R. Claus; T. Grandin; G. R. Oetzel; D. M. Schaefer	2011	Effect of water and feed withdrawal and health status on blood and serum components, body weight loss, and meat and carcass characteristics of Holstein slaughter cows	Journal of animal science	
584.	I. Viegas; J. M. L. Santos; M. A. Fontes	2011	Joint Production of Safer, Cleaner and Animal Friendlier Beef<br><br>Do Consumers Join it Too? - Insights from Focus Groups		
585.	K. Uetake; T. Tanaka; S. Sato	2011	Effects of haul distance and stocking density on young suckling calves transported in Japan	Animal science journal = Nihon chikusan Gakkaiho	
586.	C. A. Stockman	2011	Qualitative behavioural assessment and quantitative physiological measurement of cattle naïve and habituated to road transport	Animal production science. 2011, v. 51, no. 3	
587.	J. K. Shearer; J. P. Reynolds	2011	Euthanasia Techniques for Dairy Cattle	Dairy Production Medicine	
588.	I. Schiller; W. R. Waters; W. RayWaters; H. M. Vordermeier; T. Jemmi; M. Welsh; N. Keck; A. Whelan; E. Gormley; M. L. Boschiroli; J. L. Moyen; C. Vela; M. Cagiola; B.	2011	Bovine tuberculosis in Europe from the perspective of an officially tuberculosis free country: trade, surveillance and diagnostics	Veterinary microbiology	

	M. Buddle; M. Palmer; T. Thacker; B. Oesch				
589.	R. A. Russell; J. M. Bewley	2011	Producer assessment of dairy extension programming in Kentucky	Journal of dairy science	
590.	J. Rushen; A. Butterworth; J. C. Swanson	2011	ANIMAL BEHAVIOR AND WELL-BEING SYMPOSIUM: Farm animal welfare assurance: Science and application	Journal of Animal Science	10.2527/jas.2010-3589
591.	K. Riehn; G. Domel; A. Einspanier; J. Gottschalk; G. Lochmann; G. Hildebrandt; J. Luy; E. Lucker	2011	Slaughter of pregnant cattle - aspects of ethics and consumer protection	Tieraerztliche Umschau	
592.	I. Patumi; L. Bortolotti; G. Manca; S. Marangon	2011	Database Application in Veterinary Medicine for Human and Animal Health Surveillance in the Veneto Region - Italy	Imcic'11: The 2nd International Multi-Conference on Complexity, Informatics and Cybernetics, Vol Ii	
593.	R. Odore; P. Badino; G. Re; R. Barbero; B. Cuniberti; A. D'Angelo; C. Girardi; E. Fraccaro; M. Tarantola	2011	Effects of housing and short-term transportation on hormone and lymphocyte receptor concentrations in beef cattle	Research in veterinary science	
594.	B. L. Nielsen	2011	Road transport of farm animals: effects of journey duration on animal welfare	Animal. 2011 Mar., v. 5, no. 3	
595.	S. P. Ndou; V. Muchenje; M. Chimonyo	2011	Animal welfare in multipurpose cattle production Systems and its implications on beef quality	African Journal of Biotechnology	
596.	G. C. Miranda-de la Lama	2011	Livestock vehicle accidents in Spain: Causes, consequences, and effects on animal welfare	Journal of applied animal welfare science. 2011 , v. 14, no. 2	
597.	K. M. Koenig; K. A. Beauchemin	2011	Optimum extent of barley grain processing and barley silage proportion in feedlot cattle diets: Growth, feed efficiency, and fecal characteristics	Canadian Journal of Animal Science	10.4141/cjas2010-039
598.	K. Klinglmair; K. B. Stevens; N. G. Gregory	2011	Luminance and glare in indoor cattle-handling facilities	Animal Welfare	
599.	M. A. Khan; D. M. Weary; M. A. von Keyserlingk	2011	Hay intake improves performance and rumen development of calves fed higher quantities of milk	J Dairy Sci	10.3168/jds.2010-3871

600.	J. Karugia; J. Wanjiku; J. Nzuma; S. Gbegbelegbe; E. Macharia; S. Massawe; H. A. Freeman; M. M. Waithaka; S. Kaitibie	2011	The impact of non-tariff barriers on maize and beef trade in East Africa		
601.	T. Herva; A. Huuskonen; A. M. Virtala; O. Peltoniemi	2011	On-farm welfare and carcass fat score of bulls at slaughter	Livestock Science	10.1016/j.livsci.2010.12.019
602.	P. H. Hemsworth; M. Rice; M. G. Karlen; L. Calleja; J. L. Barnett; J. Nash; G. J. Coleman	2011	Human-animal interactions at abattoirs: Relationships between handling and animal stress in sheep and cattle	Applied Animal Behaviour Science	10.1016/j.applanim.2011.09.007
603.	T. Greer; K. Schwartzkopf-Genswein; T. Crowe; L. A. Gonzalez	2011	The effect of transport distance on cattle liner microclimate, live weight loss and carcass quality of finished heifers during summer transport	Canadian Journal of Animal Science	
604.	K. L. Graunke; E. Telezhenko; A. Hessle; C. Bergsten; J. M. Loberg	2011	Does rubber flooring improve welfare and production in growing bulls in fully slatted floor pens?	Animal Welfare	
605.	C. Goldhawk; E. Janzen; L. A. Gonzalez; T. Crowe; E. Pajor; J. Kastelic; K. Schwartzkopf-Genswein	2011	Predicting internal trailer THI based on space allowance and ambient THI during transport of fall-weaned beef calves	Canadian Journal of Animal Science	
606.	C. Giannetto; F. Fazio; S. Casella; S. Marafioti; E. Giudice; G. Piccione	2011	Acute phase protein response during road transportation and lairage at a slaughterhouse in feedlot beef cattle	The Journal of veterinary medical science	
607.	B. Earley; B. McDonnell; M. Murray; D. J. Prendiville; M. A. Crowe	2011	The effect of sea transport from Ireland to the Lebanon on inflammatory, adrenocortical, metabolic and behavioural responses of bulls	Research in veterinary science	
608.	D. Demmler	2011	Leistungsabhängige Gesundheitsstörungen bei Nutztieren für die Fleischerzeugung (Schweine, Rinder, Hühner, Puten) und ihre Relevanz für § 11b Tierschutzgesetz (Qualzucht")"		
609.	L. T. U. d. S. A. s. M. V. a. B. T. Czister; S. Acatincai; E. N. Sossidou	2011	General knowledge of the romanian farmers about the farm animal welfare	Lucrari stiintifice. Seria Zootehnie - Universitatea de Stiinte Agricole si Medicina Veterinara Ion Ionescu de la Brad (Romania)	
610.	G. Cima	2011	Pushing for better welfare, pathology	Journal of the American Veterinary Medical Association	

611.	T. E. Carpenter; J. M. O'Brien; A. D. Hagerman; B. A. McCarl	2011	Epidemic and economic impacts of delayed detection of foot-and-mouth disease: a case study of a simulated outbreak in California	J Vet Diagn Invest	10.1177/104063871102300104
612.	M. Brscic; L. F. M. Heutinck; M. Wolthuis-Fillerup; N. Stockhofe; B. Engel; E. K. Visser; F. Gottardo; E. A. M. Bokkers; B. J. Lensink; G. Cozzi; C. G. Van Reenen	2011	Prevalence of gastrointestinal disorders recorded at postmortem inspection in white veal calves and associated risk factors	Journal of dairy science	
613.	A. Braghieri; C. Pacelli; G. De Rosa; A. Girolami; P. De Palo; F. Napolitano	2011	Podolian beef production on pasture and in confinement	Animal	10.1017/s1751731110002685
614.	C. Bourguet; V. Deiss; C. C. Tannugi; E. M. Terlouw	2011	Behavioural and physiological reactions of cattle in a commercial abattoir: relationships with organisational aspects of the abattoir and animal characteristics	Meat Sci	10.1016/j.meatsci.2010.12.017
615.	C. Bourguet; V. Deiss; A. Boissy; S. Andanson; E. M. C. Terlouw	2011	Effects of feed deprivation on behavioral reactivity and physiological status in Holstein cattle	Journal of Animal Science	10.2527/jas.2010-3139
616.	C. Bourguet, Deiss, Véronique, Tannugi, Carole Cohen, Terlouw, E. M. Claudia	2011	Behavioural and physiological reactions of cattle in a commercial abattoir: relationships with organisational aspects of the abattoir and animal characteristics	Meat science	
617.	R. W. Blowey	2011	Color atlas of diseases and disorders of cattle		
618.		2011	Welfare during transport: EFSA assesses the scientific evidence	The Veterinary record	
619.	L. A. Warren; I. B. Mandell; K. G. Bateman	2010	An audit of transport conditions and arrival status of slaughter cattle shipped by road at an Ontario processor	Canadian Journal of Animal Science	10.4141/cjas09068
620.	L. A. Warren; I. B. Mandell; K. G. Bateman	2010	Road transport conditions of slaughter cattle: Effects on the prevalence of dark, firm and dry beef	Canadian Journal of Animal Science	10.4141/cjas09091
621.	C. Vincenzo; R. Licia; C. Biagina; C. Leonardo Nanni; L. Luigi	2010	Effect of lairage duration on some blood constituents and beef quality in bulls after long journey	Italian Journal of Animal Science, Vol 6, Iss 4, Pp 375-	
622.	P. M. Thornber	2010	An Australian perspective on developing standards and ensuring compliance	Journal of veterinary medical education	
623.	A. C. Strappini; K. Frankena; J. H. M. Metz; B. Gallo; B. Kemp	2010	Prevalence and risk factors for bruises in Chilean bovine carcasses	Meat Science	10.1016/j.meatsci.2010.07.010

624.	T. M. Seeiso; C. M. E. McCrindle	2010	An investigation of the quality of meat sold in Lesotho	Journal of the South African Veterinary Association	
625.	K. S. Schwartzkopf-Genswein; L. A. Gonzalez; T. Crowe	2010	Cattle transport in North America-Current welfare research and future directions	Journal of Dairy Science	
626.	I. Schiller; B. Oesch; H. M. Vordermeier; M. V. Palmer; B. N. Harris; K. A. Orloski; B. M. Buddle; T. C. Thacker; K. P. Lyashchenko; W. R. Waters	2010	Bovine tuberculosis: a review of current and emerging diagnostic techniques in view of their relevance for disease control and eradication	Transboundary and emerging diseases	
627.	M. Saeb; H. Baghshani; S. Nazifi; S. Saeb	2010	Physiological response of dromedary camels to road transportation in relation to circulating levels of cortisol, thyroid hormones and some serum biochemical parameters	Tropical Animal Health and Production	10.1007/s11250-009-9385-9
628.	K. L. Proudfoot; D. M. Weary; M. A. von Keyserlingk	2010	Behavior during transition differs for cows diagnosed with claw horn lesions in mid lactation	J Dairy Sci	10.3168/jds.2009-2767
629.	C. J. C. Phillips; M. K. Pines; M. Latter; T. Muller; J. C. Petherick; S. T. Norman; J. B. Gaughan	2010	The physiological and behavioral responses of steers to gaseous ammonia in simulated long-distance transport by ship	Journal of animal science	
630.	D. J. Mellor	2010	Galloping Colts, Fetal Feelings, and Reassuring Regulations: Putting Animal-Welfare Science into Practice	Journal of Veterinary Medical Education	10.3138/jvme.37.1.94
631.	K. Linda, Elisabetta, Canali	2010	Welfare Quality® project, from scientific research to on farm assessment of animal welfare	Italian Journal of Animal Science, Vol 8, Iss 2s, Pp 900-	
632.	G. Limon; J. Guitian; N. G. Gregory	2010	An evaluation of the humaneness of puntilla in cattle	Meat science	
633.	E. Lambooij; W. H. M. Baltussen; M. H. Quintiliano	2010	The beef cattle welfare during transport<br><br>Brazilian experiences		
634.	I. Kolkman; S. Aerts; H. Vervaecke; J. Vicca; J. Vandelook; A. de Kruif; G. Opsomer; D. Lips	2010	Assessment of Differences in Some Indicators of Pain in Double Muscled Belgian Blue Cows Following Naturally Calving vs Caesarean Section	Reproduction in Domestic Animals	10.1111/j.1439-0531.2008.01295.x

635.	M. Iwersen	2010	Einfluss von Propylenglykol als Bestandteil einer Totalen Mischration während der Frühlaktation auf die Tier- und Stoffwechselgesundheit sowie auf Leistungsparameter von Milchkühen		
636.	T. Ikehara; H. Nishisako; Y. Minami; H. Ichinose Sasaki; T. Shiraishi; M. Kitamura; M. Shono; H. Houchi; K. Kawazoe; K. Minakuchi; K. Yoshizaki; Y. Kinouchi; H. Miyamoto	2010	Effects of exposure to a time-varying 1.5 T magnetic field on the neurotransmitter-activated increase in intracellular Ca(2+) in relation to actin fiber and mitochondrial functions in bovine adrenal chromaffin cells	Biochim Biophys Acta	10.1016/j.bbagen.2010.09.001
637.	S. M. Huertas; A. D. Gil; J. M. Piaggio; F. van Eerdenburg	2010	Transportation of beef cattle to slaughterhouses and how this relates to animal welfare and carcase bruising in an extensive production system	Animal Welfare	
638.	S. Hepple; G. Watkins; T. Crawshaw; D. Harwood; J. Ellis-Iversen; J. Clark; A. Pollock; T. Brough	2010	Risks to cattle transported long distances in late pregnancy	The Veterinary record	
639.	S. L. Gruber	2010	Relationships of behavioral and physiological symptoms of preslaughter stress to beef longissimus muscle tenderness	Journal of animal science. 2010 Mar., v. 88, no. 3	
640.	N. G. Gregory; H. R. Fielding; M. von Wenzlawowicz; K. von Holleben	2010	Time to collapse following slaughter without stunning in cattle	Meat science	
641.	M. Greger; S. Parente; M. C. Appleby; J. L. Lanier	2010	DISEASE AND TRANSPORT: A COSTLY TICKET AROUND THE WORLD	Handbook of Disease Outbreaks: Prevention, Detection and Control	
642.	T. Grandin	2010	Auditing animal welfare at slaughter plants	Meat Science	10.1016/j.meatsci.2010.04.022
643.	C. Giulio; R. Licia; F. Gianluca; R. Rebecca; G. Flaviana	2010	Welfare and meat quality of beef cattle housed on two types of floors with the same space allowance	Italian Journal of Animal Science, Vol 2, Iss 4, Pp 243-	
644.	B. Fuerst-Waltl; M. K. Sørensen	2010	Genetic analysis of calf and heifer losses in Danish Holstein	Journal of dairy science	
645.	N. Fabio; G. Antonio; S. Marcella; R. Giuseppe De; B. Ada	2010	Behaviour and meat quality of Podolian young bulls	Italian Journal of Animal Science, Vol 8, Iss 2s, Pp 598-	
646.	B. Earley; M. Murray; D. J. Prendiville	2010	Effect of road transport for up to 24 hours followed by twenty-four hour recovery on live weight and physiological responses of bulls	BMC veterinary research	

647.	B. Earley; M. Murray	2010	The effect of road and sea transport on inflammatory, adrenocortical, metabolic and behavioural responses of weanling heifers	BMC veterinary research	
648.	S. Durham	2010	Stress: It's Not Just for You and Me	Agricultural research. 2010 Aug., v. 58, no. 7	
649.	I. D. E. v. Dixhoorn; M. Kluivers-Poodt; E. A. A. Smolders; V. A. Hindle; E. Lambooij	2010	Fit for travel/Fitness during transport<br><br>Selectie van criteria om geschiktheid voor (lang) transport van varkens en rundvee te bepalen		
650.	M. Cullinane; E. O'Sullivan; G. Collins; D. M. Collins; S. J. More	2010	A review of bovine cases consigned under veterinary certification to emergency and casualty slaughter in Ireland during 2006 to 2008	Irish Veterinary Journal	10.1186/2046-0481-63-9-568
651.	J. F. Coetzee; R. Gehring; J. Tarus-Sang; D. E. Anderson	2010	Effect of sub-anesthetic xylazine and ketamine ('ketamine stun') administered to calves immediately prior to castration	Veterinary Anaesthesia and Analgesia	10.1111/j.1467-2995.2010.00573.x
652.	K. L. Casavant; M. R. Denicoff; E. Jessup; A. Taylor; D. Nibarger; D. Sears; H. Khachatryan; V. A. McCracken; M. E. Prater; J. O'Leary; N. Marathon; B. McGregor; S. Olowolayemo; B. Blanton	2010	Study of Rural Transportation Issues		
653.	C. D. Bourguet, V.; Gobert, M.; Durand, D.; Boissy, A.; Terlouw, E. M. C.	2010	Characterising the emotional reactivity of cows to understand and predict their stress reactions to the slaughter procedure	Applied Animal Behaviour Science	10.1016/j.applanim.2010.03.008
654.	H. Benchaoui	2010	Population medicine and control of epidemics	Handbook of experimental pharmacology	
655.	C. Bähler; G. Regula; M. H. Stoffel; A. Steiner; A. von Rotz	2010	Effects of the two production programs 'Naturafarm' and 'conventional' on the prevalence of non-perforating abomasal lesions in Swiss veal calves at slaughter	Research in veterinary science	
656.	C. Alexandrescu Daniela	2010	ASPECTS REGARDING ORGANIC CATTLE BEEF TRANSPORTATION AND SLAUTHERING	Annals : Food Science and Technology, Vol 11, Iss 2, Pp 79-	
657.	M. R. Alam; N. G. Gregory; M. S. Uddin; M. A. Jabbar; S. Chowdhury; N. C. Debnath	2010	Frequency of nose and tail injuries in cattle and water buffalo at livestock markets in Bangladesh	Animal Welfare	

658.	M. R. Alam; N. G. Gregory; M. A. Jabbar; M. S. Uddin; J. P. Widdicombe; A. Kibria; M. S. I. Khan; A. Mannan	2010	Frequency of dehydration and metabolic depletion in cattle and water buffalo transported from India to a livestock market in Bangladesh	Animal Welfare	
659.	M. R. Alam; N. G. Gregory; M. A. Jabbar; M. S. Uddin; A. S. M. G. Kibria; A. Silva-Fletcher	2010	Skin injuries identified in cattle and water buffaloes at livestock markets in Bangladesh	The Veterinary record	
660.	L. Akhmad Rifai	2010	Prospect of Cattle Development to Support Self Sufficiency of Meat in North Sumatera Province	Wartazoa, Vol 20, Iss 2, Pp 85-	
661.	I. Ademola Adeshupo; F. Folorunso Oludayo	2010	Socio-economic implications of bovine liver rejection in a major abattoir in south-western Nigeria	Revista de Ciências Agrárias, Vol 33, Iss 2, Pp 211-	
662.	U. Yildiz; M. Saatci	2009	An Evaluation of the Welfare in the Large and Small Animal Transportations Made from Sarikamis	Kafkas Universitesi Veteriner Fakultesi Dergisi	
663.	J. Withee	2009	Streamlined Analysis for Evaluating the Use of Preharvest Interventions Intended to Prevent Escherichia coli O157:H7 Illness in Humans	Foodborne pathogens & disease. 2009 Sept., v. 6, no. 7	
664.	B. J. White	2009	Associations of beef calf wellness and body weight gain with internal location in a truck during transportation	Journal of animal science. 2009 Dec., v. 87, no. 12	
665.	J. Van Wart; R. K. Perrin	2009	Understanding Spatial Welfare Impacts of a Grain Ethanol Plant		
666.	K. Uetake; T. Ishiwata; T. Tanaka; S. Sato	2009	Physiological responses of young cross-bred calves immediately after long-haul road transportation and after one week of habituation	Animal Science Journal	10.1111/j.1740-0929.2009.00693.x
667.	A. C. M. Strappini, J. H. M.; Gallo, C. B.; Kemp, B.	2009	Origin and assessment of bruises in beef cattle at slaughter	Animal : an international journal of animal bioscience	
668.	B. Schnettler	2009	Consumer willingness to pay for beef meat in a developing country: The effect of information regarding country of origin, price and animal handling prior to slaughter	Food quality and preference. 2009 Mar., v. 20, no. 2	
669.	M. Schimanski	2009	Im Dritten Reich darf es keine Tierquälerei mehr geben"--Die Entstehung des Reichstierschutzgesetzes von 1933"	DTW. Deutsche tierarztliche Wochenschrift	
670.	C. J. Phillips; J. Wojciechowska; J. Meng; N. Cross	2009	Perceptions of the importance of different welfare issues in livestock production	Animal	10.1017/s1751731109004479

671.	J. C. Petherick; C. J. C. Phillips	2009	Space allowances for confined livestock and their determination from allometric principles	Applied Animal Behaviour Science	10.1016/j.applanim.2008.09.008
672.	J. C. Petherick; V. J. Doogan; B. K. Venus; R. G. Holroyd; P. Olsson	2009	Quality of handling and holding yard environment, and beef cattle temperament: 2. Consequences for stress and productivity	Applied Animal Behaviour Science	10.1016/j.applanim.2009.05.009
673.	A. Perillo; O. Paciello; A. Tinelli; A. Morelli; C. Losacco; A. Troncone	2009	Lesions associated with mineral deposition in the lymph nodes and lungs of cattle: a case-control study of environmental health hazard	Folia Histochem Cytobiol	10.2478/v10042-008-0119-z
674.	W. J. Nauta; T. Baars; H. W. Saatkamp; D. Weenink; D. Roep	2009	Farming strategies in organic dairy farming<br><br>Effects on breeding goal and choice of breed. An explorative study	Livestock Science	
675.	S. Millman	2009	Animal WelfareScientific Approaches to the Issues	Journal of Applied Animal Welfare Science	10.1080/10888700902719591
676.	D. J. Mellor; T. J. Gibson; C. B. Johnson	2009	A re-evaluation of the need to stun calves prior to slaughter by ventral-neck incision : an introductory review	N Z Vet J	10.1080/00480169.2009.36881
677.	D. McClenahan; K. Hillenbrand; A. Kapur; D. Carlton; C. Czuprynski	2009	Effects of extracellular ATP on bovine lung endothelial and epithelial cell monolayer morphologies, apoptoses, and permeabilities	Clin Vaccine Immunol	10.1128/cvi.00282-08
678.	N. Mach; A. Bach; M. Devant	2009	Effects of crude glycerin supplementation on performance and meat quality of Holstein bulls fed high-concentrate diets	J Anim Sci	10.2527/jas.2008-0987
679.	J. Lutze	2009	Monitoring of pesticides and veterinary drugs in Australian cattle: verification of the residue control system	Food additives & contaminants. Part B, Surveillance. 2009 Nov., v. 2, no. 2	
680.	A. B. Kudahl; S. S. Nielsen	2009	Effect of paratuberculosis on slaughter weight and slaughter value of dairy cows	J Dairy Sci	10.3168/jds.2009-2039
681.	D. G. Kennedy; H. D. Shortt; S. R. H. Crooks; P. B. Young; H. J. Price; W. G. Smyth; S. A. Hewitt	2009	Occurrence of alpha- and beta-nortestosterone residues in the urine of injured male cattle	Food Additives and Contaminants Part a-Chemistry Analysis Control Exposure & Risk Assessment	10.1080/02652030802662738
682.	T. Herva; A. M. Virtala; A. Huuskonen; H. W. Saatkamp; O. Peltoniemi	2009	On-farm welfare and estimated daily carcass gain of slaughtered bulls	Acta Agriculturae Scandinavica Section A-Animal Science	
683.	T. Herva; O. Peltoniemi; A. M. Virtala	2009	Validation of an Animal Needs Index for cattle using Test Theory	Animal Welfare	
684.	N. G. Gregory; T. Benson; C. W. Mason	2009	Cattle handling and welfare standards in livestock markets in the UK	Journal of Agricultural Science	10.1017/s0021859609008508

685.	N. G. Gregory	2009	Blood in the respiratory tract during slaughter with and without stunning in cattle	Meat science. 2009 May, v. 82, no. 1	
686.	K. G. Gouveia; P. G. Ferreira; J. C. R. da Costa; P. Vaz-Pires; P. M. da Costa	2009	Assessment of the efficiency of captive-bolt stunning in cattle and feasibility of associated behavioural signs	Animal Welfare	
687.	F. Gottardo; M. Brscic; B. Contiero; G. Cozzi; I. Andrighetto	2009	Towards the creation of a welfare assessment system in intensive beef cattle farms	Italian Journal of Animal Science	10.4081/ijas.2009.s1.325
688.	A. Girolami; A. Braghieri; A. Sodo; F. Napolitano; G. Maiorano	2009	Acceptability and intramuscular collagen properties of Podolian beef as affected by ageing	Italian Journal of Animal Science	10.4081/ijas.2009.s2.498
689.	T. J. Gibson; C. B. Johnson; J. C. Murrell; S. L. Mitchinson; K. J. Stafford; D. J. Mellor	2009	Amelioration of electroencephalographic responses to slaughter by non-penetrative captive-bolt stunning after ventral-neck incision in halothane - anaesthetised calves	N Z Vet J	10.1080/00480169.2009.36885
690.	T. J. Gibson; C. B. Johnson; J. C. Murrell; S. L. Mitchinson; K. J. Stafford; D. J. Mellor	2009	Electroencephalographic responses to concussive non-penetrative captive-bolt stunning in halothane - anaesthetised calves	N Z Vet J	10.1080/00480169.2009.36884
691.	T. J. Gibson; C. B. Johnson; J. C. Murrell; C. M. Hulls; S. L. Mitchinson; K. J. Stafford; A. C. Johnstone; D. J. Mellor	2009	Electroencephalographic responses of halothane - anaesthetised calves to slaughter by ventral-neck incision without prior stunning	N Z Vet J	10.1080/00480169.2009.36882
692.	T. J. Gibson; C. B. Johnson; J. C. Murrell; J. P. Chambers; K. J. Stafford; D. J. Mellor	2009	Components of electroencephalographic responses to slaughter in halothane - anaesthetised calves : effects of cutting neck tissues compared with major blood vessels	N Z Vet J	10.1080/00480169.2009.36883
693.	A. D. Fisher; I. G. Colditz; C. Lee; D. M. Ferguson	2009	The influence of land transport on animal welfare in extensive farming systems	Journal of Veterinary Behavior-Clinical Applications and Research	10.1016/j.jveb.2009.03.002
694.	S. Evangelia N; B. D. M; C. L. T; G. R; G. G; S. C. E	2009	WELFARE ASPECTS OF THE LONG DISTANCE TRANSPORTATION OF CATTLE	Scientific Papers Animal Science and Biotechnologies, Vol 42, Iss 2, Pp 613-	
695.	M. K. Edge; J. L. Barnett	2009	Development of animal welfare standards for the livestock transport industry: process, challenges, and implementation	Journal of Veterinary Behavior-Clinical Applications and Research	10.1016/j.jveb.2009.07.001

696.	J. Denaburski; A. Tworkowska	2009	The problem of pain in farm animals and its effects on animal welfare and certain economic results	Polish Journal of Veterinary Sciences	
697.	L. N. Costa	2009	Short-term stress: the case of transport and slaughter	Italian Journal of Animal Science	
698.	G. Cima	2009	HSUS calls for more rules after calf abuse allegations	Journal of the American Veterinary Medical Association	
699.	E. Canali; L. Keeling	2009	Welfare Quality (R) project: from scientific research to on farm assessment of animal welfare	Italian Journal of Animal Science	10.4081/ijas.2009.s2.900
700.	M. Berta Schnettler; F. Roberto Silva; B. Néstor Sepúlveda	2009	Utility to Consumers and Consumer Acceptance of Information on Beef Labels in Southern Chile Utilidad y Aceptación de Información en el Etiquetado de la Carne Bovina en Consumidores del Sur de Chile	Chilean Journal of Agricultural Research, Vol 69, Iss 3, Pp 373-	
701.	J. L. Barnett; P. H. Hemsworth	2009	Welfare Monitoring Schemes: Using Research to Safeguard Welfare of Animals on the Farm	Journal of Applied Animal Welfare Science	10.1080/10888700902719856
702.	L. N. Costa; D. P. Lo Fiego; F. Tassone; V. Russo	2006	The relationship between carcass bruising in bulls and behaviour observed during pre-slaughter phases	Veterinary Research Communications	10.1007/s11259-006-0086-9
