# Supplementary material for: Animal Welfare during Transport and Slaughter of Cattle: A Systematic Review of Studies in the European Legal Framework
Source: Animals (Basel). 2023 Jun 13;13(12):1974. doi: 10.3390/ani13121974 (PMC10295209; doi:10.3390/ani13121974)
Supplement: Supplementary file 1 [file animals-13-01974-s001.zip › S4.pdf]

Animal welfare during transport and slaughter of cattle: a systematic review of studies in the European legal framework

S4: List of included publications

1. Dahl-Pedersen, K.; Herskin, M.S.; Houe, H.; Thomsen, P.T. Risk factors for deterioration of the clinical condition of cull dairy cows during transport to slaughter. *Front. Vet. Sci.,* **2018**, 5, 297.
2. Bourguet, C.; Deiss, V.; Tannugi, C.C.; Terlouw, E.M.C. Behavioural and physiological reactions of cattle in a commercial abattoir: relationships with organisational aspects of the abattoir and animal characteristics. *Meat Sci.*, **2011**, 88, 158-168.
3. Hultgren, J.; Wiberg, S.; Berg, C.; Cvek, K.; Kolstrup, C.L. Cattle behaviours and stockperson actions related to impaired animal welfare at Swedish slaughter plants. *Appl. Anim. Behav. Sci.*, **2014**, 152, 23-37.
4. Hultgren, J.; Segerkvist, K.A.; Berg, C.; Karlsson, A.H.; Algers, B. Animal handling and stress-related behaviour at mobile slaughter of cattle. *Preventive Veterinary Medicine*, **2020**, 17.
5. Probst, J.K.; Neff, A.S.; Hillmann, E.; Kreuzer, M.; Koch-Mathis, M.; Leiber, F. Relationship between stress-related exsanguination blood variables, vocalisation, and stressors imposed on cattle between lairage and stunning box under conventional abattoir conditions. *Livest. Sci.,* **2014**, 164, 154-158.
6. Bourguet, C.; Deiss, V.; Gobert, M.; Durand, D.; Boissy, A.; Terlouw, E.M.C. Characterising the emotional reactivity of cows to understand and predict their stress reactions to the slaughter procedure. *Appl. Anim. Behav. Sci.*, **2010**, 125, 9-21.
7. Disanto, C.; Celano, G.; Varvara, M.; Fusiello, N.; Fransvea, A.; Bozzo, G.; Celano G. Stress factors during cattle slaughter. *Ital. J. Food Saf.*, **2014,** 3, 143-144.
8. Iulietto, M.F.; Sechi, P.; Gaudenzi, C.M.; Grispoldi, L.; Ceccarelli, M.; Barbera, S.; Cenci-Goga, B.T. Noise assessment in slaughterhouses by means of a smartphone app. *Ital. J. Food Saf.*, **2018,** 7, 79-82.
9. Fries, R.; Schrohe, K.; Lotz, F.; Arndt, G. Application of captive bolt to cattle stunning - A survey of stunner placement under practical conditions. *Animal,* **2012,** 6, 1124-1128
10. Vecerek, V.; Kamenik, J.; Voslarova, E.; Volfova, M.; Machovcova, Z.; Konvalinova, J.; Vecerkova, L. The impact of deviation of the stun shot from the ideal point on motor paralysis in cattle. *Animals,* **2020,** 10.
11. Grist, A. Macroscopic examination of multiple-shot cattle heads - An animal welfare due diligence tool for abattoirs using penetrating captive bolt devices? *Animals,* **2019,** 9, 328.
12. von Wenzlawowicz, M.; von Holleben, K.; Eser, E. Identifying reasons for stun failures in slaughterhouses for cattle and pigs: A field study. *Anim. Welf.*, **2012**, 21, 51–60.
13. Verhoeven, M.T.; Gerritzen, M.A.; Hellebrekers, L.J.; Kemp, B. Validation of indicators used to assess unconsciousness in veal calves at slaughter. *Animal,* **2016**, 10, 1457-1465.
14. Terlouw, C.; Bourguet, C.; Deiss, V. Consciousness, unconsciousness, and death in the context of slaughter. Part II. Evaluation Methods. *Meat Sci.,* **2016,** 118, 147-156.
15. Verhoeven, M.T.; Gerritzen, M.A.; Hellebrekers, L.J.; Kemp, B. Indicators used in livestock to assess unconsciousness after stunning: a review. *Animal,* **2015**, 9 320-330.
16. Atkinson, S.; Velarde, A.; Algers, B. Assessment of stun quality at commercial slaughter in cattle shot with captive bolt. *Anim. Welf.,* **2013**, 22, 473-481.
17. Borzuta, K.; Lisiak, D.; Janiszewski, P.; Grzeskowiak, E. The physiological aspects, technique and monitoring of slaughter procedures and their effects on meat quality - A review. *Ann. Anim. Sci.*, **2019**, 19, 857-873.
18. Vecerek, V.; Kamenik, J.; Voslarova, E.; Vecerkova, L.; Machovcova, Z.; Volfova, M.; Konvalinova, J. The occurrence of reflexes and reactions in cattle following stunning with a captive bolt at the slaughterhouse. *Anim. Sci. J,* **2020,** 91.

19. Herskin, M.S.; Hels, A.; Anneberg, I.; Thomsen, P.T. Livestock drivers' knowledge about dairy cow fitness for transport – A Danish questionnaire survey. Res. Vet. Sci., **2017**, 113, 62-66.
